# Material Hardship Across Metropolitan and Nonmetropolitan Counties in the United States: 2013–2021

**DOI:** 10.1111/ruso.70028

**Published:** 2025-11-11

**Authors:** Michael Caniglia

**Affiliations:** Department of Sociology and Criminology, The Pennsylvania State University, University Park, Pennsylvania, USA

## Abstract

Using data from the Survey of Income and Program Participation (SIPP) (*N* = 69,735), this paper analyzes wellbeing across nonmetropolitan–metropolitan areas through the lens of material hardship—characterized here as a household’s inability to purchase sufficient healthy foods, live in structurally safe homes, or pay bills. This approach mirrors that of other social scientists who depict poverty as not only the absence of income, but also the inability to live well. Bivariate linear probability models indicate that nonmetropolitan households face substantively greater risks of not securing enough healthy food (*b* = 0.026, *p* < 0.001), living in a poor-quality housing unit (*b* = 0.014, *p* < 0.05), and experiencing any form of hardship (*b* = 0.020, *p* < 0.05) relative to metropolitan households. However, disparities disappear after accounting for demographic and household characteristics. In the case of bill-paying hardship, nonmetropolitan households exhibit lower risks of hardship than metropolitan settings in the final models (*b* = −0.009, *p* < 0.05). Decomposition models indicate that lower incomes and elevated prevalence of disability among nonmetropolitan households explain many of the initial disparities evidenced in bivariate models.

## 1 | Introduction

Beginning in the 1970s, globalization, technological development, and deindustrialization transformed the spatial distribution of economic prosperity in the United States (Edin et al. 2023; Lobao and Meyer 2001). The changes advantaged metropolitan (metro) areas, which were home to finance and information hubs (Le Galès 2024), while nonmetropolitan (nonmetro) areas lagged in job creation (Pender 2019).^1^ Measures comparing economic security among metro households relative to nonmetro households could inform sociologists’ understanding of metro status as a social dividing line (Cramer 2016).

Yet, one of the nation’s most important metrics of population wellbeing—the poverty rate—varies by how it is measured. The US Census Bureau’s Official Poverty Measure (OPM) uses an absolute income threshold that is pegged to inflation and adjusts for family size and age composition (Iceland and Bauman 2007). In 2023, the OPM indicated that 13.5% of individuals in nonmetro areas lived below the poverty threshold compared to 10.7% of metro residents (Shrider 2024). In that same year, the Census Bureau’s Supplemental Poverty Measure (SPM)—which does a more refined job of accounting for family resources and needs—indicated that 11.9% of nonmetro and 13.0% of metro residents were poor (Shrider 2024).

Although the OPM and SPM provide important insights, the measures’ differing estimates of spatial differences muddy the understanding of wellbeing across metro status. This study provides an additional perspective on US poverty by examining metro–nonmetro variation in the prevalence of material hardship, defined here as the inability to purchase healthy foods, pay essential bills, or live in quality housing (Iceland 2021; Iceland and Sakamoto 2022).

Although they are similar, poverty and material hardship are distinct theoretical concepts. Amartya Sen (1999) perceived income as a resource that provides individuals with the ability to meet basic needs, but he recognized that income resources might not always translate to consumption. Similarly, Ringen (1988) referred to material hardship indicators as direct poverty measures because of their focus on meeting basic needs, while income-based poverty measures acted as indirect indicators of poverty status.

The unique perspectives of income-based poverty measures and hardship indicators prevent perfect correlation. For instance, not all individuals who experience material hardship have earnings below the poverty line (Iceland and Bauman 2007). This may prove true for households that have high living expenses or those who provide financial assistance to family members outside the home (Campbell 2021). Meanwhile, not all households with incomes below the poverty line face difficulty meeting basic needs. Some may benefit from personal savings, credit access, or resources provided by social networks (Campbell 2021). Place-based factors may further distort the association between income and material hardship. Income poverty measures do not consider informal provisioning activities, such as gardening or hunting, that occur more frequently in nonmetro areas and influence households’ abilities to meet basic needs (Jensen et al. 2019).

The material hardship measures presented here augment ongoing debates about metro and nonmetro poverty differences (Mueller et al. 2022; Pacas and Rothwell 2020). This study builds on descriptive findings by accounting for population-level characteristics that may differ across metro and nonmetro areas and explain disparities. Analyses use a recent, national survey dataset to focus renewed attention on variation in food, housing quality, and bill-paying hardships by metro status (Heflin et al. 2009; Iceland 2021). It offers a clearer understanding of the lived experiences of poverty in nonmetro and metro areas than if using income-based measures alone (Iceland and Bauman 2007).

## 2 | Background

From the 1900s through the early 2000s, nonmetro areas faced a series of economic challenges (Lobao and Meyer 2001; Thiede et al. 2018; Wuthnow 2018). At the start of the 20th century, the mechanization of agriculture reduced the need for farm workers and generated surplus labor. In search of lower wages, businesses moved many of their manufacturing operations to nonmetro areas with loose labor markets in the 1970s (Slack and Monnat 2024). Many of the same corporations further reduced costs by transitioning production from nonmetro settings to developing countries (Carr and Kefalas 2009; Slack and Monnat 2024). Service sector jobs with low wages and few benefits replaced manufacturing positions in nonmetro areas (Thiede et al. 2018). In 2019 alone, median earnings per worker were $42,659 and $35,478 in metro and nonmetro areas, respectively (Farrigan 2023). Simultaneously, financial crises rocked small farms, and mining companies laid off workers (Lobao and Meyer 2001; Wuthnow 2018).

Sociologists have long posited that economic processes, government institutions, and geographic characteristics shape spatial stratification (Lobao 2004; Lobao et al. 2007; Lovejoy and Krannich 1982). Rural dependency theory offers one way to conceptualize the economic relationships between metro and nonmetro residents. The theory depicts metro residents as extracting resources and labor from outlying populations. It suggests that nonmetro residents experience little economic benefit from metro dominance (Lovejoy and Krannich 1982; Mueller 2020). For instance, even as metro settings rely on food and energy from nonmetro communities, nonmetro workers face chronically low wages in precarious positions (Slack and Monnat 2024; Thiede et al. 2018). Although it is difficult to test, dependency theory offers a way to consider how economic structures in nonmetro communities are associated with their households’ relative difficulty meeting basic needs compared to those in metro areas (Mueller 2020).

Institutions and geographic characteristics further shape variation in the prevalence of material hardship through their interactions with households. Nonmetro governments frequently face shrinking tax bases that make it more difficult to provide services for residents at risk of material hardship, while nonprofits struggle to support disadvantaged clients (Lobao 2004; Shapiro 2021). Relative to their metro peers, nonmetro residents are more likely to use geographic features such as soil, game, and timber to meet their basic needs (Jensen et al. 2019). Yet, distance from population centers and small populations can be associated with higher prices and fewer economic opportunities (Miller et al. 2022; Slack and Monnat 2024).

### 2.1 | Material Hardship in Nonmetro and Metro Households

The relationship between metro status and each form of material hardship may vary based on differences in geography, economic processes, and institutional characteristics. In the space below, I discuss how these factors apply to food, bill-paying, and housing quality hardships. In each case, I pose competing hypotheses.

#### 2.1.1 | Food

The literature provides varying perspectives regarding the association between metro status and food hardship. Research using data from the Great Recession and adjusting for respondent income and demographic characteristics suggests that nonmetro areas had significantly lower levels of food hardship than their metro peers (Coleman-Jensen 2012; Heflin 2017). More recently, descriptive statistics from the US Department of Agriculture find that nonmetro areas have higher rates of food hardship (14.7%) relative to their metro peers (12.5%) (Rabbitt et al. 2023). The differences may reflect variation in demographic and income characteristics across metro and nonmetro areas.

Structural economic forces may also contribute to food hardship in nonmetro areas. Despite hosting the nation’s breadbasket, nonmetro America as a whole has witnessed a decline in the number of food retailers since 2010 (Stevens et al. 2021). The absence of local grocery stores forces nonmetro residents to travel farther to access food (Stevens et al. 2021). When residents arrive at a nonmetro grocery store, they often find higher food prices than in metro centers because of the limited market competition in isolated areas (Miller et al. 2022). Thus, I make the following hypothesis:

**Hypothesis 1a.** *Nonmetro households are more likely to face food hardship than metro households*.

On the other hand, nonmetro populations may use place-based strategies to avoid food hardship. For example, informal provisioning via gardening and hunting may partially support nonmetro households’ food budgets. Higher levels of home ownership and greater access to natural resources in nonmetro areas may allow some households to save money on vegetables harvested from a backyard garden or deer meat from hunting (Jensen et al. 2019). One study found that nonmetro households (62%) are more likely than their metro peers (57%) to report using informal provisioning to make ends meet during economically difficult periods (Jensen et al. 2019).

Despite higher food prices and longer trips to grocery stores, informal provisioning may reduce the risk of food hardship for nonmetro households. These factors lead me to the following hypothesis:

**Hypothesis 1b.** *Nonmetro households are less likely to experience food hardship than metro households*.

#### 2.1.2 | Bill-Paying

Individuals living in nonmetro areas may face unique financial pressures when paying essential household bills, namely utilities and rent. One study found that a greater percentage of households in nonmetro areas experienced power shutoffs as a result of not paying utility bills, but the results were not significant after controlling for income, racial identity, and residence in a manufactured housing unit (Hernández and Laird 2022). Nonmetro households may face worse insulation and higher utility bills because of the unique nature of prevailing housing stocks. For instance, more than three million American households reside in manufactured housing units in nonmetro areas, making up 14% of nonmetro households compared to 6% nationwide (Durst and Sullivan 2019). Residents living in mobile homes and other manufactured housing often pay higher utility bills due to poor insulation (Salamon and MacTavish 2017). The research above supports the following hypothesis:

**Hypothesis 2a.** *Nonmetro households are more likely to suffer bill-paying hardships than metro households*.

Despite the potential for higher costs, some evidence indicates that nonmetro households face fewer difficulties paying for housing relative to their metro peers. Heflin (2017) found that nonmetro households were less likely to report missing a mortgage or rent payment in the past year compared to metro households in 2010 and 2011. Fewer missed housing payments may reflect reduced housing costs in nonmetro settings (Heflin 2017).

Characteristics of nonmetro areas may contribute to a bill-paying affordability advantage. For example, manufactured housing units are a frequent source of affordable housing in nonmetro areas (Salamon and MacTavish 2017). The savings associated with living in a manufactured housing unit relative to other housing types may ease strains on the budgets of nonmetro households at greatest risk for material hardship. Nonmetro residents may also save money on utilities by using natural resources to power their homes. Nonmetro households are more likely to use wood-burning fireplaces to warm their homes than their metro counterparts (Fitchen 1981; Walker et al. 2021). Given these factors, I make the following alternative hypothesis:

**Hypothesis 2b.** *Nonmetro households face fewer bill-paying hardships than metro households*.

#### 2.1.3 | Housing Quality

Existing scholarship indicates that nonmetro areas may face greater hardship associated with housing quality. Nationally representative survey results suggest that individuals located in the most isolated nonmetro areas find mice or rats in the home at twice the rate of metro households (21%–9%) (Yam et al. 2024). Respondents in the most unpopulous nonmetro areas also report higher prevalences of missing roof components (4.4%–2.0%) and broken windows (4.5%–2.3%) compared to metro households (Yam et al. 2024).

The variation in housing quality across metro and nonmetro communities reflects differences in their housing stocks and institutional capacities. As noted, manufactured housing is more prevalent in nonmetro communities and is especially prone to structural decay over time (Salamon and MacTavish 2017). At the same time, institutional challenges play a role. Nonmetro communities frequently lack the administrative capacity to enforce housing codes that regulate housing quality in metro settings (Skobba et al. 2020). These factors motivate my third hypothesis:

**Hypothesis 3a.** *Nonmetro households are more likely to face housing quality hardship than metro households*.

In the face of ongoing housing quality challenges, nonmetro households often engage in self-organized home improvement projects. Nonmetro residents are significantly more likely to report participation in home improvement projects than their metro counterparts (28%–23%) (Jensen et al. 2019). Do-it-yourself repair skills serve as an important source of social capital in nonmetro settings and can save households money (Fitchen 1981; Halpern-Meekin and Talkington 2022). Because of these characteristics of nonmetro settings, I make the following alternative hypothesis.

**Hypothesis 3b.** *Nonmetro households are less likely to experience housing quality hardship than metro households*.

#### 2.1.4 | Experiencing Any Hardship

Because of the interconnected nature of material hardship types, an indicator of whether households experienced any of the three domains offers the broadest measure of nonmetro and metro disparities (Campbell et al. 2022). A measure of whether a household experiences any material hardship may also depict the overall responsiveness of local institutions to community needs. In metro areas, large nonprofits help individuals who might not qualify for government assistance (Shapiro 2021). However, the nation’s most geographically isolated counties have the lowest levels of nonprofit human services spending per capita (Shapiro 2021). The lack of institutional capacity in nonmetro communities relative to metro areas suggests another hypothesis.

**Hypothesis 4a.** *Nonmetro households are more likely to experience any form of material hardship than metro households*.

Other characteristics of nonmetro households may reduce the overall risk of experiencing material hardship. Informal provisioning may play a significant role in addressing hardships (Fitchen 1981; Jensen et al. 2019). In addition, evidence indicates that nonmetro families more freely share resources among kin relative to their counterparts in metro settings (Hofferth and Iceland 1998). Reallocating resources among family members may serve as a strategy to overcome the relative absence of nonprofit services in nonmetro settings (Hofferth and Iceland 1998). The above factors motivate the hypothesis below.

**Hypothesis 4b.** *Nonmetro households are less likely to experience any form of material hardship than metro households*.

### 2.2 | Demographic and Household Factors as Sources of Hardship Disparities by Metro Area

Demographic and household characteristics play important roles in shaping the risk of material hardships among US households (Heflin 2017; Iceland 2021). Variation in population composition may partially explain the differences in rates of material hardships among metro and nonmetro households. Identifying the specific population features that widen or narrow hardship disparities by metro status could offer insight into how to best address the gaps.

For instance, nonmetro households tend to have lower household incomes than metro settings (Guzman and Kollar 2023; Slack and Monnat 2024). The largest sectors in nonmetro economies, such as the service industry and agriculture, typically pay low wages (Thiede et al. 2018). Nonmetro areas also have reduced employment rates and an elevated prevalence of individuals reporting a disability (Slack and Monnat 2024). For these reasons, I make the hypothesis below:

**Hypothesis 5.** *Elevated rates of disability and lower incomes among nonmetro households promote disadvantages in material hardship prevalence relative to metro households*.

## 3 | Data and Methods

This study uses data from the US Census Bureau’s Survey of Income and Program Participation (SIPP). The SIPP, which is representative of the US non-institutionalized population, asks respondents detailed questions regarding their income, receipt of public programs, use of nonprofit services, and ability to meet basic needs associated with food, housing, and utilities. During a single panel, SIPP respondents are surveyed annually over 4 years, or waves, on topics involving their income and safety-net use over the previous calendar year (US Census Bureau 2019). At the conclusion of a panel, SIPP onboards a new set of respondents.

In my analysis, I utilize cross-sectional data from the SIPP’s 2014, 2018, and 2022 annual waves. Survey results reflect the previous year (referred to as the reference year), so the dataset includes information on calendar years 2013, 2017, and 2021. The years used in this study are optimal for several reasons. First, beginning with the 2014 panel, a major redesign shifted the SIPP’s data collection practices, so this study only uses panels surveyed under the new structure (US Census Bureau 2019). Second, the federal government adjusts its definitions of metro and nonmetro status every decade (Office of Management and Budget 2013). The SIPP waves analyzed here use the same definition of metro status (US Census Bureau 2019). Third, the use of the 2014, 2018, and 2022 waves ensures that each cross section includes a completely new set of respondents. The use of cross-sectional data eases concerns surrounding survey attrition, which has grown in recent years (US Census Bureau 2019). Since responses related to material hardship are reported at the household level, the sample is limited to the universe of householder respondents participating in 2014, 2018, and 2022 (*N* = 73,348). Householders are those who own the residence or pay rent on behalf of the household (US Census Bureau 2019). There is one householder per household.

The Census Bureau seeks to prevent geographic indicators from disclosing a respondent’s identity. Specifically, the SIPP provides a household’s metro status when it is in a state with at least 250,000 residents in both nonmetro and metro areas (US Census Bureau 2019). The SIPP also provides metro status information when a state’s population is either completely made up of metro or nonmetro residents (US Census Bureau 2019). I drop households without metro status information from my sample (*N* = 3613).^2^ The Census Bureau imputes missing responses to all other items prior to releasing SIPP data to the public (US Census Bureau 2019). As a result, no study variables have missing data. The final sample constituted 69,735 householders interviewed in one of the 2014, 2018, or 2022 SIPP waves.

### 3.1 | Outcomes

I analyze three categories of material hardship. To enhance comparability of results across studies, I utilize composite measures that reflect a broad set of hardship experiences and align with those employed by other studies (Heflin 2017; Iceland and Sakamoto 2022). Specifically, I identify the presence of material hardship within each category in the previous year using responses to a series of related questions. For instance, to categorize a household as experiencing *food hardship*, the householder must agree with at least two of the following statements: often or sometimes ran out of food and didn’t have money to buy more; often or sometimes could not afford balanced meals; ate less than should have because of costs; cut size of or skipped meals because of costs; or was hungry and didn’t eat because there wasn’t money to pay for food. Next, I categorize *bill-paying hardship* as occurring if the householder reported an inability to pay rent, mortgage, or utility bills. I define *housing quality hardship* as encompassing an affirmative response to any of the following statements about the currently inhabited dwelling: cracks exist in the ceiling or walls; there are holes in the floor; there are problems with pests; and there are plumbing issues. (Please see Table S1 for complete survey questions.) I also developed a binary variable for *experienced any hardship* (i.e., any one of these three hardships).^3^

### 3.2 | Focal Variables

I use the Census Bureau’s definition of *metro status* (metro = 0, nonmetro = 1) to determine whether a household resided in a metro or nonmetro county in the previous year. Specifically, I identify metro status based on the householder’s location at the end of the calendar year preceding the survey response. The SIPP identifies a respondent’s metro status according to the county of residence (Office of Management and Budget 2013).

### 3.3 | Covariates

This analysis used an array of covariates to account for potential demographic and socioeconomic correlates of material hardship (Heflin 2017; Iceland 2021; Iceland and Sakamoto 2022). First, I included variables for *gender* (women = 1), *race and ethnicity* (non-Hispanic white, non-Hispanic Black, Hispanic, Asian, Other), and *nativity* (born in the US = 1) of the householder. I also control for the householder’s *educational attainment* (high school degree, GED, or less, some college, bachelor’s degree, more than a bachelor’s degree), *marital status* (never married; married; divorced, separated, or widowed), a *employment status* (employed, unemployed, not in the labor force), as well as *householder age* and *age-squared*. Other variables indicate whether a household includes a *child under age 18*, an *elderly person over age 65*, or a *person with a disability*. I use the Census Bureau’s broadest definition of disability—one that includes adults facing difficulties working or carrying out basic activities and children exhibiting behavioral or developmental challenges. Other covariates include *household tenure* (own, rent, occupy without rent), *region* (Northeast, Midwest, South, and West), and *reference year*, or the calendar year referenced by the survey. To account for the role of economic insecurity in predicting material hardship, I include household income to poverty ratio using the OPM’s threshold (< 1.00, 1.00–1.99, 2.00–2.99, 3.00–3.99, 4.00 or more). (Household income-to-poverty ratio is only modestly correlated with material hardship. See Tables S2–S4 for additional information.) The covariates utilized here more closely isolated the association between metro status and material hardship after accounting for demographic and household variation across metro and nonmetro spaces.

### 3.4 | Analytic Approach

First, I review descriptive statistics and examine differences in population means using two-sided *t*-tests. Second, bivariate linear probability models estimate the risk of experiencing each hardship type. Scholars frequently use linear probability models to estimate the risks of experiencing poverty-related binary outcomes (Laird et al. 2018). Third, multivariate linear probability models examine the risk of material hardship while accounting for all covariates except income-to-poverty ratio. Fourth, linear probability models add income-to-poverty ratios. Finally, I utilize Blinder–Oaxaca decompositions to estimate the proportion of material hardship gaps attributable to population differences (Jann 2008). All analyses use Stata 18 (2023), cluster standard errors by state, and employ SIPP’s survey weights.

### 3.5 | Supplementary Analyses

Multiple analyses investigate the sensitivity of results to study specifications. First, models test the sensitivity of results to missing data by using multiple imputation to estimate one set of models and control for missing metro status in another set. Second, because of the unique nature of the COVID-19 pandemic, I examine the sensitivity of results to excluding reference year 2021. Third, tests examine whether results remain similar when using scales based on the summation of responses to material hardship questions. Fourth, I examine whether results remain the same when using logistic regression. Finally, I check whether decomposition results differ when using the Fairlie decomposition method in place of the Blinder–Oaxaca approach (Jann 2008).

## 4 | Results

### 4.1 | Descriptive Results

Weighted descriptive statistics presented in Table 1 indicate that 14.8% of the sample lives in a nonmetro household, a number approximating the federal government’s estimates that 14%–15% of the US population resided in nonmetro households between 2010 and 2020 (Dobis et al. 2021). Nonmetro households report either the presence of an individual with a disability (46.5%–39.8%, *p* < 0.001) or a person over age 65 (33.3%–28.5%, *p* < 0.001) at higher rates than metro households. Almost half of nonmetro householders (47.8%) report a high school degree or less compared to approximately one-third (33.5%, *p* < 0.001) of their metro peers. Nonmetro householders are more likely to be non-Hispanic white (81.9%) relative to metro householders (63.4%, *p* < 0.001). Nonmetro households are more likely to report household income-to-poverty ratios below 1.0 (16.4%–13.4%, *p* < 0.001) and less likely to indicate ratios at or above 4.0 (29.3%–42.4%, *p* < 0.001) compared to metro households. Descriptive statistics presented in Table 2 suggest variation in the prevalence of material hardship components across nonmetro and metro households.

### 4.2 | Regression Results

**Figure 1.**
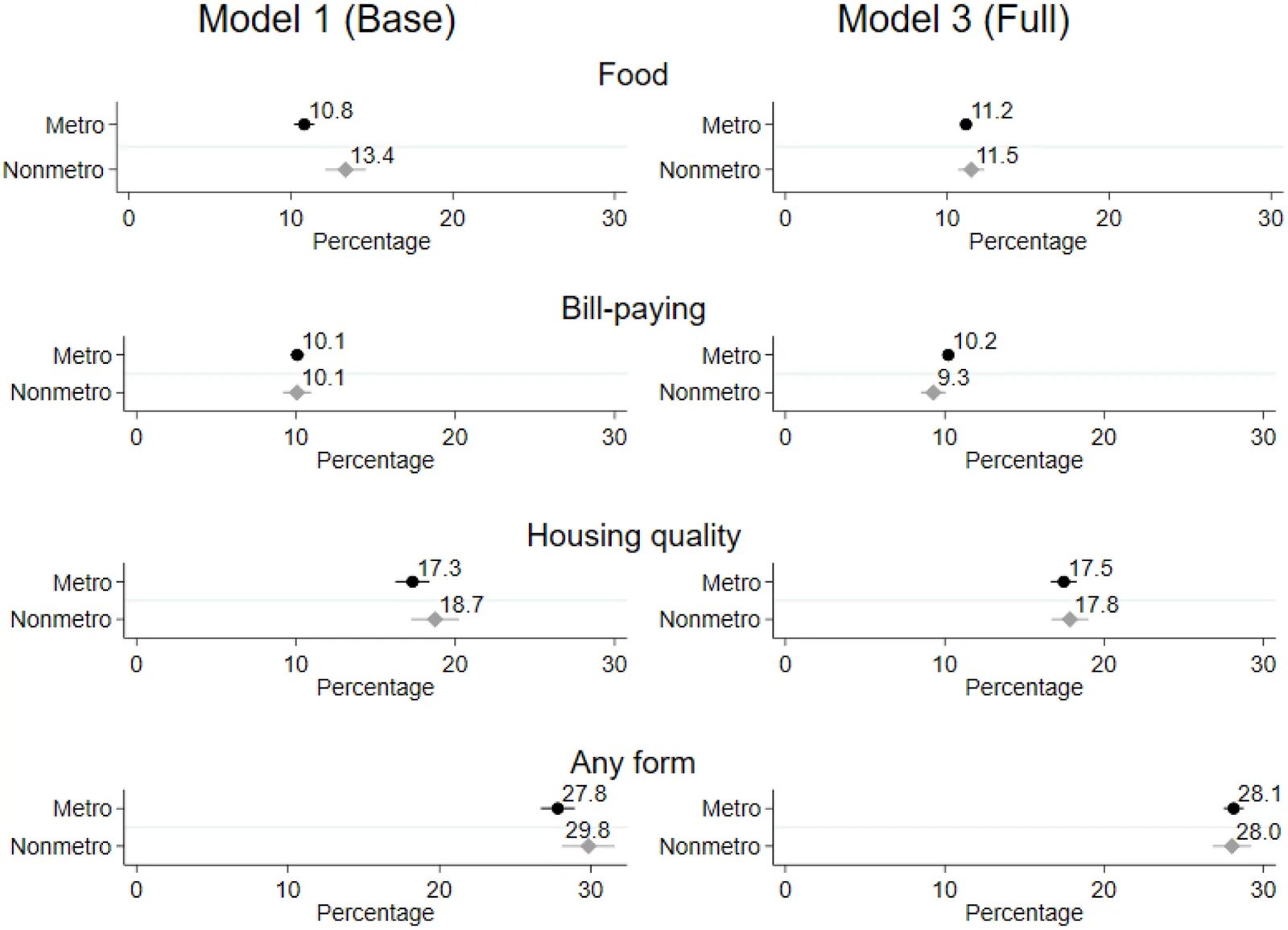
Predicted probabilities of households experiencing material hardship domains by metropolitan status and model specification. Notes: Predicted probabilities and 95% confidence intervals shown. Probabilities for the Model 1 (Base) are unadjusted. Model 3 (Full) probabilities account for householder characteristics (age, age-squared, gender, race and ethnicity, nativity, education attainment, marital status, and employment status), household characteristics (housing tenure, presence of a person with a disability, presence of a person 65 years or older, presence of a child, number of persons present, and income-to-poverty ratio), region, and reference year. Metro=Metropolitan Nonmetro=Nonmetropolitan Source: Survey of Income and Program Participation’s Waves 2014, 2018, and 2022

In Table 3, linear probability regression models investigate the association between metro status and food hardship and test competing Hypotheses 1a and 1b. Model 1 presents bivariate regression estimates in which nonmetro households are more likely to experience food hardship than their metro counterparts (*b* = 0.026, *p* < 0.001). The disadvantage declines with the inclusion of the main controls in Model 2 (*b* = 0.014, *p* < 0.01), and it disappears when adding household income-to-poverty ratios in Model 3 (*b* = 0.003). Figure 1 displays differences in the predicted probabilities of food hardship by metro status and model type.

Table 4 examines the association between metro status and bill-paying hardships. No differences existed in Model 1 (*b* = 0.000) and none emerged after including the main set of controls in Model 2 (*b* = −0.002). After adding household income-to-poverty ratio to Model 3, estimates suggest nonmetro households are less likely to experience bill-paying hardships (*b* = −0.009 *p* < 0.05). Outcomes in Table 4 support Hypothesis 2b, which posited that nonmetro households have lower risks of material hardship.

As noted in Table 5, Model 1 suggests that nonmetro residents have significantly greater risks of housing quality hardship than metro residents (*b* = 0.014, *p* < 0.05), but the association attenuates in Models 2 and 3 after accounting for key covariates (*b* = 0.009) and the addition of income-to-poverty ratios (*b* = 0.004), respectively. Given the null findings, neither Hypotheses 3a nor 3b are supported.

As with food and housing quality hardships, results presented in Table 6 indicate that nonmetro residents initially demonstrate an increased risk of experiencing any form of hardship (*b* = 0.020, *p* < 0.05), but the associations weaken in Models 2 (*b* = 0.012) and 3 (*b* = −0.001). The results do not support either Hypotheses 4a or 4b.

### 4.3 | Decomposition Results

**Figure 2.**
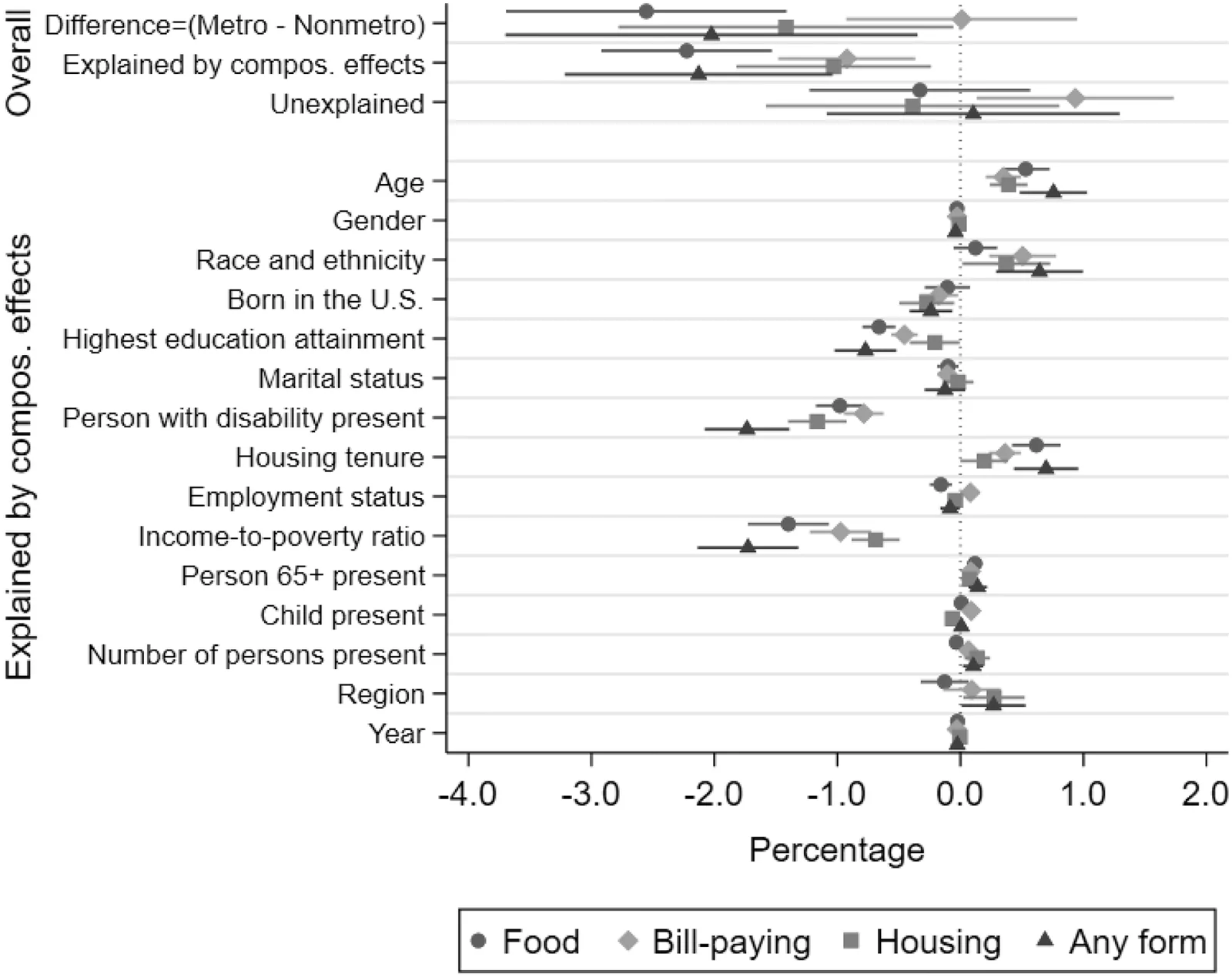
Blinder-Oaxaca decompositions of differences in the prevalence of material hardships across metropolitan and nonmetropolitan households. Notes: Metro=Metropolitan; Nonmetro=Nonmetropolitan; compos. effects =compositional effects Positive effects values reflect sources of nonmetropolitan advantage while negative values indicate areas of disadvantage compared to metropolitan settings. Source: Survey of Income and Program Participation Waves 2014, 2018, and 2022

Compositional effects, or the share of average differences related to differing population characteristics across metro and nonmetro households, explain half or more of the hardship differences. In Table 7, positive effects values reflect sources of nonmetro advantage while negative values indicate areas of disadvantage compared to metro settings. Results support Hypothesis 5. Specifically, if income-to-poverty ratios in nonmetro households were as high as metro households, nonmetro households would experience 0.014 (*p* < 0.001) lower probabilities of food hardship. Nonmetro households would also exhibit 0.01 (*p* < 0.001) lower risks of food hardship if they reported a person with a disability at the same rate as metro settings. Taken together, variation in household income-to-poverty ratios and disability rates explain 92% of the difference in food hardship across metro and nonmetro households [0.024 of 0.026]. As indicated in Figure 2, household income-to-poverty ratios and disability similarly contribute to nonmetro disadvantages in the categories of housing quality and any form of hardship.

### 4.4 | Supplementary Analyses

The main results remained robust to other model specifications. First, results do not change when analyses employ multiple imputation with chained equations to address unknown metro status or control for missing metro status (Tables S10–S18). Second, results remain similar when excluding data from 2021. These findings suggest that pandemic-era hardships alone do not account for disparities across metro status (Tables S19–S23). Third, the relationship between nonmetro residence and material hardship remains largely similar when using composite scales formed from material hardship questions in place of binary indicators for food, bill-paying, and housing quality hardships (Tables S24–S27). Fourth, results are robust to the use of logistic regression models (Tables S28–S32). Finally, the use of Fairlie decomposition models in place of Blinder–Oaxaca models does not substantively change results (Table S33).

## 5 | Discussion

This study’s findings improve rural sociologists’ understanding of material hardship disparities between metro and nonmetro households. The results underscore the importance of economic, institutional, and geographic characteristics in explaining material hardship differences between metro and nonmetro households. The outcomes carry implications for future research, theory, and policymaking.

Findings from this analysis indicate that nonmetro residence is associated with food hardship, housing quality hardship, and experiencing any form of hardship overall in the bivariate base models. Hypotheses 1a, 3a, and 4a, are supported in the base models as nonmetro areas exhibit significantly greater risks of material hardship than metro settings. However, disparities disappear after accounting for a range of demographic characteristics and income-to-poverty ratios. In fact, when using fully specified models inclusive of household income-to-poverty ratios, nonmetro areas exhibit lower risks of bill-paying hardships compared to their metro peers, supporting Hypothesis 2b. Decomposition estimates offer support for Hypothesis 5, which points to higher disability prevalence and lower incomes among nonmetro households as leading explanations for disadvantage. Each hardship measure provides a unique perspective into the possible economic, institutional, and geographic factors contributing to hardship differences.

Even as some methods depict nonmetro areas as experiencing lower costs of living relative to metro areas (Mueller et al. 2022; Pacas and Rothwell 2020), nonmetro households in this study experienced similar hardships accessing healthy food compared to peers in metro areas. In fully specified models using household income-to-poverty ratios, nonmetro counties displayed no greater probability of food hardship relative to metro settings. The null finding reflects a departure from earlier work that identified nonmetro areas as exhibiting lower risks of food hardship after adjusting for covariates (Coleman-Jensen 2012; Heflin 2017). Changing market factors, such as the closure of local grocery stores, could have played a role in the evolution of food hardship across metro and nonmetro areas since the early 2010s (Heflin 2017; Stevens et al. 2021). The food hardship results also suggest that informal food provisioning strategies rooted in local geographies might not overcome all the challenges facing nonmetro households (Coleman-Jensen and Steffen 2017).

Nonmetro residence predicted reduced risks of bill-paying hardship compared to metro settings in fully specified models. Residents’ budgets in nonmetro areas benefit from cheaper housing sources, such as manufactured housing, that minimize the size of monthly payments (Durst and Sullivan 2019; Salamon and MacTavish 2017). Previous researchers have found few differences in the risk of utility disconnections across metro status while identifying lower rates of court-ordered evictions in nonmetro areas (Gershenson and Desmond 2024; Hernández and Laird 2022).

In contrast to bill-paying hardship, nonmetro households demonstrate comparable risks of housing quality hardship compared to metro areas after accounting for income-to-poverty ratios and all other covariates. Similarities in the prevalence of housing quality underscore the importance of renewed investment in affordable housing construction and rehabilitation in both metro and nonmetro communities (Brooks 2022). The findings also indicate that demographic and household characteristics explain the significant bivariate differences in housing hardship by metro status.

Differences in the prevalences of food hardship and experiencing any form of hardship between metro and nonmetro households disappear when adding household-level income-to-poverty ratios to the covariates. The sensitivity of results to an income measure points toward the central role of household income as an explanation for hardship disparities. As noted by Sen (1999), income offers the ability to purchase basic needs. Nonmetro areas have long exhibited higher levels of working poverty compared to metro settings (Thiede et al. 2018). In part, nonmetro jobs are more likely to be temporary and feature irregular schedules (Slack and Monnat 2024; Thiede et al. 2018). Nonmetro areas also have lower rates of unionization, suggesting that they may not benefit from higher wages associated with collective bargaining (Mettler and Brown 2022).

Nonmetro households’ elevated disability rates relative to metro households explain large shares of the hardship disparities. The findings align with previous research identifying disability status as an axis of social disadvantage (Heflin 2017). People with disabilities and their caregivers are less likely to be employed and have the financial resources necessary to purchase basic needs (Heflin et al. 2012).

### 5.1 | Theoretical Implications

Sen (1999) depicted material hardship as an indicator of social marginalization and exclusion, suggesting that people cannot enter community life without first meeting their most basic needs. This paper’s bivariate models suggest that nonmetro households may face greater risks of experiencing marginalization associated with food and housing hardships than those in metro communities. Nonmetro households’ elevated prevalence of also reporting any form of material hardship suggests the challenge is multifaceted. Continued bivariate disparities in hardship categories across metro and nonmetro areas could widen the existing socioeconomic, cultural, and political cleavages that separate them (Cramer 2016; Wuthnow 2018).

Although the results cannot prove rural dependency theory’s depiction of metro communities exploiting their nonmetro counterparts, they provide clues regarding the sources of nonmetro disadvantages in material hardship. The presence of a person with a disability in a household and household income-to-poverty ratios explains significant proportions of the metro–nonmetro gaps in material hardship. These factors highlight nonmetro households’ reliance on precarious, physically demanding jobs in industries, such as agriculture and coalmining, long upheld as examples of nonmetro exploitation (Lovejoy and Krannich 1982; Mueller 2020; Slack and Monnat 2024). The results suggest that rural dependency theory may operate on material hardship through income and disability.

Future research is needed to better understand hardship disparities in metro and nonmetro households. For instance, Black and Hispanic identifying households living in nonmetro areas face higher poverty rates than their metro peers (Farrigan 2023), but little work examines whether the disparities extend to material hardship. In addition, research should consider the long-term implications of COVID-19 on material hardship among nonmetro residents. During the pandemic, some metro residents moved to nonmetro areas rich with natural amenities, raising housing costs for longtime residents (Montañez 2024). The resulting gentrification processes might increase bill-paying hardships among some nonmetro households (Brooks 2022).

### 5.2 | Policy Implications

Policymakers can take targeted action to ease material hardship in both metro and nonmetro areas. Because of the important role of the income-to-poverty ratio in explaining material hardship disparities, policymakers should consider ways to increase household incomes. For instance, bolstering the size of monthly Supplemental Security Income payments for low-income people with disabilities could help a population at high risk of material hardship (Deshpande et al. 2021).^4^ Investments in SSI would especially benefit nonmetro households, whose results suggest are more likely to report a member with a disability than their metro counterparts. In addition, re-expanding the Child Tax Credit (CTC) could support nonmetro areas; it saved the average farm household approximately $2000 per year in 2021 (McDonald and Durst 2024). As noted by Lobao (2004, 2), such “aspatial government policy may have important spatial outcomes.” Given the association between income and material hardship, permanently expanding the CTC and bolstering SSI payments may reduce the risk of material hardship among nonmetro residents.

Increasing federal appropriations for rural affordable housing preservation and construction offer a place-based approach to easing hardship. Increased investment in the US Department of Agriculture’s Section 515 Rural Rental Housing Loan Program would bolster the government’s capacity to finance the construction and preservation of affordable rental units for nonmetro residents (Jones and McCarty 2022). Allocating additional resources to USDA’s housing programs could also help homeowners afford repairs and avoid housing quality hardship (Jones and McCarty 2022).

### 5.3 | Limitations

Some respondents are dropped because of missing metro status information, which may impact national representativeness. Changing definitions of metro and nonmetro areas make it difficult to compare variation in material hardship across multiple decades (Lichter and Brown 2011). In addition, the SIPP’s binary definition of metro status could mask variability in the wellbeing of nonmetro households in different settings (Brooks 2022; Montañez 2024). Meanwhile, respondents may feel ashamed to report difficulties meeting basic needs, biasing their outcomes (Karpman et al. 2018). It is unknown whether such social desirability biases would differentially influence the responses of metro and nonmetro residents.

The research provided here is observational and should not be interpreted as causal. Key covariates, such as disability and household income-to-poverty ratio, are likely endogenous to material hardship. Although disability status may predict material hardship (Heflin 2017), individuals also tend to report disability at higher rates amid poor economic conditions (O’Brien 2013). Likewise, even as low incomes make it more difficult to access basic needs, material hardship itself may undermine health and the ability to earn money (Coleman-Jensen and Steffen 2017).

## 6 | Conclusion

In bivariate models, nonmetro households demonstrate greater risks of experiencing food, housing quality, or any form of hardship between 2013 and 2021 compared to living in a metro setting. The disproportionate number of nonmetro households who struggle to provide for themselves underscores widespread social marginalization of nonmetro communities. After accounting for covariates and household income-to-poverty ratios, nonmetro households’ disadvantages relative to metro settings disappear, and the former demonstrate a lower prevalence of bill-paying hardship compared to the latter. A decomposition highlights the role of income and disability status as factors explaining bivariate hardship disparities. Policymakers should take steps to reduce material hardship in nonmetro areas by boosting incomes and improving housing quality for nonmetro households.

## Supplementary Material

Supplementary Material

Additional supporting information can be found online in the Supporting Information section. **Data S1:** ruso70028-sup-0001-supinfo.docx.

## Figures and Tables

**Table 1.** Weighted descriptive statistics by metropolitan status using data from 2014, 2018, and 2022 Waves of the Survey of Income and Program Participation.

	Complete Sample (N=69,375)		Metropolitan (N= 56,621)		Nonmetropolitan (N=13,114)	
	Percentage/Mean	SE	Percentage/Mean	SE	Percentage/Mean	SE
*Metropolitan status*						
Metropolitan	85.2%		100%		0%	
Nonmetropolitan	14.8%		0.0%		100.0%	
*Householder characteristics*						
Age	51.93	0.04	51.55	0.05	54.13***	0.21
Women	50.8%		50.5%		52.4%***	
Race and ethnicity						
Non-Hispanic white	66.1%		63.4%		81.9%***	
Non-Hispanic Black	12.6%		13.3%		8.4%***	
Hispanic	14.1%		15.4%		6.1%***	
Asian	5.0%		5.8%		0.6%***	
Other	2.2%		2.1%		3.0%***	
Born in the US	83.4%		81.0%		95.4%***	
Education attainment						
High school degree or less	35.7%		33.5%		47.8%***	
Some college	28.8%		28.5%		30.8%***	
Bachelor’s degree	21.7%		23.2%		13.5%***	
More than a bachelor’s degree	13.8%		14.8%		7.9%***	
Marital Status						
Never married	23.2%		24.0%		18.4%***	
Married	48.3%		48.3%		48.2%	
Divorced, separated, or widowed	28.5%		27.7%		33.4%***	
Householder employment status						
Employed	61.8%		63.1%		54.4%***	
Unemployed	2.4%		2.4%		2.2%	
Not in labor force	35.8%		34.5%		43.4%***	
*Household characteristics*						
Tenure						
Owned	61.7%		60.5%		68.8%***	
Renting	35.9%		37.5%		26.7%***	
Occupied without payment of rent	2.4%		2.1%		4.5%***	
Senior 65 years or older present	29.2%		28.5%		33.3%***	
Child present	28.2%		28.7%		25.6%***	
Person with a disability present	37.4%		39.8%		46.5%***	
Number of persons in the household	2.411	0.006	2.429	0.010	2.304***	0.017
Household income-to-poverty ratio						
Less than 1.00	13.9%		13.4%		16.4%***	
1.00–1.99	17.0%		16.1%		22.4%***	
2.00–2.99	15.8%		15.4%		18.3%***	
3.00–3.99	12.9%		12.8%		13.6%*	
4.00 or more	40.4%		42.4%		29.3%***	
*Region*	13.9%		13.4%		16.4%	
Northeast	15.0%		15.9%		10.3%	
Midwest	23.1%		20.8%		35.9%**	
South	38.4%		37.8%		41.9%	
West	23.5%		25.5%		11.9%	
*Reference year*						
2013	32.3%		32.1%		32.9%	
2017	33.3%		33.3%		33.5%	
2021	34.4%		34.6%		33.6%	

Notes: Two-sided t-test results of mean differences between metropolitan and nonmetropolitan shown.

* p<.05

** p<.01

*** p<.001

Source: Survey of Income and Program Participation’s Waves 2014, 2018, and 2022.

**Table 2.** Prevalence of material hardship components by metropolitan status using data from the 2014, 2018, and 2022 Waves of the Survey of Income and Program Participation.

	Complete Sample (N=69,735)	Metropolitan (N= 56,621)	Nonmetropolitan (N=13,114)
Metropolitan status			
Metropolitan	85.2%	100%	0%
Nonmetropolitan	14.8%	0%	100%
Food hardships			
Ate less than should have because of cost	6.9%	6.6%	8.1%***
Could not afford healthy meals	11.9%	11.4%	14.3%***
Cut size of meals or skipped meals because of cost	6.9%	6.7%	8.3%***
Hungry but did not eat because of cost	4.6%	4.5%	5.5%***
Purchased food did not last	12.7%	12.3%	15.1%***
**Food hardship (presence of at least two indicators)**	**11.2%**	**10.8%**	**13.4%** ***
Bill-paying hardships			
Unable to pay rent or mortgage	5.9%	6.0%	5.1%***
Unable to pay utilities	8.0%	8.0%	7.9%
**Bill-paying hardship (presence of any indicator)**	**10.1%**	**10.1%**	**10.1%**
Housing quality hardships			
Are there cracks in the ceiling or walls?	7.1%	6.7%	9.4%***
Are there holes in the floor?	1.6%	1.5%	2.4%***
Is there a problem with pests?	10.4%	10.3%	10.7%
Are there plumbing problems?	6.4%	6.4%	6.0%
**Housing quality hardship (presence of any indicator)**	**17.5%**	**17.3%**	**18.7%** **
**Any form of hardship (presence of at least one of the three hardships)**	**28.1%**	**27.8%**	**29.8%** ***

Notes: Two-sided test results of mean differences between metropolitan and nonmetropolitan shown.

** p<.01

*** p<.001

Source: Survey of Income and Program Participation Waves 2014, 2018, and 2022

**Table 3.** Linear probability regression estimates of the association between metropolitan status and food hardship.

	Model 1 (Base)		Model 2 (Base + controls)		Model 3 (Full)	
	b	se	b	se	b	se
Metropolitan status						
Metropolitan	(Reference)					
Nonmetropolitan	0.026***	0.006	0.014**	0.005	0.003	0.004
Household income-to-poverty ratio						
Less than 100%					(Reference)	
100%−199%					−0.020**	0.007
200%−299%					−0.087***	0.006
300%−399%					−0.119***	0.007
More than 400%					−0.144***	0.007
All household and householder covariates except household income-to-poverty ratio			✓		✓	
Observations	69,735		69,735		69,735	

Source: Survey of Income and Program Participation Waves 2014, 2018, and 2022

Notes: Coefficients and standard errors shown.

* p<0.05

** p<0.01

*** p<0.001

Models 2 and 3 account for householder characteristics (age, age-squared, gender, race and ethnicity, nativity, education attainment, marital status, and employment status), household characteristics (housing tenure, presence of a person with a disability, presence of a person 65 years or older, presence of a child, and number of persons present), region, and reference year. (See Supplementary Table 5 for full output.)

**Table 4.** Linear probability regression estimates of the association between metropolitan status and bill-paying hardship.

	Model 1 (Base)		Model 2 (Base + controls)		Model 3 (Full)	
	b	se	b	se	b	se
Metropolitan status						
Metropolitan	(Reference)					
Nonmetropolitan	0.000	0.004	−0.002	0.004	−0.009*	0.004
Household income-to-poverty ratio						
Less than 100%					(Reference)	
100%−199%					−0.020***	0.005
200%−299%					−0.057***	0.005
300%−399%					−0.076***	0.006
More than 400%					−0.102***	0.006
All household and householder covariates except household income-to-poverty ratio			✓		✓	
Observations	69,735		69,735		69,735	

Source: Survey of Income and Program Participation Waves 2014, 2018, and 2022

Notes: Coefficients and standard errors shown.

* p<0.05

** p<0.01

*** p<0.001

Models 2 and 3 account for householder characteristics (age, age-squared, gender, race and ethnicity, nativity, education attainment, marital status, and employment status), household characteristics (housing tenure, presence of a person with a disability, presence of a person 65 years or older, presence of a child, and number of persons present), region, and reference year. (See Supplementary Table 6 for full output.)

**Table 5.** Linear probability regression estimates of the association between metropolitan status and housing hardship.

	Model 1 (Base)		Model 2 (Base + controls)		Model 3 (Full)	
	b	se	b	se	b	se
Metropolitan status						
Metropolitan	(Reference)					
Nonmetropolitan	0.014*	0.007	0.009	0.005	0.004	0.005
Household income-to-poverty ratio						
Less than 100%					(Reference)	
100%−199%					−0.017*	0.007
200%−299%					−0.038***	0.006
300%−399%					−0.052***	0.008
More than 400%					−0.073***	0.007
All household and householder covariates except household income-to-poverty ratio			✓		✓	
Observations	69,735		69,735		69,735	

Source: Survey of Income and Program Participation Waves 2014, 2018, and 2022

Notes: Coefficients and standard errors shown.

* p<0.05

** p<0.01

*** p<0.001

Models 2 and 3 account for householder characteristics (age, age-squared, gender, race and ethnicity, nativity, education attainment, marital status, and employment status), household characteristics (housing tenure, presence of a person with a disability, presence of a person 65 years or older, presence of a child, and number of persons present), region, and reference year. (See Supplementary Table 7 for full output.)

**Table 6.** Linear probability regression estimates of the association between metropolitan status and experiencing any form of hardship.

	Model 1 (Base)		Model 2 (Base + controls)		Model 3 (Full)	
	b	se	b	se	b	se
Metropolitan status						
Metropolitan	(Reference)					
Nonmetropolitan	0.020*	0.008	0.012*	0.006	−0.001	0.005
Income-to-poverty ratio						
Less than 100%					(Reference)	
100%−199%					−0.028***	0.008
200%−299%					−0.088***	0.006
300%−399%					−0.129***	0.01
More than 400%					−0.174***	0.009
All household and householder covariates except household income-to-poverty ratio			✓		✓	
Observations	69,735		69,735		69,735	

Source: Survey of Income and Program Participation Waves 2014, 2018, and 2022

Notes: Coefficients and standard errors shown.

* p<0.05

** p<0.01

*** p<0.001

Models 2 and 3 account for householder characteristics (age, age-squared, gender, race and ethnicity, nativity, education attainment, marital status, and employment status), household characteristics (housing tenure, presence of a person with a disability, presence of a person 65 years or older, presence of a child, and number of persons present), region, and reference year. (See Supplementary Table 8 for full output.)

**Table 7.** Blinder-Oaxaca decompositions of differences in the prevalence of material hardships across metropolitan and nonmetropolitan households.

	Food		Bill-paying		Housing quality		Any hardship	
	b	se	b	se	b	se	b	se
Metropolitan status								
Metropolitan	0.108***	0.003	0.101***	0.003	0.173***	0.005	0.278***	0.005
Nonmetropolitan	0.134***	0.006	0.101***	0.005	0.187***	0.007	0.298***	0.009
*Difference*	−0.026***	0.006	0.000	0.005	−0.014*	0.007	−0.020*	0.008
*Explained by compositional effects*	−0.022***	0.003	−0.009**	0.003	−0.010*	0.004	−0.021***	0.005
Householder characteristics								
Age	0.005***	0.001	0.003***	0.001	0.004***	0.001	0.008***	0.001
Gender	−0.000*	0.000	−0.000*	0.000	0.000	0.000	−0.000*	0.000
Race and ethnicity	0.001	0.001	0.005***	0.001	0.004*	0.002	0.006***	0.002
Born in the U.S.	−0.001	0.001	−0.002*	0.001	−0.003*	0.001	−0.002**	0.001
Education attainment	−0.007***	0.001	−0.005***	0.001	−0.002*	0.001	−0.008***	0.001
Marital status	−0.001*	0.000	−0.001*	0.000	0.000	0.001	−0.001	0.001
Employment status	−0.002***	0.000	0.001*	0.000	0.000	0.000	−0.001*	0.000
Household characteristics								
Housing tenure	0.006***	0.001	0.004***	0.001	0.002*	0.001	0.007***	0.001
Person with a disability present	−0.010***	0.001	−0.008***	0.001	−0.012***	0.001	−0.017***	0.002
Person 65 years or older present	0.001***	0.000	0.001***	0.000	0.001*	0.000	0.001***	0.000
Child present	0.000	0.000	0.001**	0.000	−0.001*	0.000	0.000	0.000
Number of persons present	0.000	0.000	0.001*	0.000	0.001*	0.001	0.001*	0.000
Household income-to-poverty ratio	−0.014***	0.002	−0.010***	0.001	−0.007***	0.001	−0.017***	0.002
Region	−0.001	0.001	0.001	0.001	0.003*	0.001	0.003*	0.001
Reference year	0.000	0.000	0.000	0.000	0.000	0.000	0.000	0.000
*Unexplained*	−0.003	0.004	0.009*	0.004	−0.004	0.006	0.001	0.006
Observations	69,735		69,735		69,735		69,735	

Notes: Coefficients and standard errors shown.

* p<.05

** p<.01

*** p<.001

Source: Survey of Income and Program Participation Waves 2014, 2018, and 2022. (See Supplementary Table 9 for full output.)

## Data Availability

The data that support the findings of this study are openly available at https://www.census.gov/programs-surveys/sipp/data/datasets.html.

## Footnotes

1 The Census Bureau’s binary metropolitan status indicator classifies counties with municipalities of 50,000 or more residents—and the surrounding counties closely linked via economic ties—as metropolitan counties. All other counties are identified as nonmetropolitan (Office of Management and Budget 2013). The inclusion of metropolitan status as a measure of rurality reflects the practices of other researchers and improves the comparability of findings across studies (Mueller et al. 2022; Pacas and Rothwell 2020). To improve clarity, I follow the Office of Management and Budget definition and use *nonmetropolitan* to describe households living outside of metropolitan counties. *Rural* is used when discussing the broader field of rural sociology or the names of specific theoretical approaches.

2 I drop Alaska, Connecticut, Maryland, Massachusetts, Vermont, and Wyoming from the sample completely because all their residents are missing data on metropolitan status. The states constitute less than 6% of the weighted sample size.

3 Social scientists often use principal component factor analysis to identify latent constructs and reduce the number of outcome variables (Dunteman 1989; Iceland and Cho 2025). Using this study’s data, principal component factor analysis groups hardship variables into food, bill-paying, and housing quality categories. This indicates that the hardship categories suggested by the literature match the data (Heflin 2017; Iceland and Sakamoto 2022).

4 The average SSI recipient received $675 per month in 2023 (Social Security Administration 2024).
