# Supplementary material for: Material Hardship Across Metropolitan and Nonmetropolitan Counties in the United States: 2013–2021
Source: Rural Sociol. Author manuscript; Available in PMC 2026 Sep 18. (PMC13585042; doi:10.1111/ruso.70028)
Supplement: Supplementary file 1 [file NIHMS2145885-supplement-Supplementary_Material.docx]

**Material Hardship Across Metropolitan and Nonmetropolitan Counties in the US:**

**2013-2021**

**Supplementary Material**

Contents

[Supplementary Figure 1. Predicted probabilities of material hardships and key coefficients from multivariate linear probability regression estimates inclusive of household income-to-poverty ratios.	4](#_Toc207280632)

[Supplementary Material Table 1. Material hardship questions from the Survey of Income and Program Participation 2014, 2018, and 2022 waves.	6](#_Toc207280633)

[Supplementary Material Table 2. Pearson’s R correlation values examining the association between material hardship types and household income-to-poverty ratios (N=69,735).	7](#_Toc207280634)

[Supplementary Table 3. Variance inflation factors (VIF) of covariates in fully specified regressions inclusive of household income-to-poverty ratios.	8](#_Toc207280635)

[Supplementary Table 4. Linear probability regression estimates of the association between metropolitan status and household earnings below the official poverty line.	10](#_Toc207280636)

[Supplementary Table 4 (continued). Linear probability regression estimates of the association between metropolitan status and household earnings below the official poverty line.	11](#_Toc207280637)

[Supplementary Table 5. Linear probability regression estimates of the association between metropolitan status and food hardship (Table 3 with all covariate coefficients).	12](#_Toc207280638)

[Supplementary Table 6. Linear probability regression estimates of the association between metropolitan status and bill-paying hardship (Table 4 with all covariate coefficients).	14](#_Toc207280639)

[Supplementary Table 7. Linear probability regression estimates of the association between metropolitan status and housing hardship (Table 5 with all covariate coefficients).	16](#_Toc207280640)

[Supplementary Table 8. Linear probability regression estimates of the association between metropolitan status and any form of hardship (Table 6 with all covariate coefficients).	18](#_Toc207280641)

[Supplementary Table 9. Blinder-Oaxaca decompositions of differences in the prevalence of material hardships across metropolitan and nonmetropolitan households. (Table 7 with all covariate coefficients shown.)	20](#_Toc207280642)

[Supplementary Table 10. Linear probability regression estimates of the association between metropolitan status and food hardship using multiple imputation.	21](#_Toc207280643)

[Supplementary Table 11. Linear probability regression estimates of the association between metropolitan status and bill-paying hardship using multiple imputation.	23](#_Toc207280644)

[Supplementary Table 12. Linear probability regression estimates of the association between metropolitan status and housing hardship using multiple imputation.	25](#_Toc207280645)

[Supplementary Table 13. Linear probability regression estimates of the association between metropolitan status and any form of hardship using multiple imputation.	27](#_Toc207280646)

[Supplementary Table 14. Blinder-Oaxaca decompositions of differences in the prevalence of material hardships across metropolitan and nonmetropolitan households using multiple imputation.	29](#_Toc207280647)

[Supplementary Table 15. Multivariate linear probability regression estimates of the association between metropolitan status and food hardship using an indicator to identify unreported metropolitan status.	30](#_Toc207280648)

[Supplementary Table 16. Multivariate linear probability regression estimates of the association between metropolitan status and bill-paying hardship using an indicator to identify unreported metropolitan status.	32](#_Toc207280649)

[Supplementary Table 17. Multivariate linear probability regression estimates of the association between metropolitan status and housing quality hardship using an indicator to identify unreported metropolitan status.	34](#_Toc207280650)

[Supplementary Table 18. Multivariate linear probability regression estimates of the association between metropolitan status and any form of hardship using an indicator to identify unreported metropolitan status.	36](#_Toc207280651)

[Supplementary Table 19. Linear probability regression estimates of the association between metropolitan status and food hardship excluding data about 2021.	38](#_Toc207280652)

[Supplementary Table 20. Linear probability regression estimates of the association between metropolitan status and bill-paying hardship excluding data about 2021.	40](#_Toc207280653)

[Supplementary Table 21. Linear probability regression estimates of the association between metropolitan status and housing hardship excluding data about 2021.	42](#_Toc207280654)

[Supplementary Table 22. Linear probability regression estimates of the association between metropolitan status and any form of hardship excluding data about 2021.	44](#_Toc207280655)

[Supplementary Table 23. Blinder-Oaxaca decompositions of differences in the prevalence of material hardships across metropolitan and nonmetropolitan households excluding data about 2021.	46](#_Toc207280656)

[Supplementary Table 24. Multivariate linear probability regression estimates of the association between metropolitan status and food hardships using a composite food hardship scale.	47](#_Toc207280657)

[Supplementary Table 25. Multivariate linear probability regression estimates of the association between metropolitan status and housing quality hardship using a composite housing quality hardship scale.	49](#_Toc207280658)

[Supplementary Table 26. Multivariate linear probability regression estimates of the association between metropolitan status and all forms of hardship using a composite hardship scale.	51](#_Toc207280659)

[Supplementary Table 27. Blinder-Oaxaca decompositions of differences in the prevalence of material hardships across metropolitan and nonmetropolitan households using composite hardship scales.	53](#_Toc207280660)

[Supplementary Table 28. Multivariate logistic regression estimates of the association between metropolitan status and food hardship.	54](#_Toc207280661)

[Supplementary Table 29. Multivariate logistic regression estimates of the association between metropolitan status and bill-paying hardship.	56](#_Toc207280662)

[Supplementary Table 30. Multivariate logistic regression estimates of the association between metropolitan status and housing hardship.	58](#_Toc207280663)

[Supplementary Table 31. Multivariate logistic regression estimates of the association between metropolitan status and any form of hardship.	60](#_Toc207280664)

[Supplementary Table 32. Blinder-Oaxaca decompositions of differences in the prevalence of material hardships across metropolitan and nonmetropolitan households using logistic regression.	62](#_Toc207280665)

[Supplementary Table 33. Fairlie decompositions of differences in the prevalence of material hardships across metropolitan and nonmetropolitan households.	63](#_Toc207280666)

# Supplementary Figure 1. Predicted probabilities of material hardships and key coefficients from multivariate linear probability regression estimates inclusive of household income-to-poverty ratios.

Panel A.

Panel B.

*Supplementary Figure 1 (continued). Predicted probabilities of material hardships and key coefficients from multivariate linear probability regression estimates inclusive of household income-to-poverty ratios.*

Panel C.

Panel D.

Note: Householder character.= Householder characteristics

Household character.=Household characteristics

Source: Survey of Income and Program Participation Waves 2014, 2018, and 2022

# Supplementary Material Table 1. Material hardship questions from the Survey of Income and Program Participation 2014, 2018, and 2022 waves.

| Hardship | Question | Answer | Code |
| --- | --- | --- | --- |
| **Food hardship**  (Food hardship=1 if the total score in the far right column is two or higher.) | These next questions are about whether [you/or reference person] could afford the food needed during past calendar year. I'm going to read you some statements that people have made about their food situations.  The first statement is, "The food that we bought just didn't last, and we didn't have money to get more." Was that often, sometimes, or never true for [your/or reference person’s household] in [reference year]? | Often, sometimes,  or never true | Often or sometimes true=1  Never true=0 |
|  | “[We] couldn't afford to eat balanced meals." Was that often, sometimes, or never true for [you/ your or reference person’s household] in [reference year]? | Often, sometimes, or never true | Often or sometimes true=1  Never true=0 |
|  | In [reference year], did [you or other adults] ever cut the size of meals or skip meals because there wasn't enough money for food? | Yes or no | Yes=1  No=0 |
|  | In [reference year], did [you or other adult] ever eat less than [he/she] felt you should because there wasn't enough money for food? | Yes or no | Yes=1  No=0 |
| **Bill-paying hardship**  (Bill-paying hardship=1 if the total score in the far right column is one or higher.) | Next are questions about difficulties people sometimes have in meeting their essential household expenses.  During [reference year], was there ANY time when [you / your household] did not pay the full amount of the rent or mortgage? | Yes or no | Yes=1  No=0 |
|  | During [reference year], was there ANY time when [you / your household] did not pay the full amount of the gas, oil, or electricity bill? | Yes or no | Yes=1  No=0 |
| **Housing hardship**  (Housing hardship occurs if the total score in the far right is 1 or higher.) | I have some questions about the place where [you or reference person] [was/were] living in [reference year]. Were the following conditions present:  …holes in the walls or ceiling, or cracks wider than the edge of a dime? | Yes or no | Yes=1  No=0 |
|  | …holes in the floor big enough to catch your foot on? | Yes or no | Yes=1  No=0 |
|  | … problems with pests such as rats, mice, roaches, or other insects? | Yes or no | Yes=1  No=0 |
|  | … a toilet, hot water heater, or other plumbing that didn’t work? | Yes or no | Yes=1  No=0 |
| **Any form of hardship**  (Any form of hardship=1 if any of the above bolded hardship categories =1.) | NA | NA | NA |

Source: Survey of Income and Program Participation Waves 2014, 2018, and 2022.

# Supplementary Material Table 2. Pearson’s R correlation values examining the association between material hardship types and household income-to-poverty ratios (N=69,735).

|  | Household income-to-poverty ratio | | | | |
| --- | --- | --- | --- | --- | --- |
|  | Less than 100% | 100%-199% | 200%-299% | 300%-399% | More than  400% |
| Food hardship | 0.193*** | 0.145*** | 0.014*** | -0.044*** | -0.227*** |
| Bill-paying hardship | 0.141*** | 0.098*** | 0.018*** | -0.023*** | -0.172*** |
| Housing quality  hardship | 0.098*** | 0.063*** | 0.014*** | -0.014*** | -0.119*** |
| Any form of hardship | 0.176*** | 0.126*** | 0.028*** | -0.030*** | -0.220*** |

Notes: All correlations are statistically significant at p<0.001. Results suggest limited-to-moderate correlations between material hardship and income-to poverty ratios (Chen and Popovich 2002). The threshold for moderate correlation is 0.2

Source: Survey of Income and Program Participation Waves 2014, 2018, and 2022

| Supplementary Table 3. Variance inflation factors (VIF) of covariates in fully specified regressions inclusive of household income-to-poverty ratios. | | |
| --- | --- | --- |
| Variable | VIF | 1/VIF |
| Metropolitan status |  |  |
| Metropolitan | (Reference) | |
| Nonmetropolitan | 1.09 | 0.919 |
| Householder characteristics |  |  |
| Age | 44.88 | 0.022 |
| Age^2^ | 51.22 | 0.020 |
| Gender |  |  |
| Male | (Reference) | |
| Female | 1.08 | 0.927 |
| Race and ethnicity |  |  |
| Non-Hispanic white | (Reference) | |
| Non-Hispanic Black | 1.17 | 0.852 |
| Hispanic | 1.48 | 0.674 |
| Asian | 1.36 | 0.735 |
| Other | 1.02 | 0.976 |
| Born in the U.S. | 1.59 | 0.628 |
| Education attainment |  |  |
| High school degree or less | (Reference) | |
| Some college | 1.37 | 0.732 |
| Bachelor's degree | 1.50 | 0.668 |
| More than a bachelor's degree | 1.43 | 0.701 |
| Marital status |  |  |
| Never married | (Reference) | |
| Married | 2.64 | 0.378 |
| Divorced, separated, or widowed | 2.26 | 0.442 |
| Employment status |  |  |
| Employed | (Reference) | |
| Unemployed | 1.06 | 0.946 |
| Not in labor force | 1.80 | 0.555 |
| Household characteristics |  |  |
| Housing tenure |  |  |
| Owner | (Reference) | |
| Renter | 1.45 | 0.688 |
| Occupy without payment of rent | 1.06 | 0.941 |
| Person with a disability present | 1.27 | 0.790 |
| Person 65 years or older present | 2.97 | 0.336 |
| Child present | 2.21 | 0.452 |
| Number of persons present | 2.46 | 0.407 |
| Income-to-poverty ratio |  |  |
| Less than 100% | (Reference) | |
| 100%-199% | 1.95 | 0.512 |
| 200%-299% | 2.02 | 0.494 |
| 300%-399% | 1.96 | 0.509 |
| More than 400% | 3.51 | 0.285 |
| Region |  |  |
| Northeast | (Reference) | |
| Midwest | 2.00 | 0.499 |
| South | 2.25 | 0.444 |
| West | 2.00 | 0.499 |

| *Supplementary Table 3 (continued). Variance inflation factors (VIF) for covariates in fully specified regressions inclusive of household income-to-poverty ratios.* | | |
| --- | --- | --- |
| Variable | VIF | 1/VIF |
| Reference year |  |  |
| 2013 | (Reference) | |
| 2017 | 1.38 | 0.727 |
| 2021 | 1.40 | 0.716 |
| Mean VIF | 4.74 |  |

Source: Survey of Income and Program Participation Waves 2014, 2018, and 2022

# Supplementary Table 4. Linear probability regression estimates of the association between metropolitan status and household earnings below the official poverty line.

|  | b | se |
| --- | --- | --- |
| Metropolitan status |  |  |
| Metropolitan | (Reference) | |
| Nonmetropolitan | 0.021*** | 0.005 |
| Householder characteristics |  |  |
| Age | (Reference) | |
| Age^2^ | -0.000*** | 0.000 |
| Gender |  |  |
| Men | (Reference) | |
| Women | 0.009*** | 0.002 |
| Race and ethnicity |  |  |
| Non-Hispanic white | (Reference) | |
| Non-Hispanic Black | 0.036*** | 0.006 |
| Hispanic | 0.014* | 0.006 |
| Asian | 0.012 | 0.007 |
| Other | 0.025** | 0.009 |
| Born in the U.S. | -0.052*** | 0.005 |
| Education attainment |  |  |
| High school degree or less | (Reference) | |
| Some college | -0.040*** | 0.004 |
| Bachelor's degree | -0.072*** | 0.004 |
| More than a bachelor's degree | -0.067*** | 0.005 |
| Marital status |  |  |
| Never married | (Reference) | |
| Married | -0.120*** | 0.004 |
| Divorced, separated, or widowed | -0.049*** | 0.005 |
| Employment status |  |  |
| Employed | (Reference) | |
| Unemployed | 0.291*** | 0.015 |
| Not in labor force | 0.219*** | 0.006 |
| Household characteristics |  |  |
| Housing tenure |  |  |
| Owner | (Reference) | |
| Renter | 0.072*** | 0.004 |
| Occupy without payment of rent | 0.152*** | 0.012 |
| Person with a disability present | 0.023*** | 0.004 |
| Person 65 years or older present | -0.078*** | 0.005 |
| Child present | 0.040*** | 0.005 |
| Number of persons present | -0.003 | 0.002 |

# Supplementary Table 4 (continued). Linear probability regression estimates of the association between metropolitan status and household earnings below the official poverty line.

|  | b | se |
| --- | --- | --- |
| Household characteristics |  |  |
| Material hardship indicators |  |  |
| Food hardship | 0.071*** | 0.006 |
| Bill-paying hardship | 0.039*** | 0.006 |
| Housing quality hardship | 0.014*** | 0.004 |
| Region |  |  |
| Northeast | (Reference) | |
| Midwest | -0.004 | 0.007 |
| South | 0.015* | 0.007 |
| West | -0.011 | 0.006 |
| Reference year |  |  |
| 2013 | (Reference) | |
| 2017 | -0.009*** | 0.003 |
| 2021 | -0.010* | 0.004 |
| Constant | 0.232*** | 0.019 |
| Observations | 69,735 |  |
| Note: Coefficients and standard errors shown. *p<.05 ** p<.01 *** p<.001  Even after controlling for material hardship conditions, nonmetropolitan residence is predictive of living below the official poverty line. There was no model in which nonmetropolitan residence predicted elevated risk of a material hardship outcome. The different results suggest that the relationship between nonmetropolitan residence and income-based poverty is unique from the association between nonmetropolitan residence and hardship.  Source: Survey of Income and Program Participation | | |

# Supplementary Table 5. Linear probability regression estimates of the association between metropolitan status and food hardship (Table 3 with all covariate coefficients).

|  | Model 1  (Base) | | Model 2  (Base + controls) | | Model 3  (Full) | |
| --- | --- | --- | --- | --- | --- | --- |
|  | b | se | b | se | b | se |
| Metropolitan status |  |  |  |  |  |  |
| Metropolitan | (Reference) | | | | | |
| Nonmetropolitan | 0.026*** | 0.006 | 0.014** | 0.005 | 0.003 | 0.004 |
| Householder characteristics |  |  |  |  |  |  |
| Age |  |  | 0.007*** | 0.000 | 0.008*** | 0.000 |
| Age^2^ |  |  | -0.000*** | 0.000 | -0.000*** | 0.000 |
| Gender |  |  |  |  |  |  |
| Men |  |  | (Reference) | | | |
| Women |  |  | 0.018*** | 0.002 | 0.015*** | 0.002 |
| Race and ethnicity |  |  |  |  |  |  |
| Non-Hispanic white |  |  | (Reference) | | | |
| Non-Hispanic Black |  |  | 0.035*** | 0.005 | 0.027*** | 0.006 |
| Hispanic |  |  | 0.021*** | 0.004 | 0.013** | 0.004 |
| Asian |  |  | -0.017** | 0.005 | -0.018** | 0.006 |
| Other |  |  | 0.042*** | 0.009 | 0.036*** | 0.009 |
| Born in the U.S. |  |  | -0.005 | 0.007 | 0.007 | 0.006 |
| Education attainment |  |  |  |  |  |  |
| High school degree or less |  |  | (Reference) | | | |
| Some college |  |  | -0.022*** | 0.004 | -0.011** | 0.004 |
| Bachelor's degree |  |  | -0.069*** | 0.004 | -0.042*** | 0.004 |
| More than a bachelor's degree |  |  | -0.073*** | 0.004 | -0.041*** | 0.004 |
| Marital status |  |  |  |  |  |  |
| Never married |  |  | (Reference) | | | |
| Married |  |  | -0.041*** | 0.004 | -0.014** | 0.004 |
| Divorced, separated, or widowed |  |  | 0.015* | 0.006 | 0.017** | 0.006 |
| Employment status |  |  |  |  |  |  |
| Employed |  |  | (Reference) | | | |
| Unemployed |  |  | 0.150*** | 0.018 | 0.110*** | 0.017 |
| Not in labor force |  |  | 0.055*** | 0.005 | 0.020*** | 0.004 |
| Household characteristics |  |  |  |  |  |  |
| Housing tenure |  |  |  |  |  |  |
| Owner |  |  | (Reference) | | | |
| Renter |  |  | 0.082*** | 0.004 | 0.060*** | 0.004 |
| Occupy without payment of rent |  |  | 0.041** | 0.014 | 0.012 | 0.013 |
| Person with a disability present |  |  | 0.099*** | 0.003 | 0.089*** | 0.003 |
| Person 65 years or older present |  |  | -0.034*** | 0.004 | -0.024*** | 0.004 |
| Child present |  |  | 0.018*** | 0.004 | 0.002 | 0.004 |
| Number of persons present |  |  | -0.002 | 0.002 | -0.003 | 0.002 |
| Income-to-poverty ratio |  |  |  |  |  |  |
| Less than 100% |  |  |  |  | (Reference) | |
| 100%-199% |  |  |  |  | -0.020** | 0.007 |
| 200%-299% |  |  |  |  | -0.087*** | 0.006 |
| 300%-399% |  |  |  |  | -0.119*** | 0.007 |
| More than 400% |  |  |  |  | -0.144*** | 0.007 |

*Supplementary Table 5 (continued). Linear probability regression estimates of the association between metropolitan status and food hardship (Table 3 with all covariate coefficients).*

|  | Model 1  (Base) | | Model 2  (Base + controls) | | Model 3  (Full) | |
| --- | --- | --- | --- | --- | --- | --- |
|  | b | se | b | se | b | se |
| Region |  |  |  |  |  |  |
| Northeast |  |  | (Reference) | | | |
| Midwest |  |  | 0.009 | 0.005 | 0.007 | 0.004 |
| South |  |  | 0.016*** | 0.004 | 0.010* | 0.004 |
| West |  |  | 0.001 | 0.005 | 0.002 | 0.005 |
| Year |  |  |  |  |  |  |
| 2013 |  |  | (Reference) | | | |
| 2017 |  |  | -0.025*** | 0.003 | -0.020*** | 0.003 |
| 2021 |  |  | -0.034*** | 0.003 | -0.027*** | 0.003 |
| Constant | 0.108*** | 0.003 | -0.055*** | 0.014 | 0.006 | 0.013 |
| Observations | 69,735 |  | 69,735 |  | 69,735 |  |
| Note: Coefficients and standard errors shown. *p<.05 ** p<.01 *** p<.001  Source: Survey of Income and Program Participation Waves 2014, 2018, and 2022 | | | | | | |

# Supplementary Table 6. Linear probability regression estimates of the association between metropolitan status and bill-paying hardship (Table 4 with all covariate coefficients).

|  | | Model 1  (Base) | | | Model 2  (Base + controls) | | | Model 3  (Full) | | |
| --- | --- | --- | --- | --- | --- | --- | --- | --- | --- | --- |
|  | b | | se | b | | se | b | | se |  |
| Metropolitan status |  | |  |  | |  |  | |  |  |
| Metropolitan | (Reference) | | | | | | | | |  |
| Nonmetropolitan | 0.000 | | 0.004 | -0.002 | | 0.004 | -0.009* | | 0.004 |  |
| Householder characteristics |  | |  |  | |  |  | |  |  |
| Age |  | |  | 0.007*** | | 0.001 | 0.007*** | | 0.001 |  |
| Age^2^ |  | |  | -0.000*** | | 0.000 | -0.000*** | | 0.000 |  |
| Gender |  | |  |  | |  |  | |  |  |
| Men |  | |  | (Reference) | | | | | |  |
| Women |  | |  | 0.016*** | | 0.003 | 0.014*** | | 0.003 |  |
| Race and ethnicity |  | |  |  | |  |  | |  |  |
| Non-Hispanic white |  | |  | (Reference) | | | | | |  |
| Non-Hispanic Black |  | |  | 0.071*** | | 0.007 | 0.065*** | | 0.007 |  |
| Hispanic |  | |  | 0.037*** | | 0.004 | 0.032*** | | 0.004 |  |
| Asian |  | |  | -0.013* | | 0.005 | -0.014* | | 0.005 |  |
| Other |  | |  | 0.046*** | | 0.012 | 0.043*** | | 0.011 |  |
| Born in the U.S. |  | |  | 0.004 | | 0.005 | 0.012* | | 0.005 |  |
| Education attainment |  | |  |  | |  |  | |  |  |
| High school degree or less |  | |  | (Reference) | | | | | |  |
| Some college |  | |  | -0.001 | | 0.004 | 0.006+ | | 0.004 |  |
| Bachelor's degree |  | |  | -0.045*** | | 0.004 | -0.026*** | | 0.004 |  |
| More than a bachelor's degree |  | |  | -0.051*** | | 0.003 | -0.028*** | | 0.004 |  |
| Marital status |  | |  |  | |  |  | |  |  |
| Never married |  | |  | (Reference) | | | | | |  |
| Married |  | |  | -0.031*** | | 0.005 | -0.012** | | 0.004 |  |
| Divorced, separated, or widowed |  | |  | 0.016** | | 0.005 | 0.018*** | | 0.005 |  |
| Employment status |  | |  |  | |  |  | |  |  |
| Employed |  | |  | (Reference) | | | | | |  |
| Unemployed |  | |  | 0.134*** | | 0.012 | 0.106*** | | 0.012 |  |
| Not in labor force |  | |  | 0.017*** | | 0.003 | -0.007* | | 0.003 |  |
| Household characteristics |  | |  |  | |  |  | |  |  |
| Housing tenure |  | |  |  | |  |  | |  |  |
| Owner |  | |  | (Reference) | | | | | |  |
| Renter |  | |  | 0.049*** | | 0.004 | 0.033*** | | 0.004 |  |
| Occupy without payment of rent |  | |  | 0.019 | | 0.012 | -0.001 | | 0.011 |  |
| Person with a disability present |  | |  | 0.078*** | | 0.003 | 0.071*** | | 0.003 |  |
| Person 65 years or older present |  | |  | -0.024*** | | 0.003 | -0.018*** | | 0.003 |  |
| Child present |  | |  | 0.039*** | | 0.004 | 0.028*** | | 0.004 |  |
| Number of persons present |  | |  | 0.005*** | | 0.002 | 0.005*** | | 0.001 |  |
| Household income-to-poverty ratio |  | |  |  | |  |  | |  |  |
| Less than 100% |  | |  |  | |  | (Reference) | | |  |
| 100%-199% |  | |  |  | |  | -0.020*** | | 0.005 |  |
| 200%-299% |  | |  |  | |  | -0.057*** | | 0.005 |  |
| 300%-399% |  | |  |  | |  | -0.076*** | | 0.006 |  |
| More than 400% |  | |  |  | |  | -0.102*** | | 0.006 |  |

*Supplementary Table 6 (continued). Linear probability regression estimates of the association between metropolitan status and bill-paying hardship (Table 4 with all covariate coefficients).*

|  | Model 1  (Base) | | Model 2  (Base + controls) | | Model 3  (Full) | |
| --- | --- | --- | --- | --- | --- | --- |
|  | b | se | b | se | b | se |
| Region |  |  |  |  |  |  |
| Northeast |  |  | (Reference) | | | |
| Midwest |  |  | -0.016*** | 0.003 | -0.017*** | 0.003 |
| South |  |  | -0.020*** | 0.003 | -0.024*** | 0.003 |
| West |  |  | -0.020*** | 0.003 | -0.019*** | 0.004 |
| Year |  |  |  |  |  |  |
| 2013 |  |  | (Reference) | | | |
| 2017 |  |  | -0.026*** | 0.004 | -0.023*** | 0.004 |
| 2021 |  |  | -0.037*** | 0.004 | -0.033*** | 0.004 |
| Constant | 0.101*** | 0.002 | -0.064*** | 0.015 | -0.022 | 0.014 |
| Observations | 69,735 |  | 69,735 |  | 69,735 |  |
| Note: Coefficients and standard errors shown. *p<.05 ** p<.01 *** p<.001  Source: Survey of Income and Program Participation Waves 2014, 2018, and 2022 | | | | | | |

# Supplementary Table 7. Linear probability regression estimates of the association between metropolitan status and housing hardship (Table 5 with all covariate coefficients).

|  | Model 1  (Base) | | Model 2  (Base + controls) | | Model 3  (Full) | |
| --- | --- | --- | --- | --- | --- | --- |
|  | b | se | b | se | b | se |
| Metropolitan status |  |  |  |  |  |  |
| Metropolitan | (Reference) | | | | | |
| Nonmetropolitan | 0.014* | 0.007 | 0.009+ | 0.005 | 0.004 | 0.005 |
| Householder characteristics |  |  |  |  |  |  |
| Age |  |  | 0.003*** | 0.001 | 0.003*** | 0.001 |
| Age^2^ |  |  | -0.000*** | 0.000 | -0.000*** | 0.000 |
| Gender |  |  |  |  |  |  |
| Men |  |  | (Reference) | | | |
| Women |  |  | 0.007* | 0.003 | 0.005 | 0.003 |
| Race and ethnicity |  |  |  |  |  |  |
| Non-Hispanic white |  |  | (Reference) | | | |
| Non-Hispanic Black |  |  | 0.021* | 0.008 | 0.017* | 0.008 |
| Hispanic |  |  | 0.032* | 0.012 | 0.028* | 0.012 |
| Asian |  |  | 0.018+ | 0.01 | 0.017+ | 0.009 |
| Other |  |  | 0.069*** | 0.014 | 0.067*** | 0.013 |
| Born in the U.S. |  |  | 0.014+ | 0.007 | 0.019** | 0.007 |
| Education attainment |  |  |  |  |  |  |
| High school degree or less |  |  | (Reference) | | | |
| Some college |  |  | -0.008+ | 0.004 | -0.002 | 0.004 |
| Bachelor's degree |  |  | -0.030*** | 0.007 | -0.017* | 0.007 |
| More than a bachelor's degree |  |  | -0.024** | 0.007 | -0.008 | 0.007 |
| Marital status |  |  |  |  |  |  |
| Never married |  |  | (Reference) | | | |
| Married |  |  | -0.057*** | 0.007 | -0.044*** | 0.007 |
| Divorced, separated, or widowed |  |  | 0.001 | 0.006 | 0.002 | 0.006 |
| Employment status |  |  |  |  |  |  |
| Employed |  |  | (Reference) | | | |
| Unemployed |  |  | 0.074*** | 0.012 | 0.055*** | 0.013 |
| Not in labor force |  |  | 0.023*** | 0.003 | 0.006* | 0.003 |
| Household characteristics |  |  |  |  |  |  |
| Housing tenure |  |  |  |  |  |  |
| Owner |  |  | (Reference) | | | |
| Renter |  |  | 0.041*** | 0.007 | 0.031*** | 0.008 |
| Occupy without payment of rent |  |  | 0.070*** | 0.012 | 0.057*** | 0.012 |
| Person with a disability present |  |  | 0.110*** | 0.004 | 0.105*** | 0.004 |
| Person 65 years or older present |  |  | -0.020** | 0.007 | -0.015* | 0.006 |
| Child present |  |  | -0.013* | 0.006 | -0.021** | 0.007 |
| Number of persons present |  |  | 0.011*** | 0.002 | 0.011*** | 0.002 |
| Income-to-poverty ratio |  |  |  |  |  |  |
| Less than 100% |  |  |  |  | (Reference) | |
| 100%-199% |  |  |  |  | -0.017* | 0.007 |
| 200%-299% |  |  |  |  | -0.038*** | 0.006 |
| 300%-399% |  |  |  |  | -0.052*** | 0.008 |
| More than 400% |  |  |  |  | -0.073*** | 0.007 |

*Supplementary Table 7 (continued). Linear probability regression estimates of the association between metropolitan status and housing hardship (Table 5 with all covariate coefficients).*

|  | Model 1  (Base) | | Model 2  (Base + controls) | | Model 3  (Full) | |
| --- | --- | --- | --- | --- | --- | --- |
|  | b | se | b | se | b | se |
| Region |  |  |  |  |  |  |
| Northeast |  |  | (Reference) | | | |
| Midwest |  |  | -0.025* | 0.011 | -0.026* | 0.011 |
| South |  |  | -0.008 | 0.014 | -0.011 | 0.014 |
| West |  |  | -0.012 | 0.012 | -0.012 | 0.012 |
| Year |  |  |  |  |  |  |
| 2013 |  |  | (Reference) | | | |
| 2017 |  |  | 0.008+ | 0.004 | 0.010* | 0.004 |
| 2021 |  |  | -0.005 | 0.005 | -0.002 | 0.005 |
| Constant | 0.173*** | 0.005 | 0.083*** | 0.016 | 0.113*** | 0.017 |
| Observations | 69,735 |  | 69,735 |  | 69,735 |  |
| Note: Coefficients and standard errors shown. *p<.05 ** p<.01 *** p<.001  Source: Survey of Income and Program Participation Waves 2014, 2018, and 2022 | | | | | | |

# Supplementary Table 8. Linear probability regression estimates of the association between metropolitan status and any form of hardship (Table 6 with all covariate coefficients).

|  | Model 1  (Base) | | Model 2  (Base + controls) | | Model 3  (Full) | |
| --- | --- | --- | --- | --- | --- | --- |
|  | b | se | b | se | b | se |
| Metropolitan status |  |  |  |  |  |  |
| Metropolitan | (Reference) | | | | | |
| Nonmetropolitan | 0.020* | 0.008 | 0.012* | 0.006 | -0.001 | 0.005 |
| Householder characteristics |  |  |  |  |  |  |
| Age |  |  | 0.009*** | 0.001 | 0.010*** | 0.001 |
| Age^2^ |  |  | -0.000*** | 0.000 | -0.000*** | 0.000 |
| Gender |  |  |  |  |  |  |
| Men |  |  | (Reference) | | | |
| Women |  |  | 0.025*** | 0.003 | 0.021*** | 0.004 |
| Race and ethnicity |  |  |  |  |  |  |
| Non-Hispanic white |  |  | (Reference) | | | |
| Non-Hispanic Black |  |  | 0.074*** | 0.009 | 0.064*** | 0.009 |
| Hispanic |  |  | 0.058*** | 0.009 | 0.048*** | 0.009 |
| Asian |  |  | -0.004 | 0.008 | -0.006 | 0.008 |
| Other |  |  | 0.104*** | 0.017 | 0.097*** | 0.016 |
| Born in the U.S. |  |  | 0.003 | 0.005 | 0.017** | 0.005 |
| Education attainment |  |  |  |  |  |  |
| High school degree or less |  |  | (Reference) | | | |
| Some college |  |  | -0.018** | 0.006 | -0.005 | 0.006 |
| Bachelor's degree |  |  | -0.083*** | 0.009 | -0.050*** | 0.008 |
| More than a bachelor's degree |  |  | -0.085*** | 0.008 | -0.044*** | 0.007 |
| Marital status |  |  |  |  |  |  |
| Never married |  |  | (Reference) | | | |
| Married |  |  | -0.082*** | 0.007 | -0.050*** | 0.007 |
| Divorced, separated, or widowed |  |  | 0.017* | 0.008 | 0.021** | 0.007 |
| Employment status |  |  |  |  |  |  |
| Employed |  |  | (Reference) | | | |
| Unemployed |  |  | 0.174*** | 0.017 | 0.127*** | 0.017 |
| Not in labor force |  |  | 0.053*** | 0.004 | 0.012** | 0.004 |
| Household characteristics |  |  |  |  |  |  |
| Housing tenure |  |  |  |  |  |  |
| Owner |  |  | (Reference) | | | |
| Renter |  |  | 0.103*** | 0.006 | 0.076*** | 0.006 |
| Occupy without payment of rent |  |  | 0.085*** | 0.014 | 0.052*** | 0.013 |
| Person with a disability present |  |  | 0.169*** | 0.005 | 0.157*** | 0.005 |
| Person 65 years or older present |  |  | -0.040*** | 0.006 | -0.029*** | 0.006 |
| Child present |  |  | 0.022*** | 0.006 | 0.003 | 0.006 |
| Number of persons present |  |  | 0.009*** | 0.002 | 0.008*** | 0.002 |
| Income-to-poverty ratio |  |  |  |  |  |  |
| Less than 100% |  |  |  |  | (Reference) | |
| 100%-199% |  |  |  |  | -0.028*** | 0.008 |
| 200%-299% |  |  |  |  | -0.088*** | 0.006 |
| 300%-399% |  |  |  |  | -0.129*** | 0.01 |
| More than 400% |  |  |  |  | -0.174*** | 0.009 |

*Supplementary Table 8 (continued). Linear probability regression estimates of the association between metropolitan status and any form of hardship (Table 6 with all covariate coefficients).*

|  | Model 1  (Base) | | Model 2  (Base + controls) | | Model 3  (Full) | |
| --- | --- | --- | --- | --- | --- | --- |
|  | b | se | b | se | b | se |
| Region |  |  |  |  |  |  |
| Northeast |  |  | (Reference) | | | |
| Midwest |  |  | -0.026** | 0.01 | -0.028** | 0.01 |
| South |  |  | -0.014 | 0.01 | -0.022+ | 0.011 |
| West |  |  | -0.019+ | 0.01 | -0.018+ | 0.01 |
| Year |  |  |  |  |  |  |
| 2013 |  |  | (Reference) | | | |
| 2017 |  |  | -0.016** | 0.006 | -0.010+ | 0.006 |
| 2021 |  |  | -0.035*** | 0.006 | -0.027*** | 0.006 |
| Constant | 0.278*** | 0.006 | 0.054** | 0.017 | 0.121*** | 0.017 |
| Observations | 69,735 |  | 69,735 |  | 69,735 |  |
| Note: Coefficients and standard errors shown. *p<.05 ** p<.01 *** p<.001  Source: Survey of Income and Program Participation Waves 2014, 2018, and 2022 | | | | | | |

# Supplementary Table 9. Blinder-Oaxaca decompositions of differences in the prevalence of material hardships across metropolitan and nonmetropolitan households. (Table 7 with all covariate coefficients shown.)

|  | Food | | Bill-paying | | Housing quality | | Any hardship | |
| --- | --- | --- | --- | --- | --- | --- | --- | --- |
|  | b | se | b | se | b | se | b | se |
| Metropolitan status |  |  |  |  |  |  |  |  |
| Metropolitan | 0.108*** | 0.003 | 0.101*** | 0.003 | 0.173*** | 0.005 | 0.278*** | 0.005 |
| Nonmetropolitan | 0.134*** | 0.006 | 0.101*** | 0.005 | 0.187*** | 0.007 | 0.298*** | 0.009 |
| *Difference* | -0.026*** | 0.006 | 0.000 | 0.005 | -0.014* | 0.007 | -0.020* | 0.008 |
| *Explained by compositional effects* | -0.022*** | 0.003 | -0.009** | 0.003 | -0.010* | 0.004 | -0.021*** | 0.005 |
| Householder characteristics |  |  |  |  |  |  |  |  |
| Age | 0.005*** | 0.001 | 0.003*** | 0.001 | 0.004*** | 0.001 | 0.008*** | 0.001 |
| Gender | -0.000* | 0.000 | -0.000* | 0.000 | 0.000 | 0.000 | -0.000* | 0.000 |
| Race and ethnicity | 0.001 | 0.001 | 0.005*** | 0.001 | 0.004* | 0.002 | 0.006*** | 0.002 |
| Born in the U.S. | -0.001 | 0.001 | -0.002* | 0.001 | -0.003* | 0.001 | -0.002** | 0.001 |
| Education attainment | -0.007*** | 0.001 | -0.005*** | 0.001 | -0.002* | 0.001 | -0.008*** | 0.001 |
| Marital status | -0.001* | 0.000 | -0.001* | 0.000 | 0.000 | 0.001 | -0.001 | 0.001 |
| Employment status | -0.002*** | 0.000 | 0.001* | 0.000 | 0.000 | 0.000 | -0.001* | 0.000 |
| Household characteristics |  |  |  |  |  |  |  |  |
| Housing tenure | 0.006*** | 0.001 | 0.004*** | 0.001 | 0.002* | 0.001 | 0.007*** | 0.001 |
| Person with a disability present | -0.010*** | 0.001 | -0.008*** | 0.001 | -0.012*** | 0.001 | -0.017*** | 0.002 |
| Person 65 years or older present | 0.001*** | 0.000 | 0.001*** | 0.000 | 0.001* | 0.000 | 0.001*** | 0.000 |
| Child present | 0.000 | 0.000 | 0.001** | 0.000 | -0.001* | 0.000 | 0.000 | 0.000 |
| Number of persons present | 0.000 | 0.000 | 0.001* | 0.000 | 0.001* | 0.001 | 0.001* | 0.000 |
| Household income-to-poverty ratio | -0.014*** | 0.002 | -0.010*** | 0.001 | -0.007*** | 0.001 | -0.017*** | 0.002 |
| Region | -0.001 | 0.001 | 0.001 | 0.001 | 0.003* | 0.001 | 0.003* | 0.001 |
| Reference year | 0.000 | 0.000 | 0.000 | 0.000 | 0.000 | 0.000 | 0.000 | 0.000 |
| *Unexplained* | -0.003 | 0.004 | 0.009* | 0.004 | -0.004 | 0.006 | 0.001 | 0.006 |
| Householder characteristics |  |  |  |  |  |  |  |  |
| Age | -0.017 | 0.033 | -0.009 | 0.031 | -0.066 | 0.046 | -0.087 | 0.048 |
| Gender | -0.008 | 0.005 | 0.004 | 0.003 | 0.006 | 0.004 | 0.002 | 0.005 |
| Race and ethnicity | 0.005 | 0.007 | -0.010 | 0.007 | -0.001 | 0.009 | -0.003 | 0.009 |
| Born in the U.S. | -0.049** | 0.014 | -0.016 | 0.019 | 0.005 | 0.021 | -0.033 | 0.024 |
| Education attainment | 0.001 | 0.002 | 0.006** | 0.002 | 0.002 | 0.003 | 0.006 | 0.004 |
| Marital status | -0.002 | 0.002 | -0.002 | 0.002 | 0.003 | 0.002 | 0.001 | 0.003 |
| Employment status | 0.009 | 0.010 | -0.003 | 0.010 | 0.014 | 0.009 | 0.001 | 0.010 |
| Household characteristics |  |  |  |  |  |  |  |  |
| Housing tenure | 0.005 | 0.007 | -0.012* | 0.005 | 0.002 | 0.007 | 0.002 | 0.007 |
| Person with a disability present | -0.008 | 0.005 | -0.004 | 0.004 | -0.001 | 0.006 | -0.005 | 0.007 |
| Person 65 years or older present | -0.002 | 0.003 | 0.002 | 0.003 | -0.005 | 0.006 | -0.004 | 0.005 |
| Child present | 0.004 | 0.004 | 0.000 | 0.003 | 0.003 | 0.004 | 0.002 | 0.004 |
| Number of persons present | -0.004 | 0.010 | 0.006 | 0.010 | -0.008 | 0.014 | -0.001 | 0.013 |
| Household income-to-poverty ratio | -0.001 | 0.001 | 0.000 | 0.001 | 0.003* | 0.001 | 0.002 | 0.001 |
| Region | -0.001 | 0.002 | -0.002 | 0.002 | -0.006 | 0.004 | -0.005 | 0.004 |
| Reference year | 0.007* | 0.003 | 0.001 | 0.004 | 0.000 | 0.005 | 0.000 | 0.005 |
| Constant | 0.056 | 0.038 | 0.049 | 0.038 | 0.046 | 0.062 | 0.122* | 0.060 |
| Observations | 69,735 |  | 69,735 |  | 69,735 |  | 69,735 |  |
| Notes: Coefficients and standard errors shown. *p<.05 ** p<.01 *** p<.001  Source: Survey of Income and Program Participation Waves 2014, 2018, and 2022. | | | | | | | | |

# Supplementary Table 10. Linear probability regression estimates of the association between metropolitan status and food hardship using multiple imputation.

|  | Model 1 | | Model 2 | | Model 3 | |
| --- | --- | --- | --- | --- | --- | --- |
|  | (Base) | | (Base + Controls) | | (Full) | |
|  | b | se | b | se | b | se |
| Metropolitan status |  |  |  |  |  |  |
| Metropolitan | (Reference) | | | | | |
| Nonmetropolitan | 0.023*** | 0.006 | 0.013** | 0.004 | 0.004 | 0.004 |
| Householder characteristics |  |  |  |  |  |  |
| Age |  |  | 0.007*** | 0 | 0.008*** | 0 |
| Age^2^ |  |  | -0.000*** | 0 | -0.000*** | 0 |
| Gender |  |  |  |  |  |  |
| Men |  |  | (Reference) | | | |
| Women |  |  | 0.018*** | 0.002 | 0.015*** | 0.002 |
| Race and ethnicity |  |  |  |  |  |  |
| Non-Hispanic white |  |  | (Reference) | | | |
| Non-Hispanic Black |  |  | 0.035*** | 0.006 | 0.027*** | 0.006 |
| Hispanic |  |  | 0.022*** | 0.004 | 0.014** | 0.004 |
| Asian |  |  | -0.017** | 0.005 | -0.018** | 0.006 |
| Other |  |  | 0.050*** | 0.01 | 0.044*** | 0.009 |
| Born in the U.S. |  |  | -0.005 | 0.006 | 0.007 | 0.006 |
| Education attainment |  |  |  |  |  |  |
| High school degree or less |  |  | (Reference) | | | |
| Some college |  |  | -0.022*** | 0.004 | -0.011** | 0.004 |
| Bachelor's degree |  |  | -0.069*** | 0.004 | -0.042*** | 0.004 |
| More than a bachelor's degree |  |  | -0.073*** | 0.004 | -0.040*** | 0.004 |
| Marital status |  |  |  |  |  |  |
| Never married |  |  | (Reference) | | | |
| Married |  |  | -0.039*** | 0.004 | -0.012* | 0.004 |
| Divorced, separated, or widowed |  |  | 0.015** | 0.005 | 0.018** | 0.005 |
| Employment status |  |  |  |  |  |  |
| Employed |  |  | (Reference) | | | |
| Unemployed |  |  | 0.148*** | 0.017 | 0.108*** | 0.017 |
| Not in labor force |  |  | 0.054*** | 0.004 | 0.018*** | 0.004 |
| Household characteristics |  |  |  |  |  |  |
| Housing tenure |  |  |  |  |  |  |
| Owner |  |  | (Reference) | | | |
| Renter |  |  | 0.083*** | 0.004 | 0.060*** | 0.004 |
| Occupy without payment of rent |  |  | 0.040** | 0.014 | 0.011 | 0.013 |
| Person with a disability present |  |  | 0.100*** | 0.003 | 0.090*** | 0.003 |
| Person 65 years or older present |  |  | -0.033*** | 0.004 | -0.023*** | 0.004 |
| Child present |  |  | 0.016*** | 0.004 | 0.001 | 0.004 |
| Number of persons present |  |  | -0.002 | 0.002 | -0.002 | 0.002 |

*Supplementary Table 10 (continued). Linear probability regression estimates of the association between metropolitan status and food hardship using multiple imputation.*

|  | Model 1 | | Model 2 | | Model 3 | |
| --- | --- | --- | --- | --- | --- | --- |
|  | (Base) | | (Base + Controls) | | (Full) | |
|  | b | se | b | se | b | se |
| Household characteristics |  |  |  |  |  |  |
| Household income-to-poverty  ratio |  |  |  |  |  |  |
| Less than 100% |  |  |  |  | (Reference) | |
| 100%-199% |  |  |  |  | -0.021** | 0.007 |
| 200%-299% |  |  |  |  | -0.088*** | 0.006 |
| 300%-399% |  |  |  |  | -0.117*** | 0.006 |
| More than 400% |  |  |  |  | -0.146*** | 0.006 |
| Region |  |  |  |  |  |  |
| Northeast |  |  | (Reference) | | | |
| Midwest |  |  | 0.007 | 0.005 | 0.005 | 0.005 |
| South |  |  | 0.012** | 0.004 | 0.007 | 0.004 |
| West |  |  | -0.002 | 0.005 | -0.001 | 0.005 |
| Year |  |  |  |  |  |  |
| 2013 |  |  | (Reference) | | | |
| 2017 |  |  | -0.025*** | 0.003 | -0.020*** | 0.003 |
| 2021 |  |  | -0.033*** | 0.004 | -0.027*** | 0.003 |
| Constant | 0.108*** | 0.003 | -0.049*** | 0.013 | 0.011 | 0.013 |
| Observations | 73,292 |  | 73,292 |  | 73,292 |  |
| Notes: Coefficients and standard errors shown. *p<.05 ** p<.01 *** p<.001 | | | | | | |

# Supplementary Table 11. Linear probability regression estimates of the association between metropolitan status and bill-paying hardship using multiple imputation.

|  | Model 1 | | Model 2 | | Model 3 | |
| --- | --- | --- | --- | --- | --- | --- |
|  | (Base) | | (Base + Controls) | | (Full) | |
|  | b | se | b | se | b | se |
| Metropolitan status |  |  |  |  |  |  |
| Metropolitan | (Reference) | | | | | |
| Nonmetropolitan | -0.001 | 0.004 | -0.002 | 0.004 | -0.008* | 0.004 |
| Householder characteristics |  |  |  |  |  |  |
| Age |  |  | 0.007*** | 0.001 | 0.007*** | 0.001 |
| Age^2^ |  |  | -0.000*** | 0 | -0.000*** | 0 |
| Gender |  |  |  |  |  |  |
| Men |  |  | (Reference) | | | |
| Women |  |  | 0.018*** | 0.003 | 0.015*** | 0.003 |
| Race and ethnicity |  |  |  |  |  |  |
| Non-Hispanic white |  |  | (Reference) | | | |
| Non-Hispanic Black |  |  | 0.073*** | 0.007 | 0.068*** | 0.007 |
| Hispanic |  |  | 0.036*** | 0.005 | 0.030*** | 0.004 |
| Asian |  |  | -0.013** | 0.005 | -0.014** | 0.005 |
| Other |  |  | 0.052*** | 0.012 | 0.048*** | 0.012 |
| Born in the U.S. |  |  | 0.004 | 0.005 | 0.012* | 0.005 |
| Education attainment |  |  |  |  |  |  |
| High school degree or less |  |  | (Reference) | | | |
| Some college |  |  | -0.001 | 0.003 | 0.007+ | 0.003 |
| Bachelor's degree |  |  | -0.044*** | 0.004 | -0.024*** | 0.004 |
| More than a bachelor's degree |  |  | -0.050*** | 0.003 | -0.026*** | 0.004 |
| Marital status |  |  |  |  |  |  |
| Never married |  |  | (Reference) | | | |
| Married |  |  | -0.032*** | 0.004 | -0.013** | 0.004 |
| Divorced, separated, or widowed |  |  | 0.016** | 0.005 | 0.018*** | 0.005 |
| Employment status |  |  |  |  |  |  |
| Employed |  |  | (Reference) | | | |
| Unemployed |  |  | 0.135*** | 0.012 | 0.108*** | 0.012 |
| Not in labor force |  |  | 0.017*** | 0.003 | -0.008* | 0.003 |
| Household characteristics |  |  |  |  |  |  |
| Housing tenure |  |  |  |  |  |  |
| Owner |  |  | (Reference) | | | |
| Renter |  |  | 0.050*** | 0.004 | 0.035*** | 0.004 |
| Occupy without payment of rent |  |  | 0.017 | 0.011 | -0.003 | 0.011 |
| Person with a disability present |  |  | 0.079*** | 0.003 | 0.072*** | 0.003 |
| Person 65 years or older present |  |  | -0.024*** | 0.003 | -0.018*** | 0.003 |
| Child present |  |  | 0.039*** | 0.004 | 0.028*** | 0.004 |
| Number of persons present |  |  | 0.006*** | 0.001 | 0.006*** | 0.001 |

*Supplementary Table 11 (continued). Linear probability regression estimates of the association between metropolitan status and bill-paying hardship using multiple imputation.*

|  | Model 1 |  | Model 2 |  | Model 3 |  |
| --- | --- | --- | --- | --- | --- | --- |
|  | (Base) |  | (Base + Controls) |  | (Full) |  |
|  | b | se | b | se | b | se |
| Household characteristics |  |  |  |  |  |  |
| Household income-to-poverty ratio |  |  |  |  |  |  |
| Less than 100% |  |  |  |  | (Reference) | |
| 100%-199% |  |  |  |  | -0.020*** | 0.005 |
| 200%-299% |  |  |  |  | -0.057*** | 0.005 |
| 300%-399% |  |  |  |  | -0.075*** | 0.005 |
| More than 400% |  |  |  |  | -0.103*** | 0.006 |
| Region |  |  |  |  |  |  |
| Northeast |  |  | (Reference) | | | |
| Midwest |  |  | -0.019*** | 0.004 | -0.021*** | 0.004 |
| South |  |  | -0.022*** | 0.004 | -0.026*** | 0.005 |
| West |  |  | -0.022*** | 0.004 | -0.022*** | 0.005 |
| Year |  |  |  |  |  |  |
| 2013 |  |  | (Reference) | | | |
| 2017 |  |  | -0.027*** | 0.004 | -0.024*** | 0.004 |
| 2021 |  |  | -0.037*** | 0.004 | -0.033*** | 0.004 |
| Constant | 0.102*** | 0.002 | -0.060*** | 0.014 | -0.019 | 0.014 |
| Observations | 73,292 |  | 73,292 |  | 73,292 |  |
| Notes: Coefficients and standard errors shown. *p<.05 ** p<.01 *** p<.001 | | | | | | |
| Source: Survey of Income and Program Participation Waves 2014, 2018, and 2022 | | | | | | |

# Supplementary Table 12. Linear probability regression estimates of the association between metropolitan status and housing hardship using multiple imputation.

|  | Model 1 | | Model 2 | | Model 3 | |
| --- | --- | --- | --- | --- | --- | --- |
|  | (Base) | | (Base + Controls) | | (Full) | |
|  | b | se | b | se | b | se |
| Metropolitan status |  |  |  |  |  |  |
| Metropolitan | (Reference) | | | | | |
| Nonmetropolitan | 0.015* | 0.006 | 0.010* | 0.005 | 0.006 | 0.005 |
| Householder characteristics |  |  |  |  |  |  |
| Age |  |  | 0.003*** | 0.001 | 0.003*** | 0.001 |
| Age^2^ |  |  | -0.000*** | 0 | -0.000*** | 0 |
| Gender |  |  |  |  |  |  |
| Men |  |  | (Reference) | | | |
| Women |  |  | 0.007* | 0.003 | 0.005+ | 0.003 |
| Race and ethnicity |  |  |  |  |  |  |
| Non-Hispanic white |  |  | (Reference) | | | |
| Non-Hispanic Black |  |  | 0.022** | 0.008 | 0.018* | 0.008 |
| Hispanic |  |  | 0.034** | 0.012 | 0.030* | 0.011 |
| Asian |  |  | 0.017+ | 0.009 | 0.017+ | 0.009 |
| Other |  |  | 0.074*** | 0.013 | 0.071*** | 0.013 |
| Born in the U.S. |  |  | 0.014* | 0.007 | 0.020** | 0.007 |
| Education attainment |  |  |  |  |  |  |
| High school degree or less |  |  | (Reference) | | | |
| Some college |  |  | -0.007+ | 0.004 | -0.002 | 0.004 |
| Bachelor's degree |  |  | -0.027*** | 0.007 | -0.014* | 0.007 |
| More than a bachelor's degree |  |  | -0.021** | 0.007 | -0.005 | 0.006 |
| Marital status |  |  |  |  |  |  |
| Never married |  |  | (Reference) | | | |
| Married |  |  | -0.059*** | 0.006 | -0.046*** | 0.006 |
| Divorced, separated, or widowed |  |  | -0.001 | 0.006 | 0 | 0.006 |
| Employment status |  |  |  |  |  |  |
| Employed |  |  | (Reference) | | | |
| Unemployed |  |  | 0.075*** | 0.012 | 0.056*** | 0.012 |
| Not in labor force |  |  | 0.023*** | 0.003 | 0.006* | 0.002 |
| Household characteristics |  |  |  |  |  |  |
| Housing tenure |  |  |  |  |  |  |
| Owner |  |  | (Reference) | | | |
| Renter |  |  | 0.041*** | 0.007 | 0.030*** | 0.007 |
| Occupy without payment of rent |  |  | 0.067*** | 0.012 | 0.053*** | 0.012 |
| Person with a disability present |  |  | 0.109*** | 0.004 | 0.104*** | 0.004 |
| Person 65 years or older present |  |  | -0.022** | 0.007 | -0.018** | 0.007 |
| Child present |  |  | -0.013* | 0.006 | -0.021** | 0.006 |
| Number of persons present |  |  | 0.012*** | 0.002 | 0.012*** | 0.002 |

*Supplementary Table 12 (continued). Linear probability regression estimates of the association between metropolitan status and housing hardship using multiple imputation.*

|  | Model 1 | | Model 2 | | Model 3 | |
| --- | --- | --- | --- | --- | --- | --- |
|  | (Base) | | (Base + Controls) | | (Full) | |
|  | b | se | b | se | b | se |
| Household characteristics |  |  |  |  |  |  |
| Household income-to-poverty ratio |  |  |  |  |  |  |
| Less than 100% |  |  |  |  | (Reference) | |
| 100%-199% |  |  |  |  | -0.016* | 0.006 |
| 200%-299% |  |  |  |  | -0.036*** | 0.006 |
| 300%-399% |  |  |  |  | -0.049*** | 0.008 |
| More than 400% |  |  |  |  | -0.072*** | 0.007 |
| Region |  |  |  |  |  |  |
| Northeast |  |  | (Reference) | | | |
| Midwest |  |  | -0.022* | 0.01 | -0.024* | 0.009 |
| South |  |  | -0.005 | 0.012 | -0.008 | 0.012 |
| West |  |  | -0.01 | 0.01 | -0.01 | 0.01 |
| Year |  |  |  |  |  |  |
| 2013 |  |  | (Reference) | | | |
| 2017 |  |  | 0.010* | 0.004 | 0.012** | 0.005 |
| 2021 |  |  | -0.002 | 0.006 | 0.002 | 0.006 |
| Constant | 0.173*** | 0.005 | 0.080*** | 0.016 | 0.108*** | 0.017 |
| Observations | 73,292 |  | 73,292 |  | 73,292 |  |
| Notes: Coefficients and standard errors shown. *p<.05 ** p<.01 *** p<.001 | | | | | | |
| Source: Survey of Income and Program Participation Waves 2014, 2018, and 2022 | | | | | | |

# Supplementary Table 13. Linear probability regression estimates of the association between metropolitan status and any form of hardship using multiple imputation.

|  | Model 1 | | Model 2 | | Model 3 | |
| --- | --- | --- | --- | --- | --- | --- |
|  | (Base) | | (Base + Controls) | | (Full) | |
|  | b | se | b | se | b | se |
| Metropolitan status |  |  |  |  |  |  |
| Metropolitan | (Reference) | | | | | |
| Nonmetropolitan | 0.019* | 0.008 | 0.014* | 0.006 | 0.002 | 0.006 |
| Householder characteristics |  |  |  |  |  |  |
| Age |  |  | 0.009*** | 0.001 | 0.010*** | 0.001 |
| Age^2^ |  |  | -0.000*** | 0 | -0.000*** | 0 |
| Gender |  |  |  |  |  |  |
| Men |  |  | (Reference) | | | |
| Women |  |  | 0.026*** | 0.003 | 0.021*** | 0.003 |
| Race and ethnicity |  |  |  |  |  |  |
| Non-Hispanic white |  |  | (Reference) | | | |
| Non-Hispanic Black |  |  | 0.078*** | 0.009 | 0.068*** | 0.009 |
| Hispanic |  |  | 0.060*** | 0.009 | 0.050*** | 0.009 |
| Asian |  |  | -0.006 | 0.007 | -0.007 | 0.007 |
| Other |  |  | 0.109*** | 0.017 | 0.102*** | 0.016 |
| Born in the U.S. |  |  | 0.002 | 0.005 | 0.016** | 0.005 |
| Education attainment |  |  |  |  |  |  |
| High school degree or less |  |  | (Reference) | | | |
| Some college |  |  | -0.017** | 0.006 | -0.003 | 0.005 |
| Bachelor's degree |  |  | -0.079*** | 0.009 | -0.045*** | 0.008 |
| More than a bachelor's degree |  |  | -0.080*** | 0.008 | -0.039*** | 0.007 |
| Marital status |  |  |  |  |  |  |
| Never married |  |  | (Reference) | | | |
| Married |  |  | -0.084*** | 0.007 | -0.051*** | 0.007 |
| Divorced, separated, or widowed |  |  | 0.016* | 0.008 | 0.019** | 0.007 |
| Employment status |  |  |  |  |  |  |
| Employed |  |  | (Reference) | | | |
| Unemployed |  |  | 0.173*** | 0.016 | 0.126*** | 0.016 |
| Not in labor force |  |  | 0.052*** | 0.004 | 0.010** | 0.003 |
| Household characteristics |  |  |  |  |  |  |
| Housing tenure |  |  |  |  |  |  |
| Owner |  |  | (Reference) | | | |
| Renter |  |  | 0.104*** | 0.006 | 0.078*** | 0.006 |
| Occupy without payment of rent |  |  | 0.080*** | 0.013 | 0.047*** | 0.013 |
| Person with a disability present |  |  | 0.168*** | 0.005 | 0.156*** | 0.005 |
| Person 65 years or older present |  |  | -0.042*** | 0.006 | -0.031*** | 0.006 |
| Child present |  |  | 0.020*** | 0.005 | 0.001 | 0.006 |
| Number of persons present |  |  | 0.010*** | 0.002 | 0.010*** | 0.002 |

*Supplementary Table 13 (continued). Linear probability regression estimates of the association between metropolitan status and any form of hardship using multiple imputation.*

|  | Model 1 | | Model 2 | | Model 3 | |
| --- | --- | --- | --- | --- | --- | --- |
|  | (Base) | | (Base + Controls) | | (Full) | |
|  | b | se | b | se | b | se |
| Household characteristics |  |  |  |  |  |  |
| Household income-to-poverty ratio |  |  |  |  |  |  |
| Less than 100% |  |  |  |  | (Reference) | |
| 100%-199% |  |  |  |  | -0.027*** | 0.008 |
| 200%-299% |  |  |  |  | -0.087*** | 0.006 |
| 300%-399% |  |  |  |  | -0.126*** | 0.009 |
| More than 400% |  |  |  |  | -0.174*** | 0.008 |
| Region |  |  |  |  |  |  |
| Northeast |  |  | (Reference) | | | |
| Midwest |  |  | -0.028*** | 0.008 | -0.031*** | 0.008 |
| South |  |  | -0.016+ | 0.008 | -0.023* | 0.009 |
| West |  |  | -0.022* | 0.008 | -0.021* | 0.008 |
| Year |  |  |  |  |  |  |
| 2013 |  |  | (Reference) | | | |
| 2017 |  |  | -0.016** | 0.006 | -0.010+ | 0.006 |
| 2021 |  |  | -0.032*** | 0.006 | -0.024*** | 0.006 |
| Constant | 0.278*** | 0.005 | 0.056** | 0.017 | 0.122*** | 0.017 |
| Observations | 73,292 |  | 73,292 |  | 73,292 |  |
| Notes: Coefficients and standard errors shown. *p<.05 ** p<.01 *** p<.001 | | | | | | |
| Source: Survey of Income and Program Participation Waves 2014, 2018, and 2022 | | | | | | |

# Supplementary Table 14. Blinder-Oaxaca decompositions of differences in the prevalence of material hardships across metropolitan and nonmetropolitan households using multiple imputation.

|  | Food | | Bill-paying | | Housing | | Any Hardship | |
| --- | --- | --- | --- | --- | --- | --- | --- | --- |
|  | b | se | b | se | b | se | b | se |
| Metropolitan status |  |  |  |  |  |  |  |  |
| Metropolitan | 0.108*** | 0.003 | 0.102*** | 0.003 | 0.173*** | 0.004 | 0.279*** | 0.005 |
| Nonmetropolitan | 0.129*** | 0.006 | 0.102*** | 0.005 | 0.185*** | 0.007 | 0.295*** | 0.008 |
| *Difference* | -0.021*** | 0.006 | 0.000 | 0.004 | -0.011 | 0.006 | -0.017* | 0.008 |
| *Explained* | -0.018*** | 0.003 | -0.006* | 0.003 | -0.007 | 0.004 | -0.014** | 0.005 |
| Householder characteristics |  |  |  |  |  |  |  |  |
| Age | 0.005*** | 0.001 | 0.003*** | 0.001 | 0.003*** | 0.001 | 0.007*** | 0.001 |
| Gender | -0.000** | 0.000 | -0.000** | 0.000 | -0.000 | 0.000 | -0.001** | 0.000 |
| Race and ethnicity | 0.001 | 0.001 | 0.005** | 0.001 | 0.003* | 0.002 | 0.006** | 0.002 |
| Born in the U.S. | -0.002 | 0.001 | -0.002** | 0.001 | -0.003** | 0.001 | -0.003** | 0.001 |
| Education attainment | -0.007*** | 0.001 | -0.005*** | 0.001 | -0.003** | 0.001 | -0.008*** | 0.001 |
| Marital status | -0.001* | 0.000 | -0.001* | 0.000 | 0.000 | 0.001 | -0.001 | 0.001 |
| Employment status | -0.002*** | 0.000 | 0.000 | 0.000 | -0.001*** | 0.000 | -0.001** | 0.000 |
| Household characteristics |  |  |  |  |  |  |  |  |
| Housing tenure | 0.006*** | 0.001 | 0.004*** | 0.001 | 0.002* | 0.001 | 0.007*** | 0.001 |
| Household income-to-poverty ratio | -0.013*** | 0.002 | -0.009*** | 0.001 | -0.006*** | 0.001 | -0.016*** | 0.002 |
| Person with a disability present | -0.007*** | 0.001 | -0.004*** | 0.001 | -0.007*** | 0.001 | -0.010*** | 0.001 |
| Person 65 years or older present | 0.001* | 0.000 | 0.000* | 0.000 | 0.000 | 0.000 | 0.001* | 0.000 |
| Child present | 0.000 | 0.000 | 0.001** | 0.000 | -0.001* | 0.000 | 0.000 | 0.000 |
| Number of persons present | 0.001* | 0.000 | 0.001** | 0.001 | 0.003** | 0.001 | 0.003** | 0.001 |
| Region | -0.001 | 0.001 | 0.001 | 0.001 | 0.002* | 0.001 | 0.002 | 0.001 |
| Reference year | 0.000 | 0.000 | 0.000 | 0.000 | 0.000 | 0.000 | 0.000 | 0.000 |
| *Unexplained* | -0.004 | 0.004 | 0.006 | 0.004 | -0.004 | 0.006 | -0.002 | 0.006 |
| Householder characteristics |  |  |  |  |  |  |  |  |
| Age | -0.044 | 0.032 | -0.028 | 0.032 | -0.081 | 0.047 | -0.109* | 0.047 |
| Gender | -0.006 | 0.005 | 0.003 | 0.003 | 0.006 | 0.004 | 0.002 | 0.004 |
| Race and ethnicity | 0.000 | 0.008 | -0.007 | 0.007 | -0.001 | 0.008 | -0.001 | 0.011 |
| Born in the U.S. | -0.032 | 0.019 | -0.016 | 0.017 | 0.004 | 0.019 | -0.028 | 0.028 |
| Education attainment | 0.001 | 0.002 | 0.003 | 0.002 | 0.002 | 0.003 | 0.004 | 0.003 |
| Marital status | -0.003 | 0.002 | -0.001 | 0.002 | 0.002 | 0.002 | 0.001 | 0.003 |
| Employment status | 0.005 | 0.010 | -0.006 | 0.009 | 0.014 | 0.008 | 0.000 | 0.010 |
| Household characteristics |  |  |  |  |  |  |  |  |
| Housing tenure | 0.004 | 0.007 | -0.013* | 0.005 | 0.001 | 0.007 | 0.000 | 0.008 |
| Household income-to-poverty ratio | 0.000 | 0.001 | 0.000 | 0.000 | 0.002* | 0.001 | 0.001 | 0.001 |
| Person with a disability present | 0.009 | 0.020 | -0.006 | 0.021 | -0.022 | 0.022 | 0.000 | 0.024 |
| Person 65 years or older present | -0.003 | 0.003 | 0.003 | 0.003 | -0.003 | 0.006 | -0.003 | 0.005 |
| Child present | 0.003 | 0.003 | 0.000 | 0.003 | 0.001 | 0.004 | 0.001 | 0.004 |
| Number of persons present | -0.005 | 0.010 | 0.000 | 0.011 | -0.001 | 0.014 | -0.003 | 0.013 |
| Region | -0.001 | 0.002 | -0.001 | 0.002 | -0.004 | 0.003 | -0.003 | 0.003 |
| Reference year | 0.000 | 0.000 | 0.000 | 0.000 | 0.000 | 0.000 | 0.000 | 0.000 |
| Constant | 0.067 | 0.047 | 0.075 | 0.04 | 0.076 | 0.066 | 0.134* | 0.062 |
| Observations | 73,345 |  | 73,345 |  | 73,345 |  | 73,345 |  |
| Notes: Coefficients and standard errors shown. *p<.05 ** p<.01 *** p<.001  Source: Survey of Income and Program Participation Waves 2014, 2018, and 2022 | | | | | | | | |

# Supplementary Table 15. Multivariate linear probability regression estimates of the association between metropolitan status and food hardship using an indicator to identify unreported metropolitan status.

|  | Model 1 | | Model 2 | | Model 3 | |
| --- | --- | --- | --- | --- | --- | --- |
|  | (Base) | | (Base + Controls) | | (Full) | |
|  | b | se | b | se | b | se |
| Metropolitan status |  |  |  |  |  |  |
| Metropolitan | (Reference) | | | | | |
| Nonmetropolitan | 0.026*** | 0.006 | 0.014** | 0.005 | 0.004 | 0.004 |
| Unreported | -0.011 | 0.008 | 0.000 | 0.01 | 0.003 | 0.007 |
| Householder characteristics |  |  |  |  |  |  |
| Age |  |  | 0.007*** | 0.000 | 0.008*** | 0.000 |
| Age^2^ |  |  | -0.000*** | 0.000 | -0.000*** | 0.000 |
| Gender |  |  |  |  |  |  |
| Men |  |  | (Reference) | | | |
| Women |  |  | 0.018*** | 0.002 | 0.015*** | 0.002 |
| Race and ethnicity |  |  |  |  |  |  |
| Non-Hispanic white |  |  | (Reference) | | | |
| Non-Hispanic Black |  |  | 0.036*** | 0.005 | 0.027*** | 0.006 |
| Hispanic |  |  | 0.022*** | 0.004 | 0.014** | 0.004 |
| Asian |  |  | -0.017** | 0.005 | -0.018** | 0.006 |
| Other |  |  | 0.050*** | 0.01 | 0.044*** | 0.009 |
| Born in the U.S. |  |  | -0.005 | 0.006 | 0.007 | 0.006 |
| Education attainment |  |  |  |  |  |  |
| High school degree or less |  |  | (Reference) | | | |
| Some college |  |  | -0.022*** | 0.004 | -0.011** | 0.004 |
| Bachelor's degree |  |  | -0.069*** | 0.004 | -0.042*** | 0.004 |
| More than a bachelor's degree |  |  | -0.073*** | 0.004 | -0.040*** | 0.004 |
| Marital status |  |  |  |  |  |  |
| Never married |  |  | (Reference) | | | |
| Married |  |  | -0.039*** | 0.004 | -0.012* | 0.004 |
| Divorced, separated, or widowed |  |  | 0.015** | 0.005 | 0.018** | 0.005 |
| Employment status |  |  |  |  |  |  |
| Employed |  |  | (Reference) | | | |
| Unemployed |  |  | 0.148*** | 0.017 | 0.108*** | 0.017 |
| Not in labor force |  |  | 0.054*** | 0.004 | 0.018*** | 0.004 |
| Household characteristics |  |  |  |  |  |  |
| Housing tenure |  |  |  |  |  |  |
| Owner |  |  | (Reference) | | | |
| Renter |  |  | 0.083*** | 0.004 | 0.060*** | 0.004 |
| Occupy without payment of rent |  |  | 0.040** | 0.014 | 0.011 | 0.013 |
| Person with a disability present |  |  | 0.100*** | 0.003 | 0.090*** | 0.003 |
| Person 65 years or older present |  |  | -0.033*** | 0.004 | -0.023*** | 0.004 |
| Child present |  |  | 0.016*** | 0.004 | 0.001 | 0.004 |
| Number of persons present |  |  | -0.002 | 0.002 | -0.002 | 0.002 |

*Supplementary Table 15 (continued). Multivariate linear probability regression estimates of the association between metropolitan status and food hardship using an indicator to identify unreported metropolitan status.*

|  | Model 1 | | Model 2 | | Model 3 | |
| --- | --- | --- | --- | --- | --- | --- |
|  | (Base) | | (Base + Controls) | | (Full) | |
|  | b | se | b | se | b | se |
| Household characteristics |  |  |  |  |  |  |
| Household income-to-poverty ratio |  |  |  |  |  |  |
| Less than 100% |  |  |  |  | (Reference) | |
| 100%-199% |  |  |  |  | -0.021** | 0.007 |
| 200%-299% |  |  |  |  | -0.088*** | 0.006 |
| 300%-399% |  |  |  |  | -0.117*** | 0.006 |
| More than 400% |  |  |  |  | -0.146*** | 0.006 |
| Region |  |  |  |  |  |  |
| Northeast |  |  | (Reference) | | | |
| Midwest |  |  | 0.006 | 0.005 | 0.005 | 0.005 |
| South |  |  | 0.012* | 0.005 | 0.007 | 0.004 |
| West |  |  | -0.002 | 0.006 | -0.001 | 0.005 |
| Year |  |  |  |  |  |  |
| 2013 |  |  | (Reference) | | | |
| 2017 |  |  | -0.025*** | 0.003 | -0.020*** | 0.003 |
| 2021 |  |  | -0.033*** | 0.004 | -0.027*** | 0.003 |
| Constant | 0.108*** | 0.003 | -0.049*** | 0.013 | 0.01 | 0.013 |
| Observations | 73,292 |  | 73,292 |  | 73,292 |  |
| Coefficients and standard errors shown. *p<.05 ** p<.01 *** p<.001 | | | | | | |
| Source: Survey of Income and Program Participation Waves 2014, 2018, and 2022 | | | | | | |

# Supplementary Table 16. Multivariate linear probability regression estimates of the association between metropolitan status and bill-paying hardship using an indicator to identify unreported metropolitan status.

|  | Model 1 | | Model 2 | | Model 3 | |
| --- | --- | --- | --- | --- | --- | --- |
|  | (Base) | | (Base + Controls) | | (Full) | |
|  | b | se | b | se | b | se |
| Metropolitan status |  |  |  |  |  |  |
| Metropolitan | (Reference) | | | | | |
| Nonmetropolitan | 0.000 | 0.004 | -0.002 | 0.004 | -0.009* | 0.004 |
| Unreported | 0.022* | 0.009 | 0.022** | 0.007 | 0.024** | 0.008 |
| Householder characteristics |  |  |  |  |  |  |
| Age |  |  | 0.007*** | 0.001 | 0.007*** | 0.001 |
| Age^2^ |  |  | -0.000*** | 0 | -0.000*** | 0 |
| Gender |  |  |  |  |  |  |
| Men |  |  | (Reference) | | | |
| Women |  |  | 0.018*** | 0.003 | 0.015*** | 0.003 |
| Racial and ethnic identity |  |  |  |  |  |  |
| Non-Hispanic white |  |  | (Reference) | | | |
| Non-Hispanic Black |  |  | 0.073*** | 0.007 | 0.068*** | 0.007 |
| Hispanic |  |  | 0.037*** | 0.004 | 0.031*** | 0.004 |
| Asian |  |  | -0.013** | 0.005 | -0.014** | 0.005 |
| Other |  |  | 0.052*** | 0.012 | 0.048*** | 0.012 |
| Born in the U.S. |  |  | 0.004 | 0.005 | 0.013* | 0.005 |
| Education attainment |  |  |  |  |  |  |
| High school degree or less |  |  | (Reference) | | | |
| Some college |  |  | -0.001 | 0.003 | 0.007 | 0.003 |
| Bachelor's degree |  |  | -0.044*** | 0.004 | -0.025*** | 0.004 |
| More than a bachelor's degree |  |  | -0.050*** | 0.003 | -0.027*** | 0.004 |
| Marital status |  |  |  |  |  |  |
| Never married |  |  | (Reference) | | | |
| Married |  |  | -0.032*** | 0.004 | -0.013** | 0.004 |
| Divorced, separated, or widowed |  |  | 0.016** | 0.005 | 0.018*** | 0.005 |
| Employment status |  |  |  |  |  |  |
| Employed |  |  | (Reference) | | | |
| Unemployed |  |  | 0.136*** | 0.012 | 0.108*** | 0.012 |
| Not in labor force |  |  | 0.017*** | 0.003 | -0.008* | 0.003 |
| Household characteristics |  |  |  |  |  |  |
| Housing tenure |  |  |  |  |  |  |
| Owner |  |  | (Reference) | | | |
| Renter |  |  | 0.050*** | 0.004 | 0.035*** | 0.004 |
| Occupy without payment of rent |  |  | 0.017 | 0.011 | -0.002 | 0.011 |
| Person with a disability present |  |  | 0.079*** | 0.003 | 0.072*** | 0.003 |
| Person 65 years or older present |  |  | -0.024*** | 0.003 | -0.018*** | 0.003 |
| Child present |  |  | 0.039*** | 0.004 | 0.028*** | 0.004 |
| Number of persons present |  |  | 0.006*** | 0.001 | 0.006*** | 0.001 |

*Supplementary Table 16 (continued). Multivariate linear probability regression estimates of the association between metropolitan status and bill-paying hardship using an indicator to identify unreported metropolitan status.*

|  | Model 1 | | Model 2 | | Model 3 | |
| --- | --- | --- | --- | --- | --- | --- |
|  | (Base) | | (Base + Controls) | | (Full) | |
|  | b | se | b | se | b | se |
| Household characteristics |  |  |  |  |  |  |
| Household income-to-poverty ratio |  |  |  |  |  |  |
| Less than 100% |  |  |  |  | (Reference) | |
| 100%-199% |  |  |  |  | -0.020*** | 0.005 |
| 200%-299% |  |  |  |  | -0.057*** | 0.005 |
| 300%-399% |  |  |  |  | -0.075*** | 0.005 |
| More than 400% |  |  |  |  | -0.104*** | 0.006 |
| Region |  |  |  |  |  |  |
| Northeast |  |  | (Reference) | | | |
| Midwest |  |  | -0.015*** | 0.003 | -0.016*** | 0.003 |
| South |  |  | -0.019*** | 0.003 | -0.022*** | 0.004 |
| West |  |  | -0.019*** | 0.003 | -0.017*** | 0.004 |
| Reference year |  |  |  |  |  |  |
| 2013 |  |  | (Reference) | | | |
| 2017 |  |  | -0.027*** | 0.004 | -0.024*** | 0.004 |
| 2021 |  |  | -0.037*** | 0.004 | -0.033*** | 0.004 |
| Constant | 0.101*** | 0.002 | -0.065*** | 0.014 | -0.024 | 0.013 |
| Observations | 73,292 |  | 73,292 |  | 73,292 |  |
| Coefficients and standard errors shown. *p<.05 ** p<.01 *** p<.001 | | | | | | |
| Source: Survey of Income and Program Participation Waves 2014, 2018, and 2022 | | | | | | |

# Supplementary Table 17. Multivariate linear probability regression estimates of the association between metropolitan status and housing quality hardship using an indicator to identify unreported metropolitan status.

|  | Model 1 | | Model 2 | | Model 3 | |
| --- | --- | --- | --- | --- | --- | --- |
|  | (Base) | | (Base + Controls) | | (Full) | |
|  | b | se | b | se | b | se |
| Metropolitan status |  |  |  |  |  |  |
| Metropolitan | (Reference) | | | | | |
| Nonmetropolitan | 0.014* | 0.007 | 0.01 | 0.005 | 0.005 | 0.005 |
| Unreported | 0.000 | 0.008 | 0.000 | 0.01 | 0.002 | 0.011 |
| Householder characteristics |  |  |  |  |  |  |
| Age |  |  | 0.003*** | 0.001 | 0.003*** | 0.001 |
| Age^2^ |  |  | -0.000*** | 0.000 | -0.000*** | 0.000 |
| Gender |  |  |  |  |  |  |
| Men |  |  | (Reference) | | | |
| Women |  |  | 0.007* | 0.003 | 0.005 | 0.003 |
| Race and ethnicity |  |  |  |  |  |  |
| Non-Hispanic white |  |  | (Reference) | | | |
| Non-Hispanic Black |  |  | 0.022** | 0.008 | 0.018* | 0.008 |
| Hispanic |  |  | 0.034** | 0.012 | 0.030* | 0.011 |
| Asian |  |  | 0.017 | 0.009 | 0.017 | 0.009 |
| Other |  |  | 0.074*** | 0.013 | 0.071*** | 0.013 |
| Born in the U.S. |  |  | 0.014* | 0.007 | 0.020** | 0.007 |
| Education attainment |  |  |  |  |  |  |
| High school degree or less |  |  | (Reference) | | | |
| Some college |  |  | -0.007 | 0.004 | -0.002 | 0.004 |
| Bachelor's degree |  |  | -0.028*** | 0.007 | -0.014* | 0.007 |
| More than a bachelor's degree |  |  | -0.021** | 0.007 | -0.005 | 0.006 |
| Marital status |  |  |  |  |  |  |
| Never married |  |  | (Reference) | | | |
| Married |  |  | -0.059*** | 0.006 | -0.046*** | 0.007 |
| Divorced, separated, or widowed |  |  | -0.001 | 0.006 | 0.000 | 0.006 |
| Employment status |  |  |  |  |  |  |
| Employed |  |  | (Reference) | | | |
| Unemployed |  |  | 0.075*** | 0.012 | 0.056*** | 0.012 |
| Not in labor force |  |  | 0.023*** | 0.003 | 0.006* | 0.002 |
| Household characteristics |  |  |  |  |  |  |
| Housing tenure |  |  |  |  |  |  |
| Owner |  |  | (Reference) | | | |
| Renter |  |  | 0.041*** | 0.007 | 0.030*** | 0.007 |
| Occupy without payment of rent |  |  | 0.067*** | 0.012 | 0.053*** | 0.012 |
| Person with a disability present |  |  | 0.109*** | 0.004 | 0.104*** | 0.004 |
| Person 65 years or older present |  |  | -0.022** | 0.007 | -0.018** | 0.007 |
| Child present |  |  | -0.013* | 0.006 | -0.021** | 0.006 |
| Number of persons present |  |  | 0.012*** | 0.002 | 0.012*** | 0.002 |

*Supplementary Table 17 (continued). Multivariate linear probability regression estimates of the association between metropolitan status and housing quality hardship using an indicator to identify unreported metropolitan status.*

|  | Model 1 | | Model 2 | | Model 3 | |
| --- | --- | --- | --- | --- | --- | --- |
|  | (Base) | | (Base + Controls) | | (Full) | |
|  | b | se | b | se | b | se |
| Household income-to-poverty ratio |  |  |  |  |  |  |
| Less than 100% |  |  |  |  | (Reference) | |
| 100%-199% |  |  |  |  | -0.016* | 0.006 |
| 200%-299% |  |  |  |  | -0.036*** | 0.006 |
| 300%-399% |  |  |  |  | -0.049*** | 0.008 |
| More than 400% |  |  |  |  | -0.072*** | 0.007 |
| Region |  |  |  |  |  |  |
| Northeast |  |  | (Reference) | | | |
| Midwest |  |  | -0.023* | 0.011 | -0.023* | 0.011 |
| South |  |  | -0.005 | 0.012 | -0.008 | 0.013 |
| West |  |  | -0.01 | 0.011 | -0.009 | 0.012 |
| Reference year |  |  |  |  |  |  |
| 2013 |  |  | (Reference) | | | |
| 2017 |  |  | 0.010* | 0.004 | 0.012** | 0.005 |
| 2021 |  |  | -0.002 | 0.006 | 0.002 | 0.006 |
| Constant | 0.173*** | 0.005 | 0.081*** | 0.017 | 0.108*** | 0.018 |
| Observations | 73292 |  | 73292 |  | 73292 |  |
| Coefficients and standard errors shown. *p<.05 ** p<.01 *** p<.001 | | | | | | |
| Source: Survey of Income and Program Participation Waves 2014, 2018, and 2022 | | | | | | |

# Supplementary Table 18. Multivariate linear probability regression estimates of the association between metropolitan status and any form of hardship using an indicator to identify unreported metropolitan status.

|  | Model 1 | | Model 2 | | Model 3 | |
| --- | --- | --- | --- | --- | --- | --- |
|  | (Base) | | (Base + Controls) | | (Full) | |
|  | b | se | b | se | b | se |
| Metropolitan status |  |  |  |  |  |  |
| Metropolitan | (Reference) | | | | | |
| Nonmetropolitan | 0.020* | 0.008 | 0.013* | 0.006 | 0.000 | 0.006 |
| Unreported | 0.008 | 0.008 | 0.013 | 0.007 | 0.017 | 0.009 |
| Householder characteristics |  |  |  |  |  |  |
| Age |  |  | 0.009*** | 0.001 | 0.010*** | 0.001 |
| Age^2^ |  |  | -0.000*** | 0.000 | -0.000*** | 0.000 |
| Gender |  |  |  |  |  |  |
| Men |  |  | (Reference) | | | |
| Women |  |  | 0.026*** | 0.003 | 0.021*** | 0.003 |
| Race and ethnicity |  |  |  |  |  |  |
| Non-Hispanic white |  |  | (Reference) | | | |
| Non-Hispanic Black |  |  | 0.078*** | 0.009 | 0.068*** | 0.009 |
| Hispanic |  |  | 0.061*** | 0.009 | 0.050*** | 0.009 |
| Asian |  |  | -0.006 | 0.007 | -0.007 | 0.007 |
| Other |  |  | 0.109*** | 0.017 | 0.102*** | 0.016 |
| Born in the U.S. |  |  | 0.002 | 0.005 | 0.016** | 0.005 |
| Education attainment |  |  |  |  |  |  |
| High school degree or less |  |  | (Reference) | | | |
| Some college |  |  | -0.017** | 0.006 | -0.003 | 0.005 |
| Bachelor's degree |  |  | -0.079*** | 0.009 | -0.046*** | 0.008 |
| More than a bachelor's degree |  |  | -0.081*** | 0.008 | -0.040*** | 0.007 |
| Marital status |  |  |  |  |  |  |
| Never married |  |  | (Reference) | | | |
| Married |  |  | -0.084*** | 0.007 | -0.051*** | 0.007 |
| Divorced, separated, or widowed |  |  | 0.016* | 0.008 | 0.019** | 0.007 |
| Employment status |  |  |  |  |  |  |
| Employed |  |  | (Reference) | | | |
| Unemployed |  |  | 0.173*** | 0.016 | 0.126*** | 0.016 |
| Not in labor force |  |  | 0.052*** | 0.004 | 0.010** | 0.003 |
| Household characteristics |  |  |  |  |  |  |
| Housing tenure |  |  |  |  |  |  |
| Owner |  |  | (Reference) | | | |
| Renter |  |  | 0.104*** | 0.006 | 0.078*** | 0.006 |
| Occupy without payment of rent |  |  | 0.080*** | 0.013 | 0.047*** | 0.013 |
| Person with a disability present |  |  | 0.168*** | 0.005 | 0.156*** | 0.005 |
| Person 65 years or older present |  |  | -0.042*** | 0.006 | -0.031*** | 0.006 |
| Child present |  |  | 0.020*** | 0.006 | 0.001 | 0.006 |
| Number of persons present |  |  | 0.010*** | 0.002 | 0.010*** | 0.002 |

*Supplementary Table 18 (continued). Multivariate linear probability regression estimates of the association between metropolitan status and any form of hardship using an indicator to identify unreported metropolitan status.*

|  | Model 1 | | Model 2 | | Model 3 | |
| --- | --- | --- | --- | --- | --- | --- |
|  | (Base) | | (Base + Controls) | | (Full) | |
|  | b | se | b | se | b | se |
| Household characteristics |  |  |  |  |  |  |
| Household income-to-poverty ratio |  |  |  |  |  |  |
| Less than 100% |  |  |  |  | (Reference) | |
| 100%-199% |  |  |  |  | -0.027*** | 0.008 |
| 200%-299% |  |  |  |  | -0.087*** | 0.006 |
| 300%-399% |  |  |  |  | -0.126*** | 0.009 |
| More than 400% |  |  |  |  | -0.175*** | 0.008 |
| Region |  |  |  |  |  |  |
| Northeast |  |  | (Reference) | | | |
| Midwest |  |  | -0.026** | 0.009 | -0.027** | 0.009 |
| South |  |  | -0.014 | 0.009 | -0.021* | 0.01 |
| West |  |  | -0.020* | 0.009 | -0.018 | 0.01 |
| Reference year |  |  |  |  |  |  |
| 2013 |  |  | (Reference) | | | |
| 2014 |  |  | -0.016** | 0.006 | -0.01 | 0.006 |
| 2021 |  |  | -0.032*** | 0.006 | -0.024*** | 0.006 |
| Constant | 0.278*** | 0.006 | 0.054** | 0.017 | 0.119*** | 0.017 |
| Observations | 73,292 |  | 73,292 |  | 73,292 |  |
| Coefficients and standard errors shown. *p<.05 ** p<.01 *** p<.001 | | | | | | |

# Supplementary Table 19. Linear probability regression estimates of the association between metropolitan status and food hardship excluding data about 2021.

|  | Model 1 | | Model 2 | | Model 3 | |
| --- | --- | --- | --- | --- | --- | --- |
|  | (Base) | | (Base + controls) | | (Full) | |
|  | b | se | b | se | b | se |
| Metropolitan status |  |  |  |  |  |  |
| Metropolitan | (Reference) | | | | | |
| Nonmetropolitan | 0.026*** | 0.007 | 0.014* | 0.005 | 0.002 | 0.005 |
| Householder characteristics |  |  |  |  |  |  |
| Age |  |  | 0.009*** | 0.001 | 0.010*** | 0.001 |
| Age-squared |  |  | -0.000*** | 0.000 | -0.000*** | 0.000 |
| Gender |  |  |  |  |  |  |
| Men |  |  | (Reference) | | | |
| Women |  |  | 0.020*** | 0.003 | 0.016*** | 0.003 |
| Race and ethnicity |  |  |  |  |  |  |
| Non-Hispanic white |  |  | (Reference) | | | |
| Non-Hispanic Black |  |  | 0.035*** | 0.005 | 0.026*** | 0.005 |
| Hispanic |  |  | 0.013** | 0.004 | 0.004 | 0.004 |
| Asian |  |  | -0.028*** | 0.005 | -0.030*** | 0.005 |
| Other |  |  | 0.049*** | 0.01 | 0.041*** | 0.010 |
| Born in the U.S. |  |  | -0.003 | 0.005 | 0.012* | 0.005 |
| Education attainment |  |  |  |  |  |  |
| High school degree or less |  |  | (Reference) | | | |
| Some college |  |  | -0.022*** | 0.004 | -0.008* | 0.004 |
| Bachelor's degree |  |  | -0.071*** | 0.004 | -0.040*** | 0.004 |
| More than a bachelor's degree |  |  | -0.076*** | 0.004 | -0.039*** | 0.004 |
| Marital status |  |  |  |  |  |  |
| Never married |  |  | (Reference) | | | |
| Married |  |  | -0.048*** | 0.005 | -0.016** | 0.006 |
| Divorced, separated, or widowed |  |  | 0.014* | 0.006 | 0.018** | 0.006 |
| Household characteristics |  |  |  |  |  |  |
| Housing tenure |  |  | (Reference) | | | |
| Owner |  |  | 0.139*** | 0.019 | 0.065*** | 0.003 |
| Renter |  |  | 0.054*** | 0.005 | 0.007 | 0.013 |
| Occupy without payment of rent |  |  |  |  |  |  |
| Employment status |  |  |  |  |  |  |
| Employed |  |  | (Reference) | | | |
| Unemployed |  |  | 0.089*** | 0.004 | 0.096*** | 0.017 |
| Not in labor force |  |  | 0.038** | 0.013 | 0.017*** | 0.004 |
| Person with a disability present |  |  | 0.107*** | 0.004 | 0.096*** | 0.003 |
| Person 65 years or older present |  |  | -0.035*** | 0.004 | -0.022*** | 0.004 |
| Child present |  |  | 0.022*** | 0.004 | 0.005 | 0.004 |
| Number of persons present |  |  | -0.001 | 0.002 | -0.002 | 0.002 |

Supplementary Table 19 (continued). Linear probability regression estimates of the association between metropolitan status and food hardship excluding data about 2021.

|  | Model 1 | | Model 2 | | Model 3 | |
| --- | --- | --- | --- | --- | --- | --- |
|  | (Base) | | (Base + controls) | | (Full) | |
|  | b | se | b | se | b | se |
| Household characteristics (continued) | |  |  |  |  |  |
| Household income-to-poverty ratio | |  |  |  |  |  |
| Less than 100% |  |  |  |  | (Reference) | |
| 100%-199% |  |  |  |  | -0.020** | 0.006 |
| 200%-299% |  |  |  |  | -0.092*** | 0.007 |
| 300%-399% |  |  |  |  | -0.133*** | 0.008 |
| More than 400% |  |  |  |  | -0.156*** | 0.008 |
| Region |  |  |  |  |  |  |
| Northeast |  |  | (Reference) | | | |
| Midwest |  |  | 0.005 | 0.007 | 0.002 | 0.006 |
| South |  |  | 0.012 | 0.006 | 0.006 | 0.006 |
| West |  |  | 0.000 | 0.007 | 0.000 | 0.006 |
| Reference year |  |  |  |  |  |  |
| 2013 |  |  | (Reference) | | | |
| 2017 |  |  | -0.025*** | 0.003 | -0.020*** | 0.003 |
| Constant | 0.116*** | 0.003 | -0.083*** | 0.015 | -0.025+ | 0.014 |
| Observations | 53,247 |  | 53,247 |  | 53,247 |  |
| Notes: Coefficients and standard errors shown. *p<.05 ** p<.01 *** p<.001 | | | | | | |
| Source: Data from the Survey of Income and Program Participation Waves 2014 and 2018. | | | | | | |

# Supplementary Table 20. Linear probability regression estimates of the association between metropolitan status and bill-paying hardship excluding data about 2021.

|  | Model 1 | | Model 2 | | Model 3 | |
| --- | --- | --- | --- | --- | --- | --- |
|  | (Base) | | (Base + controls) | | (Full) | |
|  | b | se | b | se | b | se |
| Metropolitan status |  |  |  |  |  |  |
| Metropolitan | (Reference) | | | | | |
| Nonmetropolitan | 0.001 | 0.005 | -0.004 | 0.005 | -0.012* | 0.005 |
| Householder characteristics |  |  |  |  |  |  |
| Age |  |  | 0.007*** | 0.001 | 0.008*** | 0.001 |
| Age^2^ |  |  | -0.000*** | 0 | -0.000*** | 0 |
| Gender |  |  |  |  |  |  |
| Men |  |  | (Reference) | | | |
| Women |  |  | 0.020*** | 0.003 | 0.017*** | 0.003 |
| Race and ethnicity |  |  |  |  |  |  |
| Non-Hispanic white |  |  | (Reference) | | | |
| Non-Hispanic Black |  |  | 0.069*** | 0.007 | 0.062*** | 0.007 |
| Hispanic |  |  | 0.035*** | 0.006 | 0.028*** | 0.006 |
| Asian |  |  | -0.018** | 0.006 | -0.019** | 0.006 |
| Other |  |  | 0.043*** | 0.011 | 0.038*** | 0.011 |
| Born in the U.S. |  |  | 0.008 | 0.006 | 0.018** | 0.006 |
| Education attainment |  |  |  |  |  |  |
| High school degree or less |  |  | (Reference) | | | |
| Some college |  |  | -0.005 | 0.004 | 0.005 | 0.004 |
| Bachelor's degree |  |  | -0.051*** | 0.005 | -0.029*** | 0.005 |
| More than a bachelor's degree |  |  | -0.060*** | 0.004 | -0.033*** | 0.004 |
| Marital status |  |  |  |  |  |  |
| Never married |  |  | (Reference) | | | |
| Married |  |  | -0.032*** | 0.006 | -0.01 | 0.006 |
| Divorced, separated, or widowed |  |  | 0.019** | 0.007 | 0.022** | 0.007 |
| Employment status |  |  |  |  |  |  |
| Employed |  |  | (Reference) | | | |
| Unemployed |  |  | 0.122*** | 0.014 | 0.093*** | 0.014 |
| Not in labor force |  |  | 0.015*** | 0.004 | -0.011* | 0.004 |
| Household characteristics |  |  |  |  |  |  |
| Housing tenure |  |  |  |  |  |  |
| Owner |  |  | (Reference) | | | |
| Renter |  |  | 0.046*** | 0.005 | 0.029*** | 0.005 |
| Occupy without payment of rent |  |  | 0.024* | 0.011 | 0.003 | 0.011 |
| Person with a disability present |  |  | 0.086*** | 0.004 | 0.078*** | 0.004 |
| Person 65 years or older present |  |  | -0.025*** | 0.004 | -0.017*** | 0.004 |
| Child present |  |  | 0.036*** | 0.005 | 0.024*** | 0.005 |
| Number of persons present |  |  | 0.008*** | 0.001 | 0.007*** | 0.001 |

Supplementary Table 20 (continued). Linear probability regression estimates of the association between metropolitan status and bill-paying hardship excluding data about 2021.

|  | Model 1 | | Model 2 | | Model 3 | |
| --- | --- | --- | --- | --- | --- | --- |
|  | (Base) | | (Base + controls) | | (Full) | |
|  | b | se | b | se | b | se |
| Household characteristics |  |  |  |  |  |  |
| Household income-to-poverty ratio |  |  |  |  |  |  |
| Less than 100% |  |  |  |  | (Reference) | |
| 100%-199% |  |  |  |  | -0.020** | 0.007 |
| 200%-299% |  |  |  |  | -0.052*** | 0.006 |
| 300%-399% |  |  |  |  | -0.081*** | 0.006 |
| More than 400% |  |  |  |  | -0.113*** | 0.007 |
| Region |  |  |  |  |  |  |
| Northeast |  |  | (Reference) | | | |
| Midwest |  |  | -0.016** | 0.005 | -0.018*** | 0.005 |
| South |  |  | -0.021*** | 0.005 | -0.026*** | 0.005 |
| West |  |  | -0.022*** | 0.005 | -0.022*** | 0.005 |
| Reference year |  |  |  |  |  |  |
| 2013 |  |  | (Reference) | | | |
| 2017 |  |  | -0.027*** | 0.004 | -0.023*** | 0.004 |
| Constant | 0.109*** | 0.002 | -0.080*** | 0.02 | -0.042* | 0.021 |
| Observations | 53247 |  | 53247 |  | 53247 |  |
| Coefficients and standard errors shown. *p<.05 ** p<.01 *** p<.001 | | | | | | |
| Source: Survey of Income and Program Participation Waves 2014, 2018, and 2022 | | | | | | |

# Supplementary Table 21. Linear probability regression estimates of the association between metropolitan status and housing hardship excluding data about 2021.

|  | Model 1 | | Model 2 | | Model 3 | |
| --- | --- | --- | --- | --- | --- | --- |
|  | (Base) | | (Base + controls) | | (Full) | |
|  | b | se | b | se | b | se |
| Metropolitan status |  |  |  |  |  |  |
| Metropolitan | (Reference) | | | | | |
| Nonmetropolitan | 0.012 | 0.007 | 0.007 | 0.007 | 0.002 | 0.007 |
| Householder characteristics |  |  |  |  |  |  |
| Age |  |  | 0.003*** | 0.001 | 0.003*** | 0.001 |
| Age^2^ |  |  | -0.000*** | 0.000 | -0.000*** | 0.000 |
| Gender |  |  |  |  |  |  |
| Men |  |  | (Reference) | | | |
| Women |  |  | 0.007 | 0.004 | 0.005 | 0.004 |
| Race and ethnicity |  |  |  |  |  |  |
| Non-Hispanic white |  |  | (Reference) | | | |
| Non-Hispanic Black |  |  | 0.025** | 0.008 | 0.021* | 0.009 |
| Hispanic |  |  | 0.026 | 0.014 | 0.021 | 0.014 |
| Asian |  |  | 0.006 | 0.01 | 0.005 | 0.01 |
| Other |  |  | 0.069*** | 0.018 | 0.065*** | 0.018 |
| Born in the U.S. |  |  | 0.004 | 0.006 | 0.01 | 0.006 |
| Education attainment |  |  |  |  |  |  |
| High school degree or less |  |  | (Reference) | | | |
| Some college |  |  | -0.010* | 0.004 | -0.003 | 0.004 |
| Bachelor's degree |  |  | -0.034*** | 0.009 | -0.018* | 0.008 |
| More than a bachelor's degree |  |  | -0.028*** | 0.006 | -0.009 | 0.005 |
| Marital status |  |  |  |  |  |  |
| Never married |  |  | (Reference) | | | |
| Married |  |  | -0.057*** | 0.007 | -0.042*** | 0.007 |
| Divorced, separated, or widowed |  |  | 0.003 | 0.006 | 0.005 | 0.006 |
| Employment status |  |  |  |  |  |  |
| Employed |  |  | (Reference) | | | |
| Unemployed |  |  | 0.071*** | 0.015 | 0.051** | 0.015 |
| Not in labor force |  |  | 0.023*** | 0.004 | 0.005 | 0.003 |
| Household characteristics |  |  |  |  |  |  |
| Housing tenure |  |  |  |  |  |  |
| Owner |  |  | (Reference) | | | |
| Renter |  |  | 0.037*** | 0.01 | 0.026* | 0.01 |
| Occupy without payment of rent |  |  | 0.060*** | 0.011 | 0.046*** | 0.01 |
| Person with a disability present |  |  | 0.105*** | 0.004 | 0.100*** | 0.004 |
| Person 65 years or older present |  |  | -0.015 | 0.009 | -0.01 | 0.009 |
| Child present |  |  | -0.016*** | 0.005 | -0.025*** | 0.004 |
| Number of persons present |  |  | 0.012*** | 0.003 | 0.011*** | 0.003 |

Supplementary Table 21 (continued). Linear probability regression estimates of the association between metropolitan status and housing hardship excluding data about 2021.

|  | Model 1 | | Model 2 | | Model 3 | |
| --- | --- | --- | --- | --- | --- | --- |
|  | (Base) | | (Base + controls) | | (Full) | |
|  | b | se | b | se | b | se |
| Household characteristics |  |  |  |  |  |  |
| Household income-to-poverty ratio |  |  |  |  |  |  |
| Less than 100% |  |  |  |  | (Reference) | |
| 100%-199% |  |  |  |  | -0.015 | 0.008 |
| 200%-299% |  |  |  |  | -0.035*** | 0.007 |
| 300%-399% |  |  |  |  | -0.054*** | 0.008 |
| More than 400% |  |  |  |  | -0.079*** | 0.008 |
| Region |  |  |  |  |  |  |
| Northeast |  |  | (Reference) | | | |
| Midwest |  |  | -0.025 | 0.015 | -0.026 | 0.015 |
| South |  |  | -0.008 | 0.016 | -0.012 | 0.016 |
| West |  |  | -0.011 | 0.016 | -0.011 | 0.016 |
| Reference year |  |  |  |  |  |  |
| 2013 |  |  | (Reference) | | | |
| 2017 |  |  | 0.008* | 0.004 | 0.011* | 0.004 |
| Constant | 0.175*** | 0.006 | 0.110*** | 0.015 | 0.137*** | 0.016 |
| Observations | 53,247 |  | 53,247 |  | 53,247 |  |
| Coefficients and standard errors shown. *p<.05 ** p<.01 *** p<.001  Source: Survey of Income and Program Participation Waves 2014 and 2018 | | | | | | |

# Supplementary Table 22. Linear probability regression estimates of the association between metropolitan status and any form of hardship excluding data about 2021.

|  | Model 1 | | Model 2 | | Model 3 | |
| --- | --- | --- | --- | --- | --- | --- |
|  | (Base) | | (Base + controls) | | (Full) | |
|  | b | se | b | se | b | se |
|  |  |  |  |  |  |  |
| Metropolitan | (Reference) | | | | | |
| Nonmetropolitan | 0.018* | 0.008 | 0.008 | 0.006 | -0.006 | 0.006 |
| Householder characteristics |  |  |  |  |  |  |
| Age |  |  | 0.010*** | 0.001 | 0.011*** | 0.001 |
| Age^2^ |  |  | -0.000*** | 0.000 | -0.000*** | 0.000 |
| Gender |  |  |  |  |  |  |
| Men |  |  | (Reference) | | | |
| Women |  |  | 0.028*** | 0.005 | 0.023*** | 0.005 |
| Race and ethnicity |  |  |  |  |  |  |
| Non-Hispanic white |  |  | (Reference) | | | |
| Non-Hispanic Black |  |  | 0.078*** | 0.01 | 0.068*** | 0.01 |
| Hispanic |  |  | 0.046*** | 0.009 | 0.035*** | 0.009 |
| Asian |  |  | -0.026* | 0.011 | -0.028* | 0.011 |
| Other |  |  | 0.098*** | 0.02 | 0.090*** | 0.019 |
| Born in the U.S. |  |  | -0.001 | 0.006 | 0.016* | 0.007 |
| Education attainment |  |  |  |  |  |  |
| High school degree or less |  |  | (Reference) | | | |
| Some college |  |  | -0.024*** | 0.005 | -0.007 | 0.005 |
| Bachelor's degree |  |  | -0.092*** | 0.01 | -0.054*** | 0.009 |
| More than a bachelor's degree |  |  | -0.097*** | 0.007 | -0.050*** | 0.007 |
| Marital status |  |  |  |  |  |  |
| Never married |  |  | (Reference) | | | |
| Married |  |  | -0.087*** | 0.008 | -0.049*** | 0.007 |
| Divorced, separated, or widowed |  |  | 0.018* | 0.008 | 0.023** | 0.008 |
| Employment status |  |  |  |  |  |  |
| Employed |  |  | (Reference) | | | |
| Unemployed |  |  | 0.159*** | 0.019 | 0.111*** | 0.019 |
| Not in labor force |  |  | 0.051*** | 0.004 | 0.009* | 0.004 |
| Household characteristics |  |  |  |  |  |  |
| Housing tenure |  |  |  |  |  |  |
| Owner |  |  | (Reference) | | | |
| Renter |  |  | 0.102*** | 0.009 | 0.074*** | 0.009 |
| Occupy without payment of rent |  |  | 0.077*** | 0.014 | 0.042** | 0.014 |
| Person with a disability present |  |  | 0.169*** | 0.005 | 0.156*** | 0.005 |
| Person 65 years or older present |  |  | -0.042*** | 0.009 | -0.029** | 0.008 |
| Child present |  |  | 0.018** | 0.005 | -0.003 | 0.005 |
| Number of persons present |  |  | 0.011*** | 0.002 | 0.010*** | 0.002 |

Supplementary Table 22 (continued). Linear probability regression estimates of the association between metropolitan status and any form of hardship excluding data about 2021.

|  | Model 1 | | Model 2 | | Model 3 | |
| --- | --- | --- | --- | --- | --- | --- |
|  | (Base) | | (Base + controls) | | (Full) | |
|  | b | se | b | se | b | se |
| Household characteristics |  |  |  |  |  |  |
| Household income-to-poverty ratio | |  |  |  |  |  |
| Less than 100% |  |  |  |  | (Reference) | |
| 100%-199% |  |  |  |  | -0.027** | 0.009 |
| 200%-299% |  |  |  |  | -0.084*** | 0.006 |
| 300%-399% |  |  |  |  | -0.137*** | 0.008 |
| More than 400% |  |  |  |  | -0.187*** | 0.01 |
| Region |  |  |  |  |  |  |
| Northeast |  |  | (Reference) | | | |
| Midwest |  |  | -0.024** | 0.008 | -0.028*** | 0.008 |
| South |  |  | -0.014 | 0.007 | -0.022* | 0.008 |
| West |  |  | -0.019 | 0.009 | -0.019* | 0.009 |
| Reference year |  |  |  |  |  |  |
| 2013 |  |  | (Reference) | | | |
| 2017 |  |  | -0.016** | 0.006 | -0.009 | 0.006 |
| Constant | 0.287*** | 0.005 | 0.052* | 0.021 | 0.113*** | 0.022 |
| Observations | 53,247 |  | 53,247 |  | 53,247 |  |
| Coefficients and standard errors shown. *p<.05 ** p<.01 *** p<.001  Source: Survey of Income and Program Participation Waves 2014, 2018, and 2022 | | | | | | |

# Supplementary Table 23. Blinder-Oaxaca decompositions of differences in the prevalence of material hardships across metropolitan and nonmetropolitan households excluding data about 2021.

|  | Food | | Bill-paying | | Housing | | Any Hardship | |
| --- | --- | --- | --- | --- | --- | --- | --- | --- |
|  | b | se | b | se | b | se | b | se |
| Metropolitan status |  |  |  |  |  |  |  |  |
| Metropolitan | 0.116*** | 0.003 | 0.109*** | 0.003 | 0.175*** | 0.005 | 0.287*** | 0.005 |
| Nonmetropolitan | 0.141*** | 0.007 | 0.110*** | 0.005 | 0.187*** | 0.007 | 0.305*** | 0.009 |
| *Difference* | -0.026*** | 0.006 | -0.001 | 0.006 | -0.012 | 0.007 | -0.018* | 0.008 |
| *Explained* | -0.024*** | 0.004 | -0.013*** | 0.003 | -0.010* | 0.005 | -0.024*** | 0.006 |
| Householder characteristics |  |  |  |  |  |  |  |  |
| Age | 0.006*** | 0.001 | 0.004*** | 0.001 | 0.005*** | 0.001 | 0.008*** | 0.001 |
| Gender | 0.000 | 0.000 | 0.000 | 0.000 | 0.000 | 0.000 | 0.000 | 0.000 |
| Race and ethnicity | 0.000 | 0.001 | 0.004** | 0.001 | 0.002 | 0.002 | 0.004* | 0.002 |
| Born in the U.S. | -0.002* | 0.001 | -0.002* | 0.001 | -0.001 | 0.001 | -0.002* | 0.001 |
| Education attainment | -0.006*** | 0.001 | -0.005*** | 0.001 | -0.002* | 0.001 | -0.008*** | 0.001 |
| Marital status | -0.001 | 0.000 | -0.001* | 0.000 | 0.000 | 0.001 | -0.001 | 0.001 |
| Employment status | -0.001** | 0.000 | 0.001* | 0.000 | 0.000 | 0.000 | -0.001 | 0.000 |
| Household characteristics |  |  |  |  |  |  |  |  |
| Housing tenure | 0.007*** | 0.001 | 0.003*** | 0.001 | 0.002 | 0.001 | 0.007*** | 0.001 |
| Person with a disability present | -0.011*** | 0.001 | -0.009*** | 0.001 | -0.012*** | 0.001 | -0.018*** | 0.002 |
| Person 65 years or older present | 0.001*** | 0.000 | 0.001** | 0.000 | 0.000 | 0.000 | 0.001** | 0.000 |
| Child present | 0.000 | 0.000 | 0.001** | 0.000 | -0.001** | 0.000 | 0.000 | 0.000 |
| Number of persons present | 0.000 | 0.000 | 0.001** | 0.000 | 0.001* | 0.001 | 0.001** | 0.000 |
| Household income-to-poverty  ratio | -0.015*** | 0.002 | -0.011*** | 0.001 | -0.008*** | 0.001 | -0.018*** | 0.002 |
| Region | -0.001 | 0.001 | 0.001 | 0.001 | 0.003 | 0.002 | 0.003 | 0.002 |
| Reference year | 0.000 | 0.000 | 0.000 | 0.000 | 0.000 | 0.000 | 0.000 | 0.000 |
| *Unexplained* | -0.002 | 0.005 | 0.012* | 0.005 | -0.002 | 0.008 | 0.006 | 0.006 |
| Householder characteristics |  |  |  |  |  |  |  |  |
| Age | 0.022 | 0.041 | 0.04 | 0.03 | -0.015 | 0.046 | -0.015 | 0.046 |
| Gender | -0.006 | 0.005 | 0.005 | 0.003 | 0.003 | 0.004 | 0.003 | 0.004 |
| Race and ethnicity | -0.001 | 0.007 | -0.006 | 0.008 | -0.012 | 0.013 | -0.019 | 0.011 |
| Born in the U.S. | -0.042** | 0.013 | -0.021 | 0.023 | -0.013 | 0.021 | -0.044 | 0.022 |
| Education attainment | 0.001 | 0.002 | 0.006* | 0.002 | 0.001 | 0.004 | 0.005 | 0.004 |
| Marital status | -0.005 | 0.002 | -0.005 | 0.003 | 0.002 | 0.002 | -0.002 | 0.003 |
| Employment status | 0.015 | 0.009 | 0.003 | 0.009 | 0.015 | 0.01 | 0.006 | 0.009 |
| Household characteristics |  |  |  |  |  |  |  |  |
| Housing tenure | -0.002 | 0.007 | -0.01 | 0.006 | -0.015 | 0.01 | -0.015 | 0.009 |
| Person with a disability present | -0.004 | 0.005 | -0.006 | 0.005 | -0.001 | 0.004 | -0.005 | 0.006 |
| Person 65 years or older present | -0.005 | 0.004 | 0.005 | 0.003 | -0.002 | 0.005 | -0.002 | 0.005 |
| Child present | 0.001 | 0.004 | 0.001 | 0.003 | 0.004 | 0.004 | 0.003 | 0.005 |
| Number of persons present | -0.004 | 0.01 | 0.01 | 0.008 | -0.01 | 0.017 | -0.001 | 0.013 |
| Household income-to-poverty  Ratio | -0.001 | 0.001 | 0.000 | 0.001 | 0.003 | 0.001 | 0.002 | 0.001 |
| Region | -0.001 | 0.002 | 0.000 | 0.003 | -0.005 | 0.004 | -0.004 | 0.003 |
| Reference year | 0.007 | 0.003 | 0.001 | 0.004 | 0.001 | 0.005 | 0.001 | 0.005 |
| Constant | 0.022 | 0.049 | -0.011 | 0.036 | 0.045 | 0.063 | 0.094 | 0.054 |
| Observations | 53,247 |  | 53,247 |  | 53,247 |  | 53,247 |  |
| Notes: Coefficients and standard errors shown. *p<.05 ** p<.01 *** p<.001  Source: Data from the Survey of Income and Program Participation’s Waves 2014 and 2018. | | | | | | | | |

# Supplementary Table 24. Multivariate linear probability regression estimates of the association between metropolitan status and food hardships using a composite food hardship scale.

|  | Model 1 | | Model 2 | | Model 3 | |
| --- | --- | --- | --- | --- | --- | --- |
|  | (Base) | | (Base + controls) | | (Full) | |
|  | b | se | b | se | b | se |
| Metropolitan status |  |  |  |  |  |  |
| Metropolitan | (Reference) | | | | | |
| Nonmetropolitan | 0.097*** | 0.021 | 0.048** | 0.015 | 0.009 | 0.014 |
| Householder characteristics |  |  |  |  |  |  |
| Age |  |  | 0.029*** | 0.002 | 0.032*** | 0.002 |
| Age^2^ |  |  | -0.000*** | 0.000 | -0.000*** | 0.000 |
| Gender |  |  |  |  |  |  |
| Men |  |  | (Reference) | | | |
| Women |  |  | 0.059*** | 0.007 | 0.045*** | 0.007 |
| Race and ethnicity |  |  |  |  |  |  |
| Non-Hispanic white |  |  | (Reference) | | | |
| Non-Hispanic Black |  |  | 0.115*** | 0.019 | 0.082*** | 0.02 |
| Hispanic |  |  | 0.052** | 0.015 | 0.021 | 0.016 |
| Asian |  |  | -0.054** | 0.017 | -0.060** | 0.018 |
| Other |  |  | 0.184*** | 0.038 | 0.161*** | 0.035 |
| Born in the U.S. |  |  | 0.001 | 0.021 | 0.047* | 0.02 |
| Education attainment |  |  |  |  |  |  |
| High school degree or less |  |  | (Reference) | | | |
| Some college |  |  | -0.080*** | 0.016 | -0.036* | 0.015 |
| Bachelor's degree |  |  | -0.255*** | 0.016 | -0.153*** | 0.014 |
| More than a bachelor's degree |  |  | -0.266*** | 0.015 | -0.145*** | 0.013 |
| Marital status |  |  |  |  |  |  |
| Never married |  |  | (Reference) | | | |
| Married |  |  | -0.155*** | 0.014 | -0.052*** | 0.014 |
| Divorced, separated, or widowed |  |  | 0.059** | 0.021 | 0.071*** | 0.02 |
| Employment status |  |  |  |  |  |  |
| Employed |  |  | (Reference) | | | |
| Unemployed |  |  | 0.583*** | 0.072 | 0.426*** | 0.068 |
| Not in labor force |  |  | 0.216*** | 0.017 | 0.079*** | 0.014 |
| Household characteristics |  |  |  |  |  |  |
| Housing tenure |  |  |  |  |  |  |
| Owner |  |  | (Reference) | | | |
| Renter |  |  | 0.312*** | 0.015 | 0.228*** | 0.013 |
| Occupy without payment of rent |  |  | 0.159** | 0.046 | 0.049 | 0.043 |
| Person with a disability present |  |  | 0.377*** | 0.011 | 0.339*** | 0.01 |
| Person 65 years or older present |  |  | -0.134*** | 0.013 | -0.097*** | 0.013 |
| Child present |  |  | 0.079*** | 0.014 | 0.021 | 0.014 |
| Number of persons present |  |  | -0.019** | 0.006 | -0.020*** | 0.005 |

Supplementary Table 24 (continued). Multivariate linear probability regression estimates of the association between metropolitan status and food hardships using a composite food hardship scale.

|  | Model 1 | | Model 2 | | Model 3 | |
| --- | --- | --- | --- | --- | --- | --- |
|  | (Base) | | (Base + controls) | | (Full) | |
|  | b | se | b | se | b | se |
| Household characteristics |  |  |  |  |  |  |
| Household income-to-poverty ratio |  |  |  |  |  |  |
| Less than 100% |  |  |  |  | (Reference) | |
| 100%-199% |  |  |  |  | -0.108*** | 0.025 |
| 200%-299% |  |  |  |  | -0.348*** | 0.021 |
| 300%-399% |  |  |  |  | -0.470*** | 0.023 |
| More than 400% |  |  |  |  | -0.560*** | 0.022 |
| Region |  |  |  |  |  |  |
| Northeast |  |  | (Reference) | | | |
| Midwest |  |  | 0.023 | 0.017 | 0.017 | 0.014 |
| South |  |  | 0.047*** | 0.012 | 0.024* | 0.011 |
| West |  |  | -0.008 | 0.017 | -0.003 | 0.014 |
| Year |  |  |  |  |  |  |
| 2013 |  |  | (Reference) | | | |
| 2017 |  |  | -0.088*** | 0.011 | -0.071*** | 0.011 |
| 2021 |  |  | -0.170*** | 0.011 | -0.146*** | 0.011 |
| Constant | 0.402*** | 0.012 | -0.197*** | 0.052 | 0.05 | 0.048 |
| Observations | 69735 |  | 69735 |  | 69735 |  |
| Coefficients and standard errors shown. *p<.05 ** p<.01 *** p<.001 | | | | | | |
| Source: Survey of Income and Program Participation Waves 2014, 2018, and 2022 | | | | | | |

# Supplementary Table 25. Multivariate linear probability regression estimates of the association between metropolitan status and housing quality hardship using a composite housing quality hardship scale.

|  | Model 1 | | Model 2 | | Model 3 | |
| --- | --- | --- | --- | --- | --- | --- |
|  | (Base) | | (Base + controls) | | (Full) | |
|  | b | se | b | se | b | se |
| Metropolitan status |  |  |  |  |  |  |
| Metropolitan | (Reference) | | | | | |
| Nonmetropolitan | 0.035** | 0.012 | 0.022* | 0.009 | 0.013 | 0.009 |
| Householder characteristics |  |  |  |  |  |  |
| Age |  |  | 0.006*** | 0.001 | 0.007*** | 0.001 |
| Age^2^ |  |  | -0.000*** | 0.000 | -0.000*** | 0.000 |
| Gender |  |  |  |  |  |  |
| Men |  |  | (Reference) | | | |
| Women |  |  | 0.007 | 0.005 | 0.004 | 0.006 |
| Race and ethnicity |  |  |  |  |  |  |
| Non-Hispanic white |  |  | (Reference) | | | |
| Non-Hispanic Black |  |  | 0.032* | 0.014 | 0.024 | 0.014 |
| Hispanic |  |  | 0.061** | 0.02 | 0.054** | 0.02 |
| Asian |  |  | 0.018 | 0.012 | 0.017 | 0.012 |
| Other |  |  | 0.112*** | 0.021 | 0.107*** | 0.021 |
| Born in the U.S. |  |  | 0.025* | 0.012 | 0.037** | 0.011 |
| Education attainment |  |  |  |  |  |  |
| High school degree or less |  |  | (Reference) | | | |
| Some college |  |  | -0.020** | 0.007 | -0.009 | 0.006 |
| Bachelor's degree |  |  | -0.055*** | 0.012 | -0.030** | 0.011 |
| More than a bachelor's degree |  |  | -0.052*** | 0.011 | -0.022* | 0.009 |
| Marital status |  |  |  |  |  |  |
| Never married |  |  | (Reference) | | | |
| Married |  |  | -0.099*** | 0.011 | -0.073*** | 0.011 |
| Divorced, separated, or widowed |  |  | 0 | 0.012 | 0.004 | 0.012 |
| Employment status |  |  |  |  |  |  |
| Employed |  |  | (Reference) | | | |
| Unemployed |  |  | 0.157*** | 0.025 | 0.118*** | 0.026 |
| Not in labor force |  |  | 0.054*** | 0.006 | 0.020*** | 0.005 |
| Household characteristics |  |  |  |  |  |  |
| Housing tenure |  |  |  |  |  |  |
| Owner |  |  | (Reference) | | | |
| Renter |  |  | 0.070*** | 0.015 | 0.049** | 0.016 |
| Occupy without payment of rent |  |  | 0.135*** | 0.023 | 0.108*** | 0.022 |
| Person with a disability present |  |  | 0.184*** | 0.009 | 0.175*** | 0.009 |
| Person 65 years or older present |  |  | -0.042** | 0.012 | -0.033** | 0.012 |
| Child present |  |  | -0.024* | 0.012 | -0.038** | 0.012 |
| Number of persons present |  |  | 0.017*** | 0.004 | 0.017*** | 0.004 |

Supplementary Table 25 (continued). Multivariate linear probability regression estimates of the association between metropolitan status and housing quality hardship using a composite housing quality hardship scale.

|  | Model 1 | | Model 2 | | Model 3 | |
| --- | --- | --- | --- | --- | --- | --- |
|  | (Base) | | (Base + controls) | | (Full) | |
|  | b | se | b | se | b | se |
| Household characteristics |  |  |  |  |  |  |
| Household income-to-poverty ratio |  |  |  |  |  |  |
| Less than 100% |  |  |  |  | (Reference) | |
| 100%-199% |  |  |  |  | -0.040** | 0.012 |
| 200%-299% |  |  |  |  | -0.086*** | 0.011 |
| 300%-399% |  |  |  |  | -0.111*** | 0.013 |
| More than 400% |  |  |  |  | -0.145*** | 0.013 |
| Region |  |  |  |  |  |  |
| Northeast |  |  | (Reference) | | | |
| Midwest |  |  | -0.043 | 0.022 | -0.045* | 0.022 |
| South |  |  | -0.007 | 0.026 | -0.013 | 0.026 |
| West |  |  | -0.028 | 0.024 | -0.027 | 0.024 |
| Year |  |  |  |  |  |  |
| 2013 |  |  | (Reference) | | | |
| 2017 |  |  | 0.013 | 0.006 | 0.017* | 0.007 |
| 2021 |  |  | -0.006 | 0.009 | 0 | 0.01 |
| Constant | 0.250*** | 0.01 | 0.073* | 0.03 | 0.138*** | 0.032 |
| Observations | 69735 |  | 69735 |  | 69735 |  |
| Coefficients and standard errors shown. *p<.05 ** p<.01 *** p<.001 | | | | | | |
| Source: Survey of Income and Program Participation Waves 2014, 2018, and 2022 | | | | | | |

# Supplementary Table 26. Multivariate linear probability regression estimates of the association between metropolitan status and all forms of hardship using a composite hardship scale.

|  | Model 1 | | Model 2 | | Model 3 | |
| --- | --- | --- | --- | --- | --- | --- |
|  | (Base) | | (Base + controls) | | (Full) | |
|  | b | se | b | se | b | se |
| Metropolitan status |  |  |  |  |  |  |
| Metropolitan | (Reference) | | | | | |
| Nonmetropolitan | 0.122*** | 0.031 | 0.061** | 0.021 | 0.002 | 0.019 |
| Householder characteristics |  |  |  |  |  |  |
| Age |  |  | 0.045*** | 0.002 | 0.049*** | 0.002 |
| Age^2^ |  |  | -0.001*** | 0.000 | -0.001*** | 0.000 |
| Gender |  |  |  |  |  |  |
| Men |  |  | (Reference) | | | |
| Women |  |  | 0.088*** | 0.011 | 0.068*** | 0.012 |
| Race and ethnicity |  |  |  |  |  |  |
| Non-Hispanic white |  |  | (Reference) | | | |
| Non-Hispanic Black |  |  | 0.248*** | 0.035 | 0.199*** | 0.036 |
| Hispanic |  |  | 0.163*** | 0.028 | 0.118*** | 0.028 |
| Asian |  |  | -0.064*** | 0.017 | -0.073*** | 0.018 |
| Other |  |  | 0.353*** | 0.06 | 0.319*** | 0.055 |
| Born in the U.S. |  |  | 0.025 | 0.024 | 0.094*** | 0.023 |
| Education attainment |  |  |  |  |  |  |
| High school degree or less |  |  | (Reference) | | | |
| Some college |  |  | -0.103*** | 0.022 | -0.038 | 0.019 |
| Bachelor's degree |  |  | -0.372*** | 0.026 | -0.219*** | 0.022 |
| More than a bachelor's degree |  |  | -0.388*** | 0.021 | -0.204*** | 0.018 |
| Marital status |  |  |  |  |  |  |
| Never married |  |  | (Reference) | | | |
| Married |  |  | -0.297*** | 0.022 | -0.142*** | 0.022 |
| Divorced, separated, or widowed |  |  | 0.081* | 0.031 | 0.099** | 0.03 |
| Employment status |  |  |  |  |  |  |
| Employed |  |  | (Reference) | | | |
| Unemployed |  |  | 0.929*** | 0.095 | 0.693*** | 0.091 |
| Not in labor force |  |  | 0.294*** | 0.018 | 0.088*** | 0.014 |
| Household characteristics |  |  |  |  |  |  |
| Housing tenure |  |  |  |  |  |  |
| Owner |  |  | (Reference) | | | |
| Renter |  |  | 0.457*** | 0.024 | 0.332*** | 0.023 |
| Occupy without payment of rent |  |  | 0.308*** | 0.066 | 0.143* | 0.061 |
| Person with a disability present |  |  | 0.666*** | 0.019 | 0.610*** | 0.017 |
| Person 65 years or older present |  |  | -0.211*** | 0.021 | -0.156*** | 0.02 |
| Child present |  |  | 0.105*** | 0.022 | 0.018 | 0.023 |
| Number of persons present |  |  | 0.008 | 0.009 | 0.006 | 0.008 |

Supplementary Table 26 (continued). Multivariate linear probability regression estimates of the association between metropolitan status and all forms of hardship using a composite hardship scale.

|  | Model 1 | | Model 2 | | Model 3 | |
| --- | --- | --- | --- | --- | --- | --- |
|  | (Base) | | (Base + controls) | | (Full) | |
|  | b | se | b | se | b | se |
| Household characteristics |  |  |  |  |  |  |
| Household income-to-poverty ratio |  |  |  |  |  |  |
| Less than 100% |  |  |  |  | (Reference) | |
| 100%-199% |  |  |  |  | -0.181*** | 0.029 |
| 200%-299% |  |  |  |  | -0.520*** | 0.026 |
| 300%-399% |  |  |  |  | -0.686*** | 0.032 |
| More than 400% |  |  |  |  | -0.849*** | 0.032 |
| Region |  |  |  |  |  |  |
| Northeast |  |  | (Reference) | | | |
| Midwest |  |  | -0.047 | 0.026 | -0.056* | 0.023 |
| South |  |  | 0.012 | 0.02 | -0.022 | 0.022 |
| West |  |  | -0.067* | 0.026 | -0.060* | 0.023 |
| Year |  |  |  |  |  |  |
| 2013 |  |  | (Reference) | | | |
| 2017 |  |  | -0.117*** | 0.012 | -0.090*** | 0.013 |
| 2021 |  |  | -0.231*** | 0.017 | -0.195*** | 0.017 |
| Constant | 0.792*** | 0.02 | -0.205** | 0.064 | 0.166* | 0.062 |
| Observations | 69735 |  | 69735 |  | 69735 |  |
| Coefficients and standard errors shown. *p<.05 ** p<.01 *** p<.001 | | | | | | |
| Source: Survey of Income and Program Participation Waves 2014, 2018, and 2022 | | | | | | |

# Supplementary Table 27. Blinder-Oaxaca decompositions of differences in the prevalence of material hardships across metropolitan and nonmetropolitan households using composite hardship scales.

|  | Food scale | | Housing quality scale | | All hardships | |
| --- | --- | --- | --- | --- | --- | --- |
|  | b | se | b | se | b | se |
| Metropolitan status |  |  |  |  |  |  |
| Metropolitan | 0.402*** | 0.011 | 0.250*** | 0.008 | 0.792*** | 0.018 |
| Nonmetropolitan | 0.499*** | 0.02 | 0.284*** | 0.014 | 0.914*** | 0.036 |
| *Difference* | -0.097*** | 0.02 | -0.035** | 0.012 | -0.122*** | 0.033 |
| *Explained* | -0.088*** | 0.013 | -0.022** | 0.007 | -0.120*** | 0.022 |
| Householder characteristics |  |  |  |  |  |  |
| Age | 0.021*** | 0.004 | 0.007*** | 0.001 | 0.032*** | 0.006 |
| Gender | -0.001* | 0.000 | 0.000 | 0.000 | -0.001* | 0.001 |
| Race and ethnicity | 0.001 | 0.003 | 0.006* | 0.003 | 0.014* | 0.005 |
| Born in the U.S. | -0.007* | 0.003 | -0.005** | 0.002 | -0.013** | 0.004 |
| Education attainment | -0.024*** | 0.002 | -0.004** | 0.002 | -0.034*** | 0.004 |
| Marital status | -0.004* | 0.002 | 0.000 | 0.001 | -0.006* | 0.003 |
| Employment status | -0.006*** | 0.002 | -0.002** | 0.001 | -0.007** | 0.002 |
| Household characteristics |  |  |  |  |  |  |
| Housing tenure | 0.023*** | 0.004 | 0.003 | 0.002 | 0.032*** | 0.006 |
| Person with a disability present | -0.038*** | 0.004 | -0.019*** | 0.002 | -0.067*** | 0.006 |
| Person 65 years or older present | 0.005*** | 0.001 | 0.002* | 0.001 | 0.008*** | 0.002 |
| Child present | 0.001 | 0.000 | -0.001* | 0.001 | 0.001 | 0.001 |
| Number of persons present | -0.002* | 0.001 | 0.002* | 0.001 | 0.001 | 0.001 |
| Household income-to-poverty ratio | -0.052*** | 0.006 | -0.013*** | 0.002 | -0.078*** | 0.009 |
| Region | -0.004 | 0.003 | 0.004 | 0.002 | 0.001 | 0.005 |
| Reference year | -0.001 | 0.001 | 0.000 | 0.000 | -0.002 | 0.002 |
| *Unexplained* | -0.009 | 0.014 | -0.013 | 0.011 | -0.002 | 0.021 |
| Householder characteristics |  |  |  |  |  |  |
| Age | -0.082 | 0.106 | -0.112 | 0.082 | -0.201 | 0.156 |
| Gender | -0.021 | 0.015 | 0.017* | 0.007 | -0.002 | 0.019 |
| Race and ethnicity | 0.013 | 0.025 | 0.009 | 0.015 | 0.009 | 0.037 |
| Born in the U.S. | -0.180*** | 0.044 | -0.003 | 0.031 | -0.207** | 0.07 |
| Education attainment | 0.004 | 0.007 | 0.004 | 0.005 | 0.016 | 0.01 |
| Marital status | -0.006 | 0.006 | 0.001 | 0.004 | -0.007 | 0.01 |
| Employment status | 0.063 | 0.044 | 0.011 | 0.018 | 0.068 | 0.057 |
| Household characteristics |  |  |  |  |  |  |
| Housing tenure | 0.019 | 0.023 | 0.015 | 0.015 | 0.022 | 0.031 |
| Person with a disability present | -0.027 | 0.015 | -0.01 | 0.01 | -0.04 | 0.026 |
| Person 65 years or older present | -0.004 | 0.01 | -0.009 | 0.01 | -0.011 | 0.016 |
| Child present | 0.02 | 0.013 | 0.01 | 0.007 | 0.031 | 0.017 |
| Number of persons present | -0.026 | 0.038 | -0.016 | 0.027 | -0.03 | 0.059 |
| Household income-to-poverty ratio | 0.000 | 0.003 | 0.004 | 0.002 | 0.002 | 0.004 |
| Region | -0.002 | 0.006 | -0.012 | 0.007 | -0.018 | 0.011 |
| Reference year | 0.023* | 0.011 | -0.001 | 0.01 | 0.026 | 0.018 |
| Constant | 0.198 | 0.131 | 0.079 | 0.116 | 0.342 | 0.229 |
| Observations | 69,735 |  | 69,735 |  | 69,735 |  |
| Notes: Coefficients and standard errors shown. *p<.05 ** p<.01 *** p<.001  Source: Data from the Survey of Income and Program Participation Waves 2014, 2018, and 2022. | | | | | | |

# Supplementary Table 28. Multivariate logistic regression estimates of the association between metropolitan status and food hardship.

|  | Model 1 | | Model 2 | | Model 3 | |
| --- | --- | --- | --- | --- | --- | --- |
|  | (Base) | | (Base + controls) | | (Full) | |
|  | b | se | b | se | b | se |
| Metropolitan status |  |  |  |  |  |  |
| Metropolitan | (Reference) | | | | | |
| Nonmetropolitan | 1.272*** | 0.068 | 1.154** | 0.055 | 1.029 | 0.046 |
| Householder characteristics |  |  |  |  |  |  |
| Age |  |  | 1.073*** | 0.006 | 1.087*** | 0.006 |
| Age^2^ |  |  | 0.999*** | 0 | 0.999*** | 0 |
| Gender |  |  |  |  |  |  |
| Men |  |  | (Reference) | | | |
| Women |  |  | 1.278*** | 0.03 | 1.233*** | 0.032 |
| Race and ethnicity |  |  |  |  |  |  |
| Non-Hispanic white |  |  | (Reference) | | | |
| Non-Hispanic Black |  |  | 1.345*** | 0.059 | 1.247*** | 0.058 |
| Hispanic |  |  | 1.225*** | 0.044 | 1.130** | 0.044 |
| Asian |  |  | 0.738*** | 0.062 | 0.705*** | 0.064 |
| Other |  |  | 1.460*** | 0.103 | 1.381*** | 0.095 |
| Born in the U.S. |  |  | 0.860* | 0.056 | 0.983 | 0.065 |
| Education attainment |  |  |  |  |  |  |
| High school degree or less |  |  | (Reference) | | | |
| Some college |  |  | 0.835*** | 0.032 | 0.93 | 0.035 |
| Bachelor's degree |  |  | 0.403*** | 0.02 | 0.553*** | 0.028 |
| More than a bachelor's degree |  |  | 0.283*** | 0.019 | 0.444*** | 0.03 |
| Marital status |  |  |  |  |  |  |
| Never married |  |  | (Reference) | | | |
| Married |  |  | 0.622*** | 0.025 | 0.841*** | 0.037 |
| Divorced, separated, or widowed |  |  | 1.199*** | 0.056 | 1.221*** | 0.057 |
| Employment status |  |  |  |  |  |  |
| Employed |  |  | (Reference) | | | |
| Unemployed |  |  | 2.596*** | 0.225 | 1.853*** | 0.159 |
| Not in labor force |  |  | 1.544*** | 0.059 | 1.081* | 0.04 |
| Household characteristics |  |  |  |  |  |  |
| Housing tenure |  |  |  |  |  |  |
| Owner |  |  | (Reference) | | | |
| Renter |  |  | 2.196*** | 0.087 | 1.760*** | 0.061 |
| Occupy without payment of rent |  |  | 1.574*** | 0.186 | 1.233 | 0.139 |
| Person with a disability present |  |  | 2.853*** | 0.093 | 2.593*** | 0.077 |
| Person 65 years or older present |  |  | 0.729*** | 0.036 | 0.792*** | 0.043 |
| Child present |  |  | 1.120** | 0.041 | 0.927* | 0.034 |
| Number of persons present |  |  | 0.968 | 0.016 | 0.969 | 0.015 |

Supplementary Table 28 (continued). Multivariate logistic regression estimates of the association between metropolitan status and food hardship.

|  | Model 1 | | Model 2 | | Model 3 | |
| --- | --- | --- | --- | --- | --- | --- |
|  | (Base) | | (Base + controls) | | (Full) | |
|  | b | se | b | se | b | se |
| Household characteristics |  |  |  |  |  |  |
| Household income-to-poverty ratio |  |  |  |  |  |  |
| Less than 100% |  |  |  |  | (Reference) | |
| 100%-199% |  |  |  |  | 0.991 | 0.039 |
| 200%-299% |  |  |  |  | 0.620*** | 0.025 |
| 300%-399% |  |  |  |  | 0.429*** | 0.026 |
| More than 400% |  |  |  |  | 0.180*** | 0.014 |
| Region |  |  |  |  |  |  |
| Northeast |  |  | (Reference) | | | |
| Midwest |  |  | 1.111 | 0.064 | 1.077 | 0.054 |
| South |  |  | 1.218*** | 0.06 | 1.142** | 0.049 |
| West |  |  | 1.02 | 0.065 | 1.04 | 0.054 |
| Reference year |  |  |  |  |  |  |
| 2013 |  |  | (Reference) | | | |
| 2017 |  |  | 0.750*** | 0.033 | 0.788*** | 0.034 |
| 2021 |  |  | 0.670*** | 0.028 | 0.720*** | 0.03 |
| Observations | 69,735 |  | 69,735 |  | 69,735 |  |
| Notes: Coefficients and standard errors shown. *p<.05 ** p<.01 *** p<.001 | | | | | | |
| Source: Survey of Income and Program Participation Waves 2014, 2018, and 2022 | | | | | | |

# Supplementary Table 29. Multivariate logistic regression estimates of the association between metropolitan status and bill-paying hardship.

|  | Model 1 | | Model 2 | | Model 3 | |
| --- | --- | --- | --- | --- | --- | --- |
|  | (Base) | | (Base + controls) | | (Full) | |
|  | b | se | b | se | b | se |
| Metropolitan status |  |  |  |  |  |  |
| Metropolitan | (Reference) | | | | | |
| Nonmetropolitan | 0.999 | 0.045 | 0.986 | 0.051 | 0.901* | 0.044 |
| Householder characteristics |  |  |  |  |  |  |
| Age |  |  | 1.091*** | 0.01 | 1.102*** | 0.011 |
| Age^2^ |  |  | 0.999*** | 0 | 0.999*** | 0 |
| Gender |  |  |  |  |  |  |
| Men |  |  | (Reference) | | | |
| Women |  |  | 1.227*** | 0.041 | 1.193*** | 0.04 |
| Race and ethnicity |  |  |  |  |  |  |
| Non-Hispanic white |  |  | (Reference) | | | |
| Non-Hispanic Black |  |  | 1.932*** | 0.106 | 1.829*** | 0.102 |
| Hispanic |  |  | 1.471*** | 0.084 | 1.386*** | 0.076 |
| Asian |  |  | 0.790** | 0.065 | 0.771** | 0.066 |
| Other |  |  | 1.614*** | 0.149 | 1.553*** | 0.134 |
| Born in the U.S. |  |  | 0.997 | 0.052 | 1.104 | 0.065 |
| Education attainment |  |  |  |  |  |  |
| High school degree or less |  |  | (Reference) | | | |
| Some college |  |  | 0.983 | 0.035 | 1.07 | 0.038 |
| Bachelor's degree |  |  | 0.524*** | 0.037 | 0.670*** | 0.045 |
| More than a bachelor's degree |  |  | 0.410*** | 0.03 | 0.571*** | 0.046 |
| Marital status |  |  |  |  |  |  |
| Never married |  |  | (Reference) | | | |
| Married |  |  | 0.720*** | 0.035 | 0.900* | 0.041 |
| Divorced, separated, or widowed |  |  | 1.257*** | 0.061 | 1.276*** | 0.06 |
| Employment status |  |  |  |  |  |  |
| Employed |  |  | (Reference) | | | |
| Unemployed |  |  | 2.344*** | 0.157 | 1.811*** | 0.12 |
| Not in labor force |  |  | 1.117** | 0.039 | 0.859*** | 0.032 |
| Household characteristics |  |  |  |  |  |  |
| Housing tenure |  |  |  |  |  |  |
| Owner |  |  | (Reference) | | | |
| Renter |  |  | 1.599*** | 0.072 | 1.347*** | 0.057 |
| Occupy without payment of rent |  |  | 1.264 | 0.165 | 1.049 | 0.131 |
| Person with a disability present |  |  | 2.434*** | 0.075 | 2.263*** | 0.072 |
| Person 65 years or older present |  |  | 0.776*** | 0.038 | 0.828*** | 0.04 |
| Child present |  |  | 1.444*** | 0.058 | 1.271*** | 0.05 |
| Number of persons present |  |  | 1.035* | 0.015 | 1.031* | 0.014 |

*Supplementary Table 29 (continued). Multivariate logistic regression estimates of the association between metropolitan status and bill-paying hardship.*

|  | Model 1 | | Model 2 | | Model 3 | |
| --- | --- | --- | --- | --- | --- | --- |
|  | (Base) | | (Base + controls) | | (Full) | |
|  | b | se | b | se | b | se |
| Household characteristics |  |  |  |  |  |  |
| Household income-to-poverty ratio |  |  |  |  |  |  |
| Less than 100% |  |  |  |  | (Reference) | |
| 100%-199% |  |  |  |  | 0.919 | 0.042 |
| 200%-299% |  |  |  |  | 0.683*** | 0.031 |
| 300%-399% |  |  |  |  | 0.544*** | 0.034 |
| More than 400% |  |  |  |  | 0.297*** | 0.027 |
| Region |  |  |  |  |  |  |
| Northeast |  |  | (Reference) | | | |
| Midwest |  |  | 0.823*** | 0.03 | 0.798*** | 0.031 |
| South |  |  | 0.792*** | 0.029 | 0.749*** | 0.032 |
| West |  |  | 0.793*** | 0.034 | 0.801*** | 0.04 |
| Reference year |  |  |  |  |  |  |
| 2013 |  |  | (Reference) | | | |
| 2017 |  |  | 0.736*** | 0.034 | 0.765*** | 0.038 |
| 2021 |  |  | 0.636*** | 0.035 | 0.672*** | 0.037 |
| Observations | 69,735 |  | 69,735 |  | 69,735 |  |
| Notes: Coefficients and standard errors shown. *p<.05 ** p<.01 *** p<.001 | | | | | | |
| Source: Survey of Income and Program Participation Waves 2014, 2018, and 2022 | | | | | | |

# Supplementary Table 30. Multivariate logistic regression estimates of the association between metropolitan status and housing hardship.

|  | Model 1 | | Model 2 | | Model 3 | |
| --- | --- | --- | --- | --- | --- | --- |
|  | (Base) | | (Base + controls) | | (Full) | |
|  | b | se | b | se | b | se |
| Metropolitan status |  |  |  |  |  |  |
| Metropolitan | (Reference) | | | | | |
| Nonmetropolitan | 1.101* | 0.049 | 1.066 | 0.039 | 1.028 | 0.038 |
| Householder characteristics |  |  |  |  |  |  |
| Age |  |  | 1.016*** | 0.004 | 1.019*** | 0.004 |
| Age^2^ |  |  | 1.000*** | 0 | 1.000*** | 0 |
| Gender |  |  |  |  |  |  |
| Men |  |  | (Reference) | | | |
| Women |  |  | 1.055* | 0.024 | 1.042 | 0.025 |
| Race and ethnicity |  |  |  |  |  |  |
| Non-Hispanic white |  |  | (Reference) | | | |
| Non-Hispanic Black |  |  | 1.154** | 0.061 | 1.125* | 0.06 |
| Hispanic |  |  | 1.247** | 0.093 | 1.215* | 0.089 |
| Asian |  |  | 1.141 | 0.082 | 1.135 | 0.082 |
| Other |  |  | 1.517*** | 0.111 | 1.495*** | 0.109 |
| Born in the U.S. |  |  | 1.08 | 0.053 | 1.126* | 0.057 |
| Education attainment |  |  |  |  |  |  |
| High school degree or less |  |  | (Reference) | | | |
| Some college |  |  | 0.953 | 0.026 | 0.987 | 0.026 |
| Bachelor's degree |  |  | 0.796*** | 0.045 | 0.876* | 0.046 |
| More than a bachelor's degree |  |  | 0.827** | 0.05 | 0.938 | 0.051 |
| Marital status |  |  |  |  |  |  |
| Never married |  |  | (Reference) | | | |
| Married |  |  | 0.672*** | 0.029 | 0.736*** | 0.034 |
| Divorced, separated, or widowed |  |  | 1.019 | 0.041 | 1.026 | 0.041 |
| Employment status |  |  |  |  |  |  |
| Employed |  |  | (Reference) | | | |
| Unemployed |  |  | 1.494*** | 0.092 | 1.324*** | 0.088 |
| Not in labor force |  |  | 1.139*** | 0.022 | 1.014 | 0.018 |
| Household characteristics |  |  |  |  |  |  |
| Housing tenure |  |  |  |  |  |  |
| Owner |  |  | (Reference) | | | |
| Renter |  |  | 1.319*** | 0.064 | 1.227*** | 0.064 |
| Occupy without payment of rent |  |  | 1.546*** | 0.099 | 1.419*** | 0.092 |
| Person with a disability present |  |  | 2.158*** | 0.057 | 2.089*** | 0.056 |
| Person 65 years or older present |  |  | 0.870** | 0.04 | 0.894* | 0.041 |
| Child present |  |  | 0.916 | 0.04 | 0.866** | 0.041 |
| Number of persons present |  |  | 1.069*** | 0.012 | 1.069*** | 0.012 |

Supplementary Table 30 (continued). Multivariate logistic regression estimates of the association between metropolitan status and housing hardship.

|  | Model 1 | | Model 2 | | Model 3 | |
| --- | --- | --- | --- | --- | --- | --- |
|  | (Base) | | (Base + controls) | | (Full) | |
|  | b | se | b | se | b | se |
| Household characteristics |  |  |  |  |  |  |
| Household income-to-poverty  ratio |  |  |  |  |  |  |
| Less than 100% |  |  |  |  | (Reference) | |
| 100%-199% |  |  |  |  | 0.936 | 0.037 |
| 200%-299% |  |  |  |  | 0.832*** | 0.032 |
| 300%-399% |  |  |  |  | 0.754*** | 0.039 |
| More than 400% |  |  |  |  | 0.615*** | 0.031 |
| Region |  |  |  |  |  |  |
| Northeast |  |  | (Reference) | | | |
| Midwest |  |  | 0.832* | 0.066 | 0.824* | 0.066 |
| South |  |  | 0.945 | 0.09 | 0.924 | 0.09 |
| West |  |  | 0.919 | 0.076 | 0.924 | 0.078 |
| Reference year |  |  |  |  |  |  |
| 2013 |  |  | (Reference) | | | |
| 2017 |  |  | 1.053 | 0.032 | 1.071* | 0.034 |
| 2021 |  |  | 0.962 | 0.039 | 0.985 | 0.041 |
| Observations | 69,735 |  | 69,735 |  | 69,735 |  |
| Coefficients and standard errors shown. *p<.05 ** p<.01 *** p<.001 | | | | | | |
| Source: Survey of Income and Program Participation Waves 2014, 2018, and 2022 | | | | | | |

# Supplementary Table 31. Multivariate logistic regression estimates of the association between metropolitan status and any form of hardship.

|  | Model 1 | | Model 2 | | Model 3 | |
| --- | --- | --- | --- | --- | --- | --- |
|  | (Base) | | (Base + controls) | | (Full) | |
|  | b | se | b | se | b | se |
| Metropolitan status |  |  |  |  |  |  |
| Metropolitan | (Reference) | | | | | |
| Nonmetropolitan | 1.104* | 0.045 | 1.068* | 0.034 | 0.995 | 0.03 |
| Householder characteristics |  |  |  |  |  |  |
| Age |  |  | 1.047*** | 0.004 | 1.055*** | 0.004 |
| Age^2^ |  |  | 0.999*** | 0 | 0.999*** | 0 |
| Gender |  |  |  |  |  |  |
| Men |  |  | (Reference) | | | |
| Women |  |  | 1.159*** | 0.021 | 1.133*** | 0.023 |
| Race and ethnicity |  |  |  |  |  |  |
| Non-Hispanic white |  |  | (Reference) | | | |
| Non-Hispanic Black |  |  | 1.445*** | 0.063 | 1.382*** | 0.064 |
| Hispanic |  |  | 1.347*** | 0.06 | 1.283*** | 0.055 |
| Asian |  |  | 0.979 | 0.046 | 0.967 | 0.047 |
| Other |  |  | 1.670*** | 0.128 | 1.632*** | 0.12 |
| Born in the U.S. |  |  | 0.991 | 0.025 | 1.073* | 0.031 |
| Education attainment |  |  |  |  |  |  |
| High school degree or less |  |  | (Reference) | | | |
| Some college |  |  | 0.915** | 0.027 | 0.98 | 0.028 |
| Bachelor's degree |  |  | 0.621*** | 0.033 | 0.744*** | 0.038 |
| More than a bachelor's degree |  |  | 0.594*** | 0.029 | 0.750*** | 0.034 |
| Marital status |  |  |  |  |  |  |
| Never married |  |  | (Reference) | | | |
| Married |  |  | 0.640*** | 0.023 | 0.760*** | 0.028 |
| Divorced, separated, or widowed |  |  | 1.115** | 0.044 | 1.133** | 0.043 |
| Employment status |  |  |  |  |  |  |
| Employed |  |  | (Reference) | | | |
| Unemployed |  |  | 2.180*** | 0.168 | 1.741*** | 0.132 |
| Not in labor force |  |  | 1.283*** | 0.028 | 1.031 | 0.02 |
| Household characteristics |  |  |  |  |  |  |
| Housing tenure |  |  |  |  |  |  |
| Owner |  |  | (Reference) | | | |
| Renter |  |  | 1.673*** | 0.053 | 1.466*** | 0.046 |
| Occupy without payment of rent |  |  | 1.542*** | 0.101 | 1.318*** | 0.086 |
| Person with a disability present |  |  | 2.475*** | 0.066 | 2.347*** | 0.066 |
| Person 65 years or older present |  |  | 0.804*** | 0.025 | 0.845*** | 0.027 |
| Child present |  |  | 1.117*** | 0.033 | 1.003 | 0.031 |
| Number of persons present |  |  | 1.040*** | 0.01 | 1.039*** | 0.01 |

Supplementary Table 31 (continued). Multivariate logistic regression estimates of the association between metropolitan status and any form of hardship.

|  | Model 1 | | Model 2 | | Model 3 | |
| --- | --- | --- | --- | --- | --- | --- |
|  | (Base) | | (Base + controls) | | (Full) | |
|  | b | se | b | se | b | se |
| Household characteristics |  |  |  |  |  |  |
| Household income-to-poverty ratio |  |  |  |  |  |  |
| Less than 100% |  |  |  |  | (Reference) | |
| 100%-199% |  |  |  |  | 0.918* | 0.033 |
| 200%-299% |  |  |  |  | 0.705*** | 0.019 |
| 300%-399% |  |  |  |  | 0.572*** | 0.028 |
| More than 400% |  |  |  |  | 0.411*** | 0.02 |
| Region |  |  |  |  |  |  |
| Northeast |  |  | (Reference) | | | |
| Midwest |  |  | 0.861** | 0.045 | 0.844** | 0.046 |
| South |  |  | 0.924 | 0.052 | 0.885 | 0.056 |
| West |  |  | 0.9 | 0.051 | 0.905 | 0.054 |
| Reference year |  |  |  |  |  |  |
| 2013 |  |  | (Reference) | | | |
| 2017 |  |  | 0.911** | 0.03 | 0.939 | 0.032 |
| 2021 |  |  | 0.817*** | 0.028 | 0.853*** | 0.029 |
| Observations | 69,735 |  | 69,735 |  | 69,735 |  |
| Coefficients and standard errors shown. *p<.05 ** p<.01 *** p<.001 | | | | | | |
| Source: Survey of Income and Program Participation Waves 2014, 2018, and 2022 | | | | | | |

# Supplementary Table 32. Blinder-Oaxaca decompositions of differences in the prevalence of material hardships across metropolitan and nonmetropolitan households using logistic regression.

|  | Food | | Bill-paying | | Housing | | Any Hardship | |
| --- | --- | --- | --- | --- | --- | --- | --- | --- |
|  | b | se | b | se | b | se | b | se |
| Metropolitan status |  |  |  |  |  |  |  |  |
| Metropolitan | 0.108*** | 0.003 | 0.101*** | 0.003 | 0.173*** | 0.005 | 0.278*** | 0.005 |
| Nonmetropolitan | 0.134*** | 0.006 | 0.101*** | 0.005 | 0.187*** | 0.007 | 0.298*** | 0.008 |
| *Difference* | -0.026*** | 0.006 | 0.000 | 0.005 | -0.014* | 0.007 | -0.020* | 0.008 |
| *Explained* | -0.023*** | 0.004 | -0.009** | 0.003 | -0.010* | 0.004 | -0.021*** | 0.005 |
| Householder characteristics |  |  |  |  |  |  |  |  |
| Age | 0.005*** | 0.001 | 0.004*** | 0.001 | 0.004*** | 0.001 | 0.008*** | 0.001 |
| Gender | -0.000* | 0 | -0.000* | 0.000 | 0.000 | 0.000 | -0.000* | 0.000 |
| Race and ethnicity | 0.000 | 0.001 | 0.002** | 0.001 | 0.004* | 0.002 | 0.006*** | 0.002 |
| Born in the U.S. | 0.000 | 0.001 | -0.001 | 0.001 | -0.002* | 0.001 | -0.002* | 0.001 |
| Highest education attainment | -0.008*** | 0.001 | -0.005*** | 0.001 | -0.002* | 0.001 | -0.009*** | 0.001 |
| Marital status | -0.001* | 0.000 | -0.001** | 0.000 | 0.000 | 0.001 | -0.001 | 0.001 |
| Employment status | 0.000 | 0.000 | 0.001*** | 0.000 | 0.000 | 0.000 | 0.000 | 0.000 |
| Household characteristics |  |  |  |  |  |  |  |  |
| Housing tenure | 0.004*** | 0.001 | 0.002*** | 0 | 0.002* | 0.001 | 0.006*** | 0.001 |
| Person with a disability present | -0.007*** | 0.001 | -0.005*** | 0.001 | -0.012*** | 0.001 | -0.017*** | 0.002 |
| Person 65 years or older present | 0.001** | 0.000 | 0.001** | 0.000 | 0.001* | 0.000 | 0.001*** | 0.000 |
| Child present | -0.000 | 0.000 | 0.000** | 0.000 | -0.001* | 0.000 | 0.000 | 0.000 |
| Number of persons present | 0 | 0.000 | 0.000+ | 0.000 | 0.001** | 0.000 | 0.001* | 0.000 |
| Household income-to-poverty ratio | -0.014*** | 0.002 | -0.008*** | 0.002 | -0.007*** | 0.001 | -0.017*** | 0.002 |
| Region | -0.001 | 0.001 | 0.001 | 0.001 | 0.003* | 0.001 | 0.003* | 0.001 |
| Year | 0.000 | 0.000 | 0.000 | 0.000 | 0.000 | 0.000 | 0.000 | 0.000 |
| *Unexplained* | -0.003 | 0.004 | 0.009* | 0.004 | -0.004 | 0.006 | 0.001 | 0.006 |
| Householder characteristics |  |  |  |  |  |  |  |  |
| Age | 0.002 | 0.007 | -0.008 | 0.015 | 1.588 | 71.906 | -0.008 | 0.049 |
| Gender | 0.001 | 0.003 | 0.002 | 0.002 | -0.162 | 7.332 | 0 | 0.001 |
| Race and ethnicity | -0.001 | 0.002 | -0.004 | 0.004 | 0.178 | 8.114 | -0.001 | 0.003 |
| Born in the U.S. | 0.009 | 0.015 | -0.008 | 0.009 | -0.141 | 6.417 | -0.004 | 0.021 |
| Highest education attainment | 0.000 | 0.001 | 0.003 | 0.002 | -0.028 | 1.285 | 0.001 | 0.003 |
| Marital status | 0.000 | 0.001 | 0.000 | 0.001 | -0.065 | 2.942 | 0.000 | 0.001 |
| Employment status | -0.001 | 0.002 | -0.001 | 0.002 | -0.258 | 11.751 | 0.000 | 0.001 |
| Household characteristics |  |  |  |  |  |  |  |  |
| Housing tenure | 0.000 | 0.001 | -0.006+ | 0.003 | 0.003 | 0.195 | 0.000 | 0.001 |
| Person with a disability present | 0.001 | 0.002 | -0.003 | 0.002 | 0.045 | 2.041 | -0.001 | 0.004 |
| Person 65 years or older present | 0.001 | 0.002 | 0.000 | 0.002 | 0.154 | 6.948 | -0.001 | 0.003 |
| Child present | -0.001 | 0.001 | 0.000 | 0.001 | -0.064 | 2.857 | 0.000 | 0.001 |
| Number of persons present | 0.000 | 0.002 | 0.002 | 0.004 | 0.154 | 6.911 | 0.000 | 0.001 |
| Household income-to-poverty ratio | 0.000 | 0.001 | 0.001 | 0.001 | -0.076 | 3.457 | 0.000 | 0.002 |
| Region | 0.000 | 0.001 | -0.001 | 0.001 | 0.158 | 7.233 | -0.001 | 0.003 |
| Year | -0.001 | 0.002 | 0.001 | 0.002 | 0.006 | 0.262 | 0.000 | 0.001 |
| Constant | -0.013 | 0.025 | 0.032 | 0.021 | -1.497 | 67.481 | 0.014 | 0.08 |
| Observations | 69,735 |  | 69,735 |  | 69,735 |  | 69,735 |  |
| Note: Coefficients and standard errors shown. *p<.05 ** p<.01 *** p<.001  Source: Survey of Income and Program Participation (SIPP) waves 2014, 2018, and 2022 | | | | | | | | |

# Supplementary Table 33. Fairlie decompositions of differences in the prevalence of material hardships across metropolitan and nonmetropolitan households.

|  | Food | | Bill-paying | | Housing quality | | Any hardship | |
| --- | --- | --- | --- | --- | --- | --- | --- | --- |
|  | b | se | b | se | b | se | b | se |
| Metropolitan status |  |  |  |  |  |  |  |  |
| Metropolitan | 0.108 |  | 0.101 |  | 0.173 |  | 0.278 |  |
| Nonmetropolitan | 0.134 |  | 0.101 |  | 0.187 |  | 0.298 |  |
| *Difference* | -0.0255 |  | 0.000 |  | -0.0141 |  | -0.02 |  |
| *Difference explained* | -0.023 |  | -0.008 |  | -0.011 |  | -0.021 |  |
| Householder characteristics |  |  |  |  |  |  |  |  |
| Age | 0.004*** | 0.001 | 0.002*** | 0.001 | 0.003*** | 0.001 | 0.006*** | 0.001 |
| Gender | -0.000* | 0.000 | -0.000* | 0.000 | 0.000 | 0.000 | -0.000*** | 0.000 |
| Race and ethnicity | 0.001 | 0.001 | 0.006*** | 0.001 | 0.004*** | 0.001 | 0.007*** | 0.001 |
| Born in the U.S. | 0.000 | 0.001 | -0.001 | 0.001 | -0.002** | 0.001 | -0.002* | 0.001 |
| Education attainment | -0.006*** | 0.000 | -0.004*** | 0.000 | -0.002*** | 0.001 | -0.007*** | 0.001 |
| Marital status | -0.002*** | 0.000 | -0.001*** | 0.000 | 0.000 | 0.000 | -0.001** | 0.000 |
| Employment status | -0.001 | 0.000 | 0.001** | 0.000 | 0.000 | 0.000 | 0.000 | 0.001 |
| Household characteristics |  |  |  |  |  |  |  |  |
| Housing tenure | 0.006*** | 0.000 | 0.003*** | 0.000 | 0.002*** | 0.001 | 0.007*** | 0.001 |
| Person with a disability present | -0.011*** | 0.001 | -0.008*** | 0.001 | -0.011*** | 0.001 | -0.018*** | 0.001 |
| Person 65 years or older present | 0.001 | 0.000 | 0.000 | 0.000 | 0.001 | 0.000 | 0.001** | 0.000 |
| Child present | 0.000 | 0.000 | 0.001* | 0.000 | -0.000* | 0.000 | 0.000 | 0.000 |
| Number of persons present | 0.000 | 0.000 | 0.000 | 0.000 | 0.001*** | 0.000 | 0.001** | 0.000 |
| Household income-to-poverty ratio | -0.013*** | 0.001 | -0.010*** | 0.001 | -0.007*** | 0.001 | -0.017*** | 0.001 |
| Region | -0.001* | 0.001 | 0.002** | 0.001 | 0.003*** | 0.001 | 0.003*** | 0.001 |
| Reference year | 0.000 | 0.000 | 0.000 | 0.000 | 0.000 | 0.000 | -0.000** | 0.000 |
| Observations | 69,717 |  | 69,717 |  | 69,717 |  | 69,717 |  |
| Notes: Coefficients and standard errors shown. *p<.05** p<.01 *** p<.001  Source: Data from the Survey of Income and Program Participation Waves 2014, 2018, and 2022. | | | | | | | | |
